# Specific Gene Expression Responses to Parasite Genotypes Reveal Redundancy of Innate Immunity in Vertebrates

**DOI:** 10.1371/journal.pone.0108001

**Published:** 2014-09-25

**Authors:** David Haase, Jennifer K. Rieger, Anika Witten, Monika Stoll, Erich Bornberg-Bauer, Martin Kalbe, Thorsten B. H. Reusch

**Affiliations:** 1 Evolutionary Ecology of Marine Fishes, GEOMAR Helmholtz Centre for Ocean Research Kiel, Kiel, Germany; 2 Genetic Epidemiology of Vascular Disorders, Leibniz Institute for Arteriosclerosis Research at the University Münster, Münster, Germany; 3 Institute for Evolution and Biodiversity, University of Münster, Münster, Germany; 4 Department Evolutionary Ecology, Max-Planck Institute for Evolutionary Biology, Plön, Germany; Chang Gung University, Taiwan

## Abstract

Vertebrate innate immunity is the first line of defense against an invading pathogen and has long been assumed to be largely unspecific with respect to parasite/pathogen species. However, recent phenotypic evidence suggests that immunogenetic variation, i.e. allelic variability in genes associated with the immune system, results in host-parasite genotype-by-genotype interactions and thus specific innate immune responses. Immunogenetic variation is common in all vertebrate taxa and this reflects an effective immunological function in complex environments. However, the underlying variability in host gene expression patterns as response of innate immunity to within-species genetic diversity of macroparasites in vertebrates is unknown. We hypothesized that intra-specific variation among parasite genotypes must be reflected in host gene expression patterns. Here we used high-throughput RNA-sequencing to examine the effect of parasite genotypes on gene expression patterns of a vertebrate host, the three-spined stickleback (*Gasterosteus aculeatus*). By infecting naïve fish with distinct trematode genotypes of the species *Diplostomum pseudospathaceum* we show that gene activity of innate immunity in three-spined sticklebacks depended on the identity of an infecting macroparasite genotype. In addition to a suite of genes indicative for a general response against the trematode we also find parasite-strain specific gene expression, in particular in the complement system genes, despite similar infection rates of single clone treatments. The observed discrepancy between infection rates and gene expression indicates the presence of alternative pathways which execute similar functions. This suggests that the innate immune system can induce redundant responses specific to parasite genotypes.

## Introduction

Vertebrate immunity consists of innate and adaptive components, intertwined through a close interaction of both systems. Innate immunity has long been considered rather unspecific with respect to the pathogen species, let alone the genotype of a particular pathogen or parasite [1]. However, the distinction between an unspecific innate immune response and a specific adaptive response is not clear-cut [2]. For example, the variable activation of innate receptor types may be induced by multiple signaling pathways which result in a wide range of possible immune responses [3]. Furthermore, immunogenetic variation, i.e. allelic variability in genes associated with the immune system, is common in all vertebrate taxa and this reflects an effective immunological function in complex environments (reviewed in Maizels & Nussey 2013 [4]), but it is unclear how this mediates divergent immune response of hosts with respect to the effector cascades. We also have little knowledge on the degree of discrimination that the innate immune system can achieve, although some phenotypic studies suggest distinction among parasite genotypes in three-spined sticklebacks, rainbow trout and monarch butterflies [5] [6] [7], but also in a wide range of other species as reviewed by Lazzaro & Little [8]. In line with these findings, host immune reactions were found to be parasite-genotype specific in crabs [9] and gene expression differences could be attributed to host-parasite genotype interactions in bumblebees [10]. But the genes responsible for host immune reactions to parasite genotypes have yet to be shown in vertebrates.

In this study we used a member of the bony fishes, the three-spined stickleback (*Gasterosteus aculeatus*), a recently emerging model fish species with outstanding genomic and transcriptomic resources [11] [12] [13]. Bony fish are a basal class of vertebrates and are among the first taxa which unite elements of innate and adaptive immunity, making them a cornerstone for further understanding the evolution of basic features in vertebrate immune responses [14] [15]. Studies on host responses against pathogenic viruses have already shown that the main mechanisms of both innate and adaptive immunity in bony fish are similar to those in mammals [16]. The presence of intra-specific parasite effects however suggests a currently unknown genetic basis for variation of the innate immune response in bony fishes [5]. The understanding of such genotype specific effects and alternative immune response pathways can provide information about the specific characteristics of the discrimination processes in vertebrates.

The model parasite *Diplostomum pseudospathaceum*, a digenean trematode, was utilized to elicit a parasite genotype specific innate immune response in the fish host. This parasite has a complex life cycle, using freshwater snails and fish as intermediate hosts, before reproducing sexually in piscivorous birds [17]. Since digenean trematodes undergo clonal expansion in the snail host, they are ideal candidates to investigate genotype-specific performance of parasites [9]. After penetration of the skin and migration through tissues and blood vessels, larvae of *D. pseudospathaceum* invade the fish’s eye lens within 24 h to evade the adaptive immune system [17]. Higher infection intensities of the parasite reduce the visual capacities of its fish host, impending feeding efficiency and predator avoidance [18] [19]. The short timeframe between initial penetration and invasion of the eye facilitates the innate immune system as key response to *D. pseudospathaceum* in naive fish [20].

Here we investigated host gene expression patterns of four stickleback families with regard to the general parasite species mediated effect but were particularly interested in unique trematode genotype-dependent effects. We examined the mRNA expression patterns in head kidneys, the principal immune organ in fish [21], and gills, one of the preferred spots for penetration of parasite larvae [22]. Gene expression differences can be affected by natural selection [23], leading to habitat specific immune responses [24] and show parasite genotype specific host responses [10]. To check for gene expression differences, we compared gene expression patterns of exposed fish to naive controls. First we assessed all genes differentially expressed as a function of parasite treatment to investigate how a general transcriptomic response against *D. pseudospathaceum* in three-spined sticklebacks is realized. Here we expected a set of genes to be differentially expressed in all fish treated with *D. pseudospathaceum*. We then compared the genes uniquely expressed due to a specific monoclonal treatment, investigating the specificity of a transcriptomic response against a certain parasite genotype and how this influences gene expression patterns. Furthermore, we focussed on the gene expression levels of known host immune genes. Here we reduced the amount of differentially expressed genes to a set of prior defined immune relevant genes in *G. aculeatus*. With this approach we expected to identify a distinct subset of immune genes putatively responsible as key elements against infections with the parasitic trematode *D. pseudospathaceum* on the gene expression level.

## Results

### Parasite load

**Figure 1:**
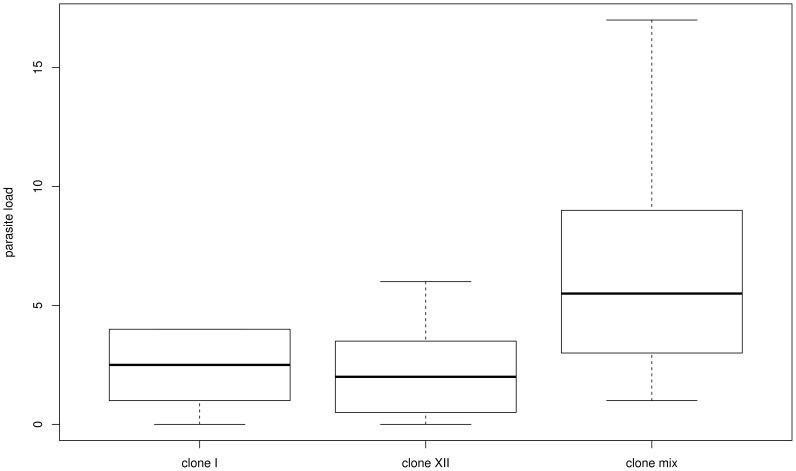
Effects of parasite exposure to parasite load in sticklebacks. Shown is parasite load of three-spined sticklebacks, exposed to three different treatments of *Diplostomum pseudospathaceum* cercariae; clone I, clone XII and clone mix. The box plots show distribution of total number of cercaria per fish in each infection treatment.

Parasite load of infected sticklebacks ranged from 0–4 metacercariae (larvae) in the eye lenses for *D. pseudospathaceum*-clone I, 0–6 for clone XII and 1–17 for the clone mix treatment (figure 1). Infection intensity varied significantly among parasite treatments (ANOVA, F = 9.595, p = 0.000867). Fish family as main factor and fish family x treatment interactions had no significant effect on parasite infection success. Differences in parasite infection success were driven by clone mix vs. single clone treatments (post-hoc comparison p = 0.0056 against clone I, p = 0.0034 against clone XII) while there was no significant distinction between both single *D. pseudospathaceum* clones (p = 0.98).

### Differential gene expression

**Figure 2:**
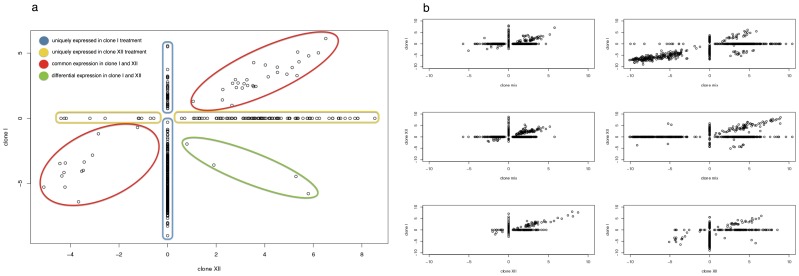
Treatment specific pairwise comparisons of host gene expression. (a) Exemplary comparison of log2-fold change values of genes differentially expressed in head kidney tissue of the clone I and XII treatments. Genes with fold changes only significant in one of two treatments are set to zero in the other treatment, thus genes can be either identified as unique to a certain treatment (yellow, blue), similar in both treatments (red) or up-regulated in one treatment while down-regulated in the other (green). (b) Figure 2b shows comparison of log2-fold changes for all pairwise treatment comparisons. From top to bottom, clone I vs. mix, clone XII vs. mix and clone I vs. XII, with gill tissue samples in the left column and head kidney samples in the right column. Differential expression is defined a statistical significance in differences of gene expression between exposed and control fish.

Illumina RNA sequencing produced 29 good-quality libraries representing samples of head kidney and gills from single fish individuals (total data set 486 million valid paired-end reads of 101 nucleotides length). This excludes two samples from gills (clone I, control) and one sample from head kidneys (control) which had to be removed because they did not meet sequence quality or read number criteria. A total of 265 million reads with an average distribution of ∼9 million reads per sample were aligned with Tophat (table S1). Fold-changes were calculated with Cufflinks for each gene of any possible combination of two treatments for all 4 treatments, of which 37945 were significant in their gene expression differences. We focused on the comparison of infection treatments with the tissue specific uninfected controls, resulting in 1415 (gill) and 1060 (head kidney) gene comparisons to be significantly different, which comprise of 1246 unique genes in gill and 691 in head kidney tissue (table S2 and S3). In addition we were able to detect parasite genotype dependent differences in host gene expression-levels (figure 2, table S6 and S7).

### GO term enrichment

GO term enrichment analysis of the full gene set identified significant enrichment of GO terms under category “Biological Process” in gills as well as head kidneys. Genes belonging to the category “generation of precursor metabolites and energy” (GO:0006091) were down-regulated genes in gills, whereas in head kidneys 7 GO terms were enriched for down-regulated genes (table S5). Important enriched down-regulated genes in head kidney tissue belonged to metabolic processes (GO:0008152) or to category “response to stimulus” (GO:0050896). Up-regulated genes show a more complex pattern, revealing a higher number of enriched GO terms (table S5). In gills, GO terms correspond to the development of cells and the organization of cellular components as well as to stimulus responses and signaling. Up-regulated genes in head kidney tissue also revealed enrichment of GO terms related to cell production and cell organization. Abundant transcripts involved in the pathways of interleukins (IL22RA, IL4R, IL6) and interferons (IRF4) indicated the presence of immune relevant cells. This also applies to chemokines (c-c chemokine receptor type 9, c–c chemokine 19 precursor) which play a central role in inflammatory responses [25]. The analysis of gene sets specific for a certain treatment showed no significant enrichment of GO terms. This is likely due to the small number of identified genes and thus reduced analytical power.

### Specific immune gene expression

**Figure 3:**
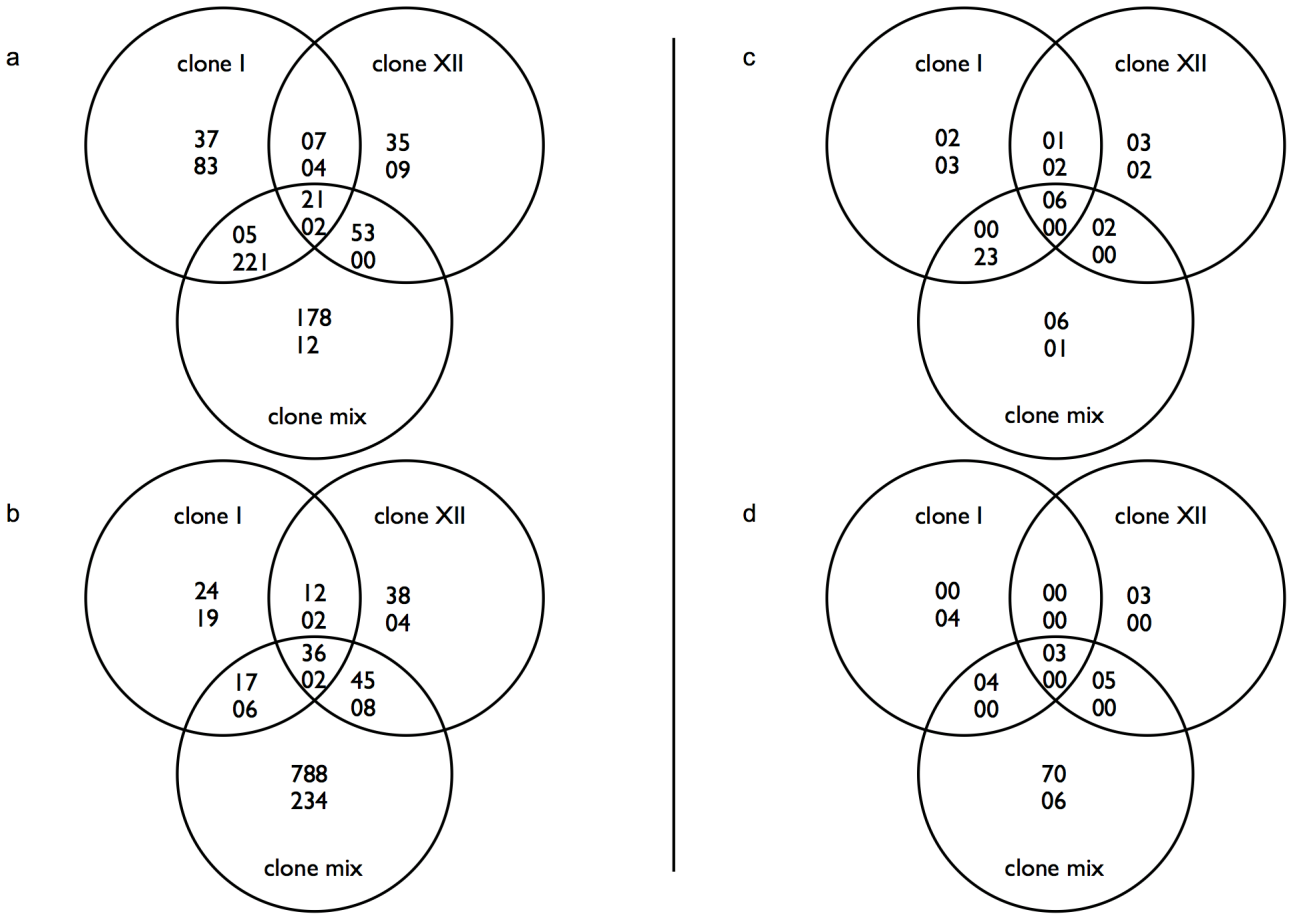
Venn diagrams for clone-wise distribution of differentially expressed genes in three-spined sticklebacks. Differential expression is defined as statistical significance in differences of gene expression between exposed and control fish. The first row of numbers shows up-regulated, the second row down-regulated genes. (a, b) Displayed are all genes with significantly different expression values in head kidney (a) and gills (b). (c, d) Venn diagrams show genes significantly different and associated to putative immune functions in head kidney (c) and gills (d).

**Figure 4:**
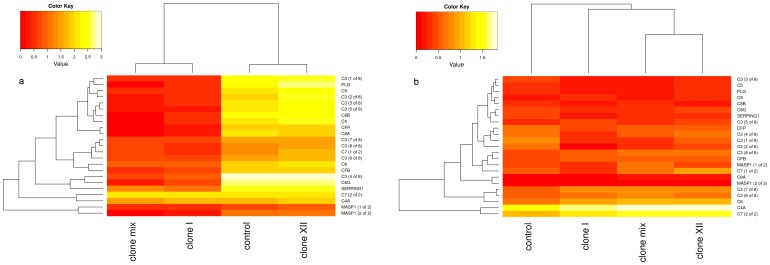
Heat map of genes attributed to the complement system. Shown are transformed FPKM values indicating high (bright) and low (dark) expression of genes for both mono-clonal, clone I (I) and clone XII (XII), and the clone mix (M) treatment as well as for the control in (a) head kidney tissue and (b) gills.

We investigated the expression of identifiable putative teleost immune genes (see methods) and observed differential expression in 139 out of 1067 genes (13%), with tissue specific expression revealing 55 immune genes in head kidney and 95 in gills (table S6 and S7; figure 3) that generally responded to the parasite infection. The majority of differentially expressed immune genes were down-regulated in head kidneys (31 out of 55) and up-regulated in gills (85 out of 95). 20 out of 139 immune genes (14%) were related to the complement system, including several isoforms of complement component 3 (C3, figure 4). The distribution of differentially expressed immune related genes allowed us to identify genes concertedly expressed between all treatments as a factor of *D. pseudospathaceum* infections in general. In head kidney samples, 6 genes were jointly up-regulated (THBS1, two isoforms of SOCS3, JUNB, IRF4, IL4RA). In gills only 3 shared up-regulated genes could be detected (THBS1, SOCS3, C4A). In up-regulated genes of head kidney samples, 2 genes respond to infection by *D. pseudospathaceum*-clone I (HYAL2, PRF1), 3 to clone XII (RGCC, CYP27B1, CCR9) and 6 to the clone mix treatment (ITGA5, SIX1, ATP1B3, MEF2C, MLF1, MYLPF). Uniquely down-regulated in head kidney were 3 genes in the clone I treatment (KYNU, C3, C7), 2 in clone XII (COL1A1, ZC3HAV1) and 1 in clone mix (VTN). Up-regulated immune related genes in gills could only be found in clone XII (ITGB1, C6, SLC3A2) and clone mix samples (70 genes, see table S7). In gills, treatment with clone I resulted in 4 down-regulated genes (GATA2, CASP3, F11R, EPHA2), whereas treatment with the clone mix caused down-regulation of 6 genes (PFDN1, EXOSC5, ATG12, ELMOD2, PRDX3, MHC II beta).

## Discussion

We provide evidence that innate immune gene responses in vertebrates can be specific for infecting parasite genotypes. The ability of innate immunity to detect bacterial, viral and fungal pathogens is ensured by several microbial sensors, including TLRs, NODs and the complement system, resulting in redundancy at the level of pathogen detection [26] [3]. Redundancy of the innate immune system is defined here as the interchangeability of gene products that lead to a certain immune response against an invading pathogen. Chemokines, signaling proteins that provide chemotaxis for leukocytes, show redundant responses to target cells, with each chemokine acting on several leukocyte populations [27]. This has been shown in mice were knock-out individuals depleted of transcriptional factors IRF3 and IRF7 were still able to mount a chemokine mediated antiviral immune response [28]. Similar findings have been shown in a response to parasitic helminths, were innate immunity can be induced via alternative pathways [29]. In our data, we could clearly distinguish between genes generally expressed in response to trematode infection and those only activated against specific clones. Such pattern can be explained by the activation of different receptor types which induce a parasite-genotype specific combination of innate immune responses upon first exposure of the host [3]. Differences between both single clone treatments imply redundant possibilities for the effector cascade of an innate immune response [26] [3]. The most parsimonious explanation of our results is that the genetic background of a specific parasite genotype influences the combination of triggered host receptor mechanisms, resulting in alternative combinations of response pathways [3]. *D. pseudospathaceum* actively penetrates the skin and migrates to the eye lens of the fish host, it thus needs to produce enzymes capable of disrupting host tissue and protecting itself from the host immune system [30]. We thus expect that genetically different parasite lineages of the same species are recognized by a different set of receptor molecules, inducing the observed differences in gene expression. In fact, the clone mix treatment shares the expression of genes present also in either of both single clone treatments (figure 3). Since both single clones are present in the clone mix, this further supports the proposed parasite clone specific host responses. This might even involve a regulatory strategy concerning fitness costs, in terms of tissue damaging potential of the immune response, which dictates the order of activated response pathways [26].

The elements active in innate immune responses against macroparasites such as the genus *Diplostomum* involve, among others, interleukins, macrophages and the complement system [22] [25] [31] [24]. However, while we have a substantial understanding of anti-microbial immune responses in vertebrates our knowledge on the immune responses to multicellular parasites has just expanded recently [31]. Notably, there is a large gap concerning the mechanisms for innate immune recognition of parasitic worms [32]. The present knowledge is partially reflected in our data where gene expression, which responds to *D. pseudospathaceum* infections in general, can be linked to macrophage activation, for example via the interleukin-associated gene products THBS1, IRF4 and IL4RA. We observe cytokine signaling (SOCS3, JUNB) and an activation of the complement system (C4A). The complement system with its several effector proteins can be activated through three pathways which all lead to the generation of factor C3 [25]. The activation of C3 in turn, is involved in enhanced phagocytosis, recruitment of immune cells, stimulation of B-cell proliferation, activation of inflammatory responses and the membrane attack complex [25], this has been demonstrated in fish as well [14].

Among immune genes differentially expressed in response to a specific trematode genotype infecting the fish we distinguished between head kidney and gill tissue. In both tissues we observed the unique expression of a number of genes (figure 3) including a putative influence of the complement system (figure 4). In head kidneys, we observed up-regulation of genes involved in cytolytic processes (PRF1) as well as cytokine activation (HYAL2) due to the clone I treatment, a pattern similar in the clone XII treatment (RGCC, CYP27B1) (www.uniprot.org). Contrary to the clone I response we also find CCR9 to be up-regulated in the clone XII treated samples. CCR9 is a chemokine receptor interacting with its ligand TECK, which has been shown to attract dendritic cells and macrophages [33]. Among down-regulated genes we observed reduced interferon activity (KYNU in clone I, ZC3HAV1 in clone XII) suggesting a macrophage-mediated migration of immune cells from head kidneys to the periphery [25]. On the other hand, infected fish displayed a down-regulation of complement genes in the clone I treatment (C3, C7) and a decreased amount of leukocyte activity in the clone XII response (COL1A1) (www.uniprot.org). Since we expected immune-relevant cells to migrate from the head kidney to the gills as a response to the parasite treatment, these observations suggest an increased complement-based response due to the clone I treatment compared to an increased leukocyte activity due to the clone XII treatment. This is supported by the genes differentially expressed in the gill tissue, where we observe an increase in leukocyte related genes (ITGB1, SLC3A2) due to the clone XII treatment and a decrease in the clone I treatment (F11R, EPHA2) (www.uniprot.org). However, we do see complement activity due to clone XII (C6) as well as putative leukocyte activation as a response to clone I (GATA2, CASP3) (www.uniprot.org), mediated as decreased expression of negative leukocyte regulation. Interestingly, changes in expression of C3 isoforms due to *D. pseudospathaceum* infections seem to be limited to the treatments involving clone I and/or clone mix (table S6 and S7). The activation of C4A in gills of all infected fish indicates an activated classical pathway of the complement system, which should also involve C3 (www.uniprot.org). Up-regulation of C6 in gills of fish infected with clone XII however suggests an increased amount of activity for the membrane attack complex (www.uniprot.org). Different isoforms of C3 have been detected in other fish species with antibody reactivities and binding affinities affected by the type of isoform expressed [14]. One possible interpretation would be that the complement system as central part in the response to *D. pseudospathaceum* infections is activated via different pathways depending on genotypic background of the parasite strain involved.

Although we did not observe a significantly different parasite load in sticklebacks between both single clone infection treatments, a diverse genetic background of invading parasite larvae (i.e. the clone mix) resulted in higher parasite load (figure 1). The infection success of *D. pseudospathaceum* can be increased in mixed infections compared in single clones, primarily due to the genetic diversity of exposure [34]. In addition, a lake environment might harbor a dense population of infected snails, making simultaneous infections with several parasite genotypes more likely than single genotype infections [34]. Coinfection with multiple pathogens is the rule rather than the exception and also applies to our study system where dense populations of intermediate hosts (snails) harbor a high parasite diversity [35]. It has been hypothesized that multiple infections decrease the effectiveness of resource allocation in defense mechanisms [36]. Theory predicts that the enhanced diversity of combined parasite genotypes increases the pressure on the host, thus causing a shift from optimal resource allocation to damage toleration, which could explain the higher infection rates in the clone mix treatment [36]. However, another study has shown effects of intra-specific competition in *D. pseudospathaceum*, resulting in reduced infection rates of clone mix treatments compared to single clone exposure [37]. In our case, the presence of outcrossed parasite genotypes in the clone mix treatment as opposed to the inbred single clones might explain the discrepancies to aforementioned study [38]. We were also not able to detect host-genotype influences on parasite-genotype infection rates. These genotype-by-genotype interactions have been found in three-spined sticklebacks [5] and, although our sampling was potent enough to detect treatment dependent differences, we cannot exclude the possibility that it might not be sufficient to detect the less pronounced effects of host-genotypes.

In conclusion we have demonstrated that a specific transcriptomic response in the fish host can be induced by a genetically defined parasite infection. With this study we present evidence that genetic variation in parasitic worms influences the mechanism with which the host immune system reacts to an immunological threat. The effect of different unicellular parasite genotypes on host gene expression has recently been shown in invertebrates [10]. Furthermore it has been widely recognized that immunological variation in host populations and the effect of different parasite species should be taken into account (reviewed in Maizels and Nussey 2013 [4]). In vertebrates, this has not yet been applied to intra-specific parasite genotype effects. To the best of our knowledge, there were so far no detailed data approximating the complex physiological processes that underlie within-species genetic affiliation and diversity in experimental infection of vertebrates by any macroparasite species. Host immune responses to parasitic worms are likely to induce multiple pathways instead of single molecular mechanisms [31], thus gene expression differences in our study likely result from genotype specific activation of redundant mechanisms. With this large scale gene expression data set we provide ample evidence for the importance of intra-specific variation in parasites on the level of host gene expression, expanding our knowledge about vertebrate immune systems.

## Materials and Methods

### Ethics statement

This study was performed according to the requirements of the German Protection of Animals Act (Tierschutzgesetz). All animal experiments were approved by the ‘Ministry of Energy, Agriculture, the Environment and Rural Areas’ of the state of Schleswig-Holstein, Germany (reference number: V 313-72241.123-34). Herring gulls were kept under permit number 1400-144/153-5.2.3, three-spined sticklebacks under 144-153-32, issued by Plön district administration, Germany. No further animal ethics committee approval was needed. Wild sticklebacks for breeding of parasite free F1 offspring were caught via minnow traps in the “Großer Plöner See” (Great Plön Lake) in Plön, Germany (54°08′53.8′′N, 10°25′49.3′′E). During the experiment fish were sacrificed by an incision into the brain followed by immediate decapitation, and every effort was made to minimize suffering. Eggs of herring gulls were collected on the island Ruhlebener Warder near Plön, Germany (54°08′30.8′′N, 10°26′14.4′′E) and kept under a heat lamp until hatching. Parasite eggs were collected from gull feces non-invasively. Gulls were returned to the wild 8 weeks after hatching. Parasite eggs for line establishment were collected from gull feces at the shore of the “Großer Plöner See” (Great Plön Lake) in Plön, Germany (54°9′21.16″N, 10°25′50.14″E). No specific permission was required for this location and activity. For a full description of methods used in obtaining parasite lineages and keeping herring gulls, see Rieger et al. (2013) “Genetic compatibilities, outcrossing rates and fitness consequences across life stages of the trematode *Diplostomum pseudospathaceum*.” Int J Parasitol 43∶485–491. All species used in this study are not endangered or protected.

### Clonal parasite lineages

Clonal lines of *D. pseudospathaceum* were established by collecting parasite eggs from gull feces at the shore of the “Großer Plöner See” (Great Plön Lake) in Plön, Germany (54°9′21.16″N, 10°25′50.14″E). Hatched miracidia, the first larval stage, were used to infect lab bred freshwater snails of the species *Lymnaea stagnalis*, the first intermediate host of *D. pseudospathaceum*. Snails were exposed to single miracidia under controlled conditions in 12-well-plates, ensuring mono-miracidial infections. Eight weeks post infection, snails were isolated and exposed to light for 3 h to check for production of cercaria, the second larval stage. From each of 4 snail groups, infected with miracidia from eggs originating from 4 different gull feces samples, the snail with the highest visible amount of cercaria production was chosen. Genotypes of cercariae emerging from snails were verified using the polymorphisms of 4 microsatellite loci (Diplo08, Diplo09, Diplo23 and Diplo29) [39]. To increase the amount of available cercariae and avoid snail effects we decided to multiply and propagate specific clonal parasite lineages by establishing each lineage in several host snails. Therefore, groups of lab bred sticklebacks (*Gasterosteus aculeatus*) were exposed to cercariae from individual mono-miracidial infected snails to serve as parasite reservoirs. Those fish were sacrificed and fed together with the infective parasite stages to uninfected European Herring Gulls (*Larus argentatus*). We collected gull feces of all infected gulls over two weeks and used single hatched, within clone inbred miracidia to infect in total 692 lab bred *L. stagnalis*, thus increasing the amount of snails available for a specific clonal parasite lineage.

### Sticklebacks & experimental infection

Three-spined stickleback (*G. aculeatus*) of 4 fish families, originating from the lake “Großer Plöner See”, were bred and raised under standardized conditions [20] at the Max Planck Institute for Evolutionary Biology (Plön, Germany). Hatchlings were kept for 7 months in 16 l aquaria. For experimental infection we randomly selected 12 fish per family and placed them individually in 1 l aquaria. Three individuals per family were either exposed to 100 cercaria-larvae of clone I, clone XII or a clone mix (several clonal lineages, containing inbred and outcrossed parasite genotypes) [38]. Three additional fish per family were treated the same way but not exposed to serve as control. All surviving infected snails were assessed for their respective parasite genotype prior to the experiment using microsatellites [38]. Only cercariae from snails where parasite genotype was unambiguous were used for the infection procedure.

Fish were killed 4 h after infection by an incision into the brain, followed by immediate decapitation and separation of gills. Body cavities were opened for instantaneous exposure of inner organs to preserving buffer (RNAlater, Qiagen). To reliably determine infection success an additional 3 fish from the same 4 fish families were exposed to the same parasite treatment under the exact same conditions as mentioned above. These fish were dissected after 4 weeks and eye lenses were checked for metacercaria-larvae of *D. pseudospathaceum*.

### RNA sequencing

The Macherey-Nagel NucleoSpin 96 RNA kit was used to extract RNA, following the standard protocol, and quality was checked with an Experion Automated Electrophoresis system (Bio-Rad). Samples were prepared for sequencing using the Illumina TruSeq RNA Sample Preparation Kit, following the standard protocol. Quality control and quantification of libraries was done using the LapChip GX (Caliper) and the HT DNA High Sensitivity Kit. For sequencing, indexed libraries were diluted to 2 nmol/l and pooled. To control quality of the sequencing run, a 1% PhiX control library (PhiX Control Kit v3, Illumina) was added to each lane. cDNA libraries were sequenced on the Illumina HiScan SQ platform for 2*101 cycles (paired-end), yielding a read length of 101 bp on both ends of the target sequence. We sequenced 32 indexed samples (as part of a 96 sample setup) distributed over one flowcell. Raw image data were transformed and de-multiplexed using CASAVA 1.8 software. Primary sequence data has been submitted to the NCBI short read archive (SRA) under the BioProject ID PRJNA253091.

### Statistics and data visualization

To estimate differences in parasite infection success, a two-way analysis of variance (ANOVA) was performed. Tukey’s test was used as posthoc test. Data were square-root transformed to meet assumptions of normality and homogeneity of variances. P-values <0.05 were considered significant. Visualization and modification of data sets, as well as statistical analysis of parasite infection data, was done with R (version 2.14.1, R Core Development Team, 2012) and custom made Python scripts, version 3.1.2.

### Quantification of gene expression

Sequence reads were aligned to the *G. aculeatus* reference genome, version 67 (www.ensembl.org), using TopHat [40] with standard parameters according to the manual. Quality of sequenced reads was checked via FastQC. For statistical analysis we used normalized gene expression values as fragments per kilobase of exon model per million mapped reads (FPKM) modified for paired-end data after RPKM [41]. The software Cufflinks (version 1.3.0) was used for calculation of differential expression with parasite infections as treatments and fish families as replicates within a treatment. Only known transcripts were used for the analysis. Upper quartile normalization was included to improve robustness of differential expression calls for less abundant transcripts. P-values indicate significance of estimated log2 fold changes with a correction for multiple testing (false discovery rate, FDR <0.05) [42].

### GO term enrichment analysis

We used GO terms to test for enrichment of functional categories between two distinct gene sets. Blast2GO was used for annotation of all reference-transcripts [43]. GO term enrichment was done using the integrated enrichment analysis function. We tested for differences between both mono-clonal treatments as well as for all genes differentially expressed compared to control versus all known stickleback genes. False discovery rate correction was applied after testing (FDR <0.05).

### Immune genes

In a second approach, we restricted the global transcriptome analysis a priori to all genes that are hypothesized to be involved in an immune response. To this end we extracted human genes tagged as “immune system process” (GO:0002376) from the ensembl database (www.ensembl.org). Only genes that could be unambiguously identified in the genome of *G. aculeatus* were kept for further analyses. This set of putative immune relevant genes was completed with a set of selected core immune genes [44] [45], resulting in a list containing 1067 putatively immune relevant genes (table S4).

## Supporting Information

Table S1 **Number of reads per sample, including treatment, fish family, organ type, flowcell lane number, total reads and mapped reads.** Two samples from gills and one sample from head kidney were of reduced quality and had to be removed from further analysis. This led to 4 samples in gills (clone XII, clone clone mix) and head kidney (clone I, clone XII, clone clone mix) as well as 3 samples in gills (clone I, control) and head kidney (control), resulting in 29 individual libraries.
(PDF)

Table S2 **Cufflinks output, list of differentially expressed genes in head kidney tissue of three-spined sticklebacks.** Differentially expressed is defined by comparison to uninfected controls. The term ”gene” is the name for a specific gene as taken from the G. aculeatus reference genome, ”locus” is the location on the genome, ”sample_1” is the control group, ”sample_2” is the infection treatment group, ”value_1” are FPKM values for ”sample_1”, ”value_2„ FPKM values for ”sample_2„, log2(fold change) displays the transformed fold change in ”sample_2” compared to ”sample_1”, the next three columns show the test statistic, p value and q value for each test, the last column shows whether the observed difference was significant (only significant differences shown).
(PDF)

Table S3 (PDF)

Table S4 **Putative immune system related genes in three-spined stickleback.** Gene names were obtained either via the ensembl biomart filter, querying the Homo sapiens transcriptome for genes belonging to ”immune system process” (GO:0002376), or via a published list containing immune genes identified in cod (Gadus morhua) and pipefish (Syngnathus typhle).
(PDF)

Table S5 **Enriched GO terms.** Shown are GO-terms (GO-ID, GO-term) of the group ”Biological Process” found to be overrepresented in a given test-set tested against the whole set of identified *G, aculeatus* genes. Given are number of genes per GO-term in test- (#Test) and reference-set (#Ref) with p-values and FDR corrections.
(PDF)

Table S6 **Differentially expressed immune-related genes in head kidney tissue of G. aculeatus.** Shown are differentially expressed genes and their corresponding treatment, including FPKM values for control (control_val) and treatment (treatment_val). The log2-fold change shows if there is up- or down-regulation of a given gene due to the parasite treatment. Only significant differences shown.
(PDF)

Table S7 **Differentially expressed immune-related genes in gill tissue of G. aculeatus.** Shown are differentially expressed genes and their corresponding treatment, including FPKM values for control (control_val) and treatment (treatment_val). The log2-fold change shows if there is up- or down-regulation of a given gene due to the parasite treatment. Only significant differences shown.
(PDF)
