# Supplementary material for: Specific Gene Expression Responses to Parasite Genotypes Reveal Redundancy of Innate Immunity in Vertebrates
Source: PLoS One. 2014 Sep 25;9(9):e108001. doi: 10.1371/journal.pone.0108001 (PMC4177871; doi:10.1371/journal.pone.0108001)
Supplement: Table S1 — Number of reads per sample, including treatment, fish family, organ type, flowcell lane number, total reads and mapped reads. Two samples from gills and one sample from head kidney were of reduced quality and had to be removed from further analysis. This led to 4 samples in gills (clone XII, clone clone mix) and head kidney (clone I, clone XII, clone clone mix) as well as 3 samples in gills (clone I, control) and head kidney (control), resulting in 29 individual libraries. (PDF) [file pone.0108001.s001.pdf]

**Supplementary table S.1** Number of reads per sample, including treatment, fish family, organ type, flowcell lane number, total reads and mapped reads. Two samples from gills and one sample from head kidney were of reduced quality and had to be removed from further analysis. This led to 4 samples in gills (clone XII, clone clone mix) and head kidney (clone I, clone XII, clone clone mix) as well as 3 samples in gills (clone I, control) and head kidney (control), resulting in 29 individual libraries.

| sample | fish_family | treatment | organ | lane_no | total_reads | aligned_reads | %_aligned |
|---|---|---|---|---|---|---|---|
| 1 | 10-10x15 | control | gills | L006 | 11193950 | 6374910 | 56,95 |
| 2 | 10-13x12 | control | gills | L001 | 6542490 | 3710021 | 56,71 |
| 3 | 10-2x1 | control | gills | L002 | 11781202 | 6625186 | 56,24 |
| 4 | 10-10x15 | clone mix | gills | L007 | 9034308 | 4817625 | 53,33 |
| 5 | 10-11x16 | clone mix | gills | L006 | 10700158 | 6461189 | 60,38 |
| 6 | 10-13x12 | clone mix | gills | L004 | 24710382 | 11990896 | 48,53 |
| 7 | 10-2x1 | clone mix | gills | L003 | 12123212 | 6914101 | 57,03 |
| 8 | 10-10x15 | clone I | gills | L004 | 11070914 | 5252831 | 47,45 |
| 9 | 10-11x16 | clone I | gills | L008 | 12455600 | 6233762 | 50,05 |
| 10 | 10-13x12 | clone I | gills | L004 | 13034054 | 6891659 | 52,87 |
| 11 | 10-10x15 | clone XII | gills | L008 | 14880032 | 6888039 | 46,29 |
| 12 | 10-11x16 | clone XII | gills | L007 | 9508384 | 4819680 | 50,69 |
| 13 | 10-13x12 | clone XII | gills | L006 | 9498616 | 5792914 | 60,99 |
| 14 | 10-2x1 | clone XII | gills | L005 | 9015786 | 4803562 | 53,28 |
| 15 | 10-10x15 | control | head_kidney | L005 | 10356510 | 5845850 | 56,45 |
| 16 | 10-11x16 | control | head_kidney | L008 | 16185886 | 8286884 | 51,20 |
| 17 | 10-2x1 | control | head_kidney | L004 | 19945222 | 10646606 | 53,38 |
| 18 | 10-10x15 | clone mix | head_kidney | L001 | 31556086 | 17647114 | 55,92 |
| 19 | 10-11x16 | clone mix | head_kidney | L008 | 20305784 | 9906271 | 48,79 |
| 20 | 10-13x12 | clone mix | head_kidney | L005 | 14366550 | 7398995 | 51,50 |
| 21 | 10-2x1 | clone mix | head_kidney | L006 | 18581584 | 11015042 | 59,28 |
| 22 | 10-10x15 | clone I | head_kidney | L005 | 14538858 | 7900850 | 54,34 |
| 23 | 10-11x16 | clone I | head_kidney | L007 | 14744676 | 8186321 | 55,52 |
| 24 | 10-13x12 | clone I | head_kidney | L004 | 20077788 | 11469317 | 57,12 |
| 25 | 10-2x1 | clone I | head_kidney | L003 | 28496486 | 19646137 | 68,94 |
| 26 | 10-10x15 | clone XII | head_kidney | L008 | 23881298 | 12756204 | 53,42 |
| 27 | 10-11x16 | clone XII | head_kidney | L008 | 42804942 | 21061942 | 49,20 |
| 28 | 10-13x12 | clone XII | head_kidney | L003 | 22337552 | 12241427 | 54,80 |
| 29 | 10-2x1 | clone XII | head_kidney | L001 | 22988590 | 13737901 | 59,76 |
