# Supplementary material for: Specific Gene Expression Responses to Parasite Genotypes Reveal Redundancy of Innate Immunity in Vertebrates
Source: PLoS One. 2014 Sep 25;9(9):e108001. doi: 10.1371/journal.pone.0108001 (PMC4177871; doi:10.1371/journal.pone.0108001)
Supplement: Table S5 — Enriched GO terms. Shown are GO-terms (GO-ID, GO-term) of the group ”Biological Process” found to be overrepresented in a given test-set tested against the whole set of identified G, aculeatus genes. Given are number of genes per GO-term in test- (#Test) and reference-set (#Ref) with p-values and FDR corrections. (PDF) [file pone.0108001.s005.pdf]

Supplementary table S.5 Enriched GO terms. Shown are GO-terms (GO-ID, GO-term) of the group „Biological Process“ found to be overrepresented in a given test-set tested against the whole set of identified *G, aculeatus* genes. Given are number of genes per GO-term in test- (#Test) and reference-set (#Ref) with p-values and FDR corrections.

test-set	GO-ID	GO-term	FDR	p-value	#Test	#Ref
total up-regulated in head kidney	GO:0032502	developmental process	2,46E-02	6,04E-04	101	4465
total up-regulated in head kidney	GO:0007275	multicellular organismal development	2,69E-02	7,96E-04	96	4216
total up-regulated in head kidney	GO:0032501	multicellular organismal process	2,69E-02	8,05E-04	96	4225
total up-regulated in head kidney	GO:0048869	cellular developmental process	3,98E-02	1,54E-03	61	2455
total up-regulated in head kidney	GO:0030154	cell differentiation	3,98E-02	1,54E-03	61	2455
total up-regulated in head kidney	GO:0009653	anatomical structure morphogenesis	3,98E-02	1,62E-03	58	2315
total up-regulated in head kidney	GO:0048856	anatomical structure development	3,98E-02	1,63E-03	58	2317
total down-regulated in head kidney	GO:0008152	metabolic process	4,53E-10	3,69E-12	182	7196
total down-regulated in head kidney	GO:0009056	catabolic process	4,36E-08	4,74E-10	70	1875
total down-regulated in head kidney	GO:0006629	lipid metabolic process	5,97E-05	1,14E-06	37	893
total down-regulated in head kidney	GO:0009605	response to external stimulus	7,57E-05	1,65E-06	46	1264
total down-regulated in head kidney	GO:0005975	carbohydrate metabolic process	3,82E-03	1,24E-04	25	627
total down-regulated in head kidney	GO:0019748	secondary metabolic process	9,22E-03	3,38E-04	8	94
total down-regulated in head kidney	GO:0006950	response to stress	9,22E-03	3,51E-04	58	2144
total up-regulated in gill	GO:0032502	developmental process	1,13E-18	6,12E-21	340	4226
total up-regulated in gill	GO:0032501	multicellular organismal process	4,70E-18	3,83E-20	324	3997
total up-regulated in gill	GO:0007275	multicellular organismal development	5,03E-18	5,46E-20	323	3989
total up-regulated in gill	GO:0050789	regulation of biological process	4,76E-13	1,16E-14	455	6800
total up-regulated in gill	GO:0065007	biological regulation	1,20E-12	3,26E-14	459	6916
total up-regulated in gill	GO:0009653	anatomical structure morphogenesis	2,36E-10	9,12E-12	184	2189
total up-regulated in gill	GO:0048856	anatomical structure development	2,36E-10	9,82E-12	184	2191
total up-regulated in gill	GO:0007010	cytoskeleton organization	2,36E-10	1,03E-11	79	652
total up-regulated in gill	GO:0050896	response to stimulus	3,56E-10	1,64E-11	352	5120
total up-regulated in gill	GO:0048869	cellular developmental process	4,10E-10	2,12E-11	191	2325
total up-regulated in gill	GO:0030154	cell differentiation	4,10E-10	2,12E-11	191	2325
total up-regulated in gill	GO:0016043	cellular component organization	4,87E-09	3,05E-10	265	3664
total up-regulated in gill	GO:0071840	cellular component organization or biogene	4,87E-09	3,05E-10	265	3664
total up-regulated in gill	GO:0007165	signal transduction	3,72E-08	2,59E-09	257	3604
total up-regulated in gill	GO:0009790	embryo development	3,72E-08	2,66E-09	107	1142
total up-regulated in gill	GO:0051716	cellular response to stimulus	3,72E-08	2,66E-09	257	3605
total up-regulated in gill	GO:0050794	regulation of cellular process	3,72E-08	2,73E-09	257	3606
total up-regulated in gill	GO:0009605	response to external stimulus	2,21E-07	1,80E-08	108	1202
total up-regulated in gill	GO:0023052	signaling	4,52E-06	4,79E-07	267	4006
total up-regulated in gill	GO:0009719	response to endogenous stimulus	1,78E-05	1,94E-06	67	704
total up-regulated in gill	GO:0006464	cellular protein modification process	1,95E-05	2,22E-06	144	1923
total up-regulated in gill	GO:0043412	macromolecule modification	1,95E-05	2,22E-06	144	1923
total up-regulated in gill	GO:0006996	organelle organization	3,56E-05	4,16E-06	133	1763
total up-regulated in gill	GO:0071841	cellular component organization or biogene	3,89E-05	4,76E-06	133	1768
total up-regulated in gill	GO:0071842	cellular component organization at cellular	3,89E-05	4,76E-06	133	1768
total up-regulated in gill	GO:0009987	cellular process	1,26E-04	1,58E-05	504	8771
total up-regulated in gill	GO:0008283	cell proliferation	5,11E-04	7,08E-05	88	1127
total up-regulated in gill	GO:0008219	cell death	6,20E-04	8,76E-05	101	1347
total up-regulated in gill	GO:0016265	death	6,58E-04	9,47E-05	101	1350
total up-regulated in gill	GO:0044267	cellular protein metabolic process	7,81E-03	1,23E-03	150	2319
total up-regulated in gill	GO:0006950	response to stress	1,75E-02	2,99E-03	133	2069
total up-regulated in gill	GO:0019538	protein metabolic process	2,61E-02	4,68E-03	185	3045
total down-regulated in gill	GO:0006091	generation of precursor metabolites and er	9,69E-03	6,83E-05	16	386
