# Supplementary material for: Specific Gene Expression Responses to Parasite Genotypes Reveal Redundancy of Innate Immunity in Vertebrates
Source: PLoS One. 2014 Sep 25;9(9):e108001. doi: 10.1371/journal.pone.0108001 (PMC4177871; doi:10.1371/journal.pone.0108001)
Supplement: Table S6 — Differentially expressed immune-related genes in head kidney tissue of G. aculeatus. Shown are differentially expressed genes and their corresponding treatment, including FPKM values for control (control_val) and treatment (treatment_val). The log2-fold change shows if there is up- or down-regulation of a given gene due to the parasite treatment. Only significant differences shown. (PDF) [file pone.0108001.s006.pdf]

Supplementary table S.6 Differentially expressed immune-related genes in head kidney tissue of *G. aculeatus*. Shown are differentially expressed genes and their corresponding treatment, including FPKM values for control (control_val) and treatment (treatment_val). The log2-fold change shows if there is up- or down-regulation of a given gene due to the parasite treatment. Only significant differences shown.

gene	treatment	control_val	treatment_val	log2(fold_change)
APOB (5 of 5)	clone I	397,857	2,52703	-7,29866
PGLYRP2 (2 of 2)	clone I	302,534	2,23121	-7,08313
APOB (1 of 5)	clone I	142,41	1,13425	-6,97217
C8G	clone I	564,245	4,5427	-6,95663
C3 (1 of 8)	clone I	253,781	2,14946	-6,88346
C8B	clone I	189,341	1,62499	-6,86441
C3 (4 of 8)	clone I	862,33	8,5632	-6,65395
VTN (2 of 2)	clone I	52,3546	0,527891	-6,63193
C3 (5 of 8)	clone I	131,06	1,43388	-6,51416
C3 (3 of 8)	clone I	159,784	1,87542	-6,41276
PLG	clone I	216,823	2,54515	-6,41262
CFP	clone I	95,5085	1,17732	-6,34205
C8A	clone I	107,029	1,39908	-6,25739
SUSD2	clone I	9,21498	0,148896	-5,9516
C9	clone I	141,829	2,39006	-5,89096
ENSGACG00000003030	clone I	50,9171	0,928614	-5,77693
G6PD (2 of 2)	clone I	13,1035	0,242406	-5,75639
ENSGACG00000014811	clone I	107,827	2,36189	-5,51263
C3 (2 of 8)	clone I	100,881	2,30492	-5,45179
ENSGACG00000014852	clone I	56,6342	2,57792	-4,45739
PVRL1 (2 of 2)	clone I	11,0099	0,514717	-4,41888
CFB	clone I	76,4034	3,73169	-4,35573
SERPING1	clone I	265,943	13,3341	-4,31792
C6	clone I	98,9399	6,35633	-3,96029
ENPP2 (2 of 2)	clone I	39,8483	2,61654	-3,92879
KYNU	clone I	9,8258	0,762041	-3,68863
C7 (1 of 2)	clone I	20,5733	1,85433	-3,47181
ADSSL1	clone I	22,0206	2,48666	-3,14657
C3 (8 of 8)	clone I	24,4008	2,94383	-3,05116
MYLPF (2 of 2)	clone I	141,621	20,0123	-2,82307
C3 (7 of 8)	clone I	25,5542	3,86762	-2,72404
ADAMTS13	clone I	6,34378	1,61993	-1,96941
HYAL2 (1 of 2)	clone I	14,5824	32,8338	1,17095
IRF4 (2 of 2)	clone I	24,12	64,276	1,41405
JUNB (1 of 2)	clone I	14,9186	76,5929	2,3601
ENSGACG00000016298	clone I	21,2148	136,593	2,68674
PRF1 (2 of 5)	clone I	2,84543	19,7145	2,79254
THBS1 (2 of 2)	clone I	5,34663	41,8024	2,96688
SOCS3 (2 of 2)	clone I	6,09284	69,9715	3,52158
JUNB (2 of 2)	clone I	4,48677	68,0492	3,92283
SOCS3 (1 of 2)	clone I	8,54547	277,545	5,02142
VTN (1 of 2)	clone mix	290,729	0,291691	-9,96102
C8B	clone mix	189,341	0,246136	-9,58731
PGLYRP2 (2 of 2)	clone mix	302,534	0,491203	-9,26656
PLG	clone mix	216,823	0,38897	-9,12264
APOB (5 of 5)	clone mix	397,857	0,736573	-9,07721
C8G	clone mix	564,245	1,19664	-8,88119
ENSGACG00000014811	clone mix	107,827	0,302411	-8,47799
APOB (1 of 5)	clone mix	142,41	0,401887	-8,46904
C8A	clone mix	107,029	0,371097	-8,172
C3 (4 of 8)	clone mix	862,33	3,43326	-7,97252
C3 (3 of 8)	clone mix	159,784	0,846626	-7,56018
CFP	clone mix	95,5085	0,510596	-7,5473
C3 (5 of 8)	clone mix	131,06	0,811535	-7,33536
C3 (1 of 8)	clone mix	253,781	1,58069	-7,32688
C9	clone mix	141,829	1,58198	-6,48627
SUSD2	clone mix	9,21498	0,107208	-6,4255
C3 (2 of 8)	clone mix	100,881	1,47385	-6,09692
ENSGACG00000003030	clone mix	50,9171	1,36813	-5,21788
CFB	clone mix	76,4034	2,06039	-5,21265
G6PD (2 of 2)	clone mix	13,1035	0,459733	-4,83302
ENSGACG00000014852	clone mix	56,6342	2,01001	-4,8164
VTN (2 of 2)	clone mix	52,3546	2,73782	-4,25722
SERPING1	clone mix	265,943	17,0305	-3,96492
C6	clone mix	98,9399	6,54284	-3,91856
ENPP2 (2 of 2)	clone mix	39,8483	3,13234	-3,66921
C3 (8 of 8)	clone mix	24,4008	3,10237	-2,97549
IRF4 (2 of 2)	clone mix	24,12	83,3801	1,78947
ITGA5 (1 of 2)	clone mix	3,89684	19,3759	2,31389
THBS1 (2 of 2)	clone mix	5,34663	28,5636	2,41748
ENSGACG00000016298	clone mix	21,2148	124,818	2,55669
SOCS3 (2 of 2)	clone mix	6,09284	47,1337	2,95157
SIX1	clone mix	2,98137	31,7399	3,41225
JUNB (2 of 2)	clone mix	4,48677	47,7688	3,41232
PIP5K1C (2 of 2)	clone mix	0,832627	10,2316	3,61921
ATP1B3	clone mix	3,11375	55,4923	4,15556
CRIP2 (2 of 2)	clone mix	2,78183	50,6523	4,18652
ADSSL1	clone mix	22,0206	439,238	4,31808
MEF2C (2 of 2)	clone mix	1,28259	30,988	4,59457
SOCS3 (1 of 2)	clone mix	8,54547	230,874	4,7558
MLF1	clone mix	0,781018	21,7563	4,79993
MYLPF (1 of 2)	clone mix	1,35951	2943,02	11,08
PVRL1 (2 of 2)	clone XII	11,0099	0,538633	-4,35336
MYLPF (2 of 2)	clone XII	141,621	16,6078	-3,0921
COL1A1 (2 of 2)	clone XII	57,8938	25,687	-1,17237
ZC3HAV1	clone XII	35,2857	21,6113	-0,707299
ADAMTS13	clone XII	6,34378	11,0225	0,797039
IRF4 (2 of 2)	clone XII	24,12	115,193	2,25575
RGCC	clone XII	28,2047	157,304	2,47955
ENSGACG00000016298	clone XII	21,2148	135,673	2,67699
CYP27B1	clone XII	7,50464	51,4143	2,77632
THBS1 (2 of 2)	clone XII	5,34663	37,0327	2,7921
JUNB (1 of 2)	clone XII	14,9186	106,155	2,83099
CCR9 (1 of 2)	clone XII	2,60228	31,9553	3,61821
CRIP2 (2 of 2)	clone XII	2,78183	39,5776	3,83058
PIP5K1C (2 of 2)	clone XII	0,832627	12,8216	3,94477
JUNB (2 of 2)	clone XII	4,48677	102,8	4,51802
SOCS3 (2 of 2)	clone XII	6,09284	140,181	4,52403
ENSGACG00000014852	clone XII	56,6342	2208,16	5,28503
ENSGACG00000003030	clone XII	50,9171	2822,85	5,79286
SOCS3 (1 of 2)	clone XII	8,54547	632,269	6,20923
