# Supplementary material for: Specific Gene Expression Responses to Parasite Genotypes Reveal Redundancy of Innate Immunity in Vertebrates
Source: PLoS One. 2014 Sep 25;9(9):e108001. doi: 10.1371/journal.pone.0108001 (PMC4177871; doi:10.1371/journal.pone.0108001)
Supplement: Table S7 — Differentially expressed immune-related genes in gill tissue of G. aculeatus. Shown are differentially expressed genes and their corresponding treatment, including FPKM values for control (control_val) and treatment (treatment_val). The log2-fold change shows if there is up- or down-regulation of a given gene due to the parasite treatment. Only significant differences shown. (PDF) [file pone.0108001.s007.pdf]

Supplementary table S.7 Differentially expressed immune-related genes in gill tissue of *G. aculeatus*. Shown are differentially expressed genes and their corresponding treatment, including FPKM values for control (control_val) and treatment (treatment_val). The log2-fold change shows if there is up- or down-regulation of a given gene due to the parasite treatment. Only significant differences shown.

gene	treatment	control_val	treatment_val	log2(fold_change)
GATA2 (1 of 2)	clone I	110,657	64,4838	-0,779088
CASP3 (4 of 4)	clone I	450,008	269,981	-0,73709
F11R	clone I	282,828	187,116	-0,595993
EPHA2 (1 of 2)	clone I	24,5537	18,464	-0,411225
COL1A1 (2 of 2)	clone I	120,383	195,736	0,701278
C4A	clone I	26,6354	44,8943	0,753185
PIK3R1 (1 of 2)	clone I	32,8272	76,9047	1,22818
NFKBIA (1 of 2)	clone I	101,293	256,941	1,3429
CCR9 (1 of 2)	clone I	15,7446	43,8927	1,47913
THBS1 (2 of 2)	clone I	2,65074	14,2046	2,42189
SOCS3 (1 of 2)	clone I	25,2321	151,034	2,58154
PFDN1	clone mix	113,611	33,5984	-1,75764
EXOSC5	clone mix	89,0188	28,0065	-1,66835
ATG12	clone mix	50,5042	16,175	-1,64264
ELMOD2	clone mix	99,0101	47,3581	-1,06397
ENSGACG00000000336	clone mix	646,991	391,739	-0,723854
PRDX3	clone mix	128,492	87,3783	-0,556329
DCTN1 (2 of 2)	clone mix	21,9148	35,4999	0,695912
ZC3HAV1	clone mix	57,9356	102,987	0,829933
CHUK (1 of 2)	clone mix	15,6785	28,4516	0,859719
PKHD1L1	clone mix	3,33013	6,26935	0,912738
PRKDC	clone mix	5,5653	11,5522	1,05364
CXCL12 (2 of 2)	clone mix	180,438	381,163	1,0789
VAV3 (2 of 2)	clone mix	7,66529	16,7189	1,12506
MYO9B (1 of 2)	clone mix	11,7707	25,8328	1,134
KLC1	clone mix	10,2467	22,7065	1,14795
MINK1	clone mix	12,5942	27,9295	1,14903
FGFR2	clone mix	38,7514	87,4171	1,17367
TRPM4 (2 of 2)	clone mix	16,1638	36,749	1,18494
LYST	clone mix	2,52577	5,75517	1,18814
ROCK1	clone mix	19,4466	44,4478	1,1926
JAK1	clone mix	42,4953	97,2966	1,19508
IL6ST	clone mix	19,5226	44,7802	1,19771
CHD7	clone mix	6,55623	15,0938	1,20302
PIK3CB	clone mix	18,7046	43,1989	1,2076
TGFBR3	clone mix	15,8447	36,6134	1,20837
MALT1	clone mix	5,48002	12,6746	1,20969
AP3D1	clone mix	10,9076	25,3267	1,21533
TCF3 (1 of 2)	clone mix	12,5922	29,3723	1,22193
NCOA6	clone mix	6,04059	14,0976	1,22269
NLRC5	clone mix	7,72544	18,1536	1,23256
MAPK14 (1 of 2)	clone mix	36,8271	86,8727	1,23813
RICTOR	clone mix	7,93354	18,8267	1,24674
C3 (6 of 8)	clone mix	2,23888	5,32882	1,25104
ACIN1	clone mix	21,5025	51,4822	1,25957
PRKCA (2 of 2)	clone mix	7,20845	17,3013	1,26312
PSME4 (1 of 2)	clone mix	11,7679	28,8033	1,29137
C3 (8 of 8)	clone mix	1,66511	4,16296	1,32199
PIK3CG	clone mix	7,73283	19,6629	1,34641
C4A	clone mix	26,6354	68,1728	1,35585
COL1A1 (2 of 2)	clone mix	120,383	312,219	1,37493
PDE1B	clone mix	11,871	30,8535	1,37799
MAP4K2	clone mix	10,3164	27,0149	1,38881
ENSGACG00000001919	clone mix	118,854	316,304	1,41213
MLL	clone mix	3,41602	9,25116	1,43732
SLC11A2 (1 of 2)	clone mix	8,2926	22,76	1,45661
TNRC6C (2 of 2)	clone mix	2,86844	7,96076	1,47264
EP300 (1 of 2)	clone mix	3,93953	10,9489	1,47468
EGR1	clone mix	17,677	49,5211	1,48617
ITCH (2 of 2)	clone mix	4,34254	12,3402	1,50676
CYSLTR2	clone mix	8,98739	25,6988	1,51573
JAG2	clone mix	3,96985	11,4035	1,52232
TGFB1	clone mix	19,2995	56,4909	1,54946
SWAP70 (1 of 2)	clone mix	25,7638	75,9629	1,55995
C7 (2 of 2)	clone mix	11,3621	34,368	1,59683
SKIL	clone mix	5,59669	17,1667	1,61697
PTPN1	clone mix	12,4365	38,4085	1,62685
PREX1	clone mix	7,1903	22,6468	1,65518
MECOM	clone mix	2,79049	8,89955	1,67321
KLF6 (1 of 2)	clone mix	38,6488	127,676	1,72399
THRA (1 of 2)	clone mix	6,17339	20,5508	1,73506
IGF1R (2 of 2)	clone mix	3,76793	12,7508	1,75874
SLC16A1 (1 of 2)	clone mix	10,4441	35,4457	1,76292
PGLYRP2 (1 of 2)	clone mix	12,1395	43,2742	1,83379
SFRP2	clone mix	14,8646	53,0794	1,83627
MKNK2	clone mix	61,4839	221,94	1,85189
COL1A2	clone mix	67,0945	252,776	1,9136
COLEC12 (2 of 2)	clone mix	6,56252	25,2012	1,94117
TINAGL1	clone mix	18,6589	74,4725	1,99684
FN1 (2 of 2)	clone mix	5,47172	21,9932	2,00699
DAB2	clone mix	5,07582	20,4465	2,01014
CCR9 (1 of 2)	clone mix	15,7446	65,1434	2,04877
HDAC4 (1 of 2)	clone mix	3,84143	15,899	2,04922
EDN2	clone mix	9,09681	37,761	2,05346
SRF (2 of 2)	clone mix	2,85015	11,9942	2,07322
PIK3R1 (1 of 2)	clone mix	32,8272	138,861	2,08068
FN1 (1 of 2)	clone mix	11,812	50,042	2,08289
JUNB (2 of 2)	clone mix	15,1382	66,1757	2,1281
ITGA1	clone mix	8,11723	35,7251	2,13788
NFKBIA (1 of 2)	clone mix	101,293	453,214	2,16166
ITGA5 (1 of 2)	clone mix	1,8522	8,67928	2,22833
PODXL	clone mix	6,93071	32,6175	2,23457
KIF3C (1 of 2)	clone mix	2,13274	10,4545	2,29334
JUNB (1 of 2)	clone mix	22,0675	136,157	2,62527
DUSP6	clone mix	8,66461	56,9579	2,71669
SOCS3 (2 of 2)	clone mix	8,34668	65,733	2,97734
ADAM8 (1 of 2)	clone mix	1,99632	15,7632	2,98115
THBS1 (2 of 2)	clone mix	2,65074	34,9675	3,72154
SOCS3 (1 of 2)	clone mix	25,2321	334,289	3,72776
ITGB1 (1 of 2)	clone XII	35,9397	55,5941	0,629352
C4A	clone XII	26,6354	59,4999	1,15954
DAB2	clone XII	5,07582	14,08	1,47193
C6	clone XII	3,91636	10,9939	1,48911
SLC3A2 (2 of 2)	clone XII	57,6051	203,394	1,82001
JUNB (2 of 2)	clone XII	15,1382	63,0149	2,0575
SOCS3 (2 of 2)	clone XII	8,34668	47,5986	2,51164
ITGA5 (1 of 2)	clone XII	1,8522	12,0105	2,69698
ADAM8 (1 of 2)	clone XII	1,99632	14,0493	2,81508
SOCS3 (1 of 2)	clone XII	25,2321	250,36	3,31067
THBS1 (2 of 2)	clone XII	2,65074	34,5915	3,70595
