# Supplementary material for: Specific Gene Expression Responses to Parasite Genotypes Reveal Redundancy of Innate Immunity in Vertebrates
Source: PLoS One. 2014 Sep 25;9(9):e108001. doi: 10.1371/journal.pone.0108001 (PMC4177871; doi:10.1371/journal.pone.0108001)
Supplement: Table S2 — Cufflinks output, list of differentially expressed genes in head kidney tissue of three-spined sticklebacks. Differentially expressed is defined by comparison to uninfected controls. The term ”gene” is the name for a specific gene as taken from the G. aculeatus reference genome, ”locus” is the location on the genome, ”sample_1” is the control group, ”sample_2” is the infection treatment group, ”value_1” are FPKM values for ”sample_1”, ”value_2„ FPKM values for ”sample_2„, log2(fold change) displays the transformed fold change in ”sample_2” compared to ”sample_1”, the next three columns show the test statistic, p value and q value for each test, the last column shows whether the observed difference was significant (only significant differences shown). (PDF) [file pone.0108001.s002.pdf]

Supplementary table S.2 Cufflinks output, list of differentially expressed genes in head kidney tissue of three-spined sticklebacks. Differentially expressed is defined by comparison to uninfected controls. The term „gene" is the name for a specific gene as taken from the *G. aculeatus* reference genome, „locus" is the location on the genome, „sample_1" is the control group, „sample_2" is the infection treatment group, „value_1" are FPKM values for „sample_1", „value_2" FPKM values for „sample_2", log2(fold change) displays the transformed fold change in „sample_2" compared to „sample_1", the next three columns show the test statistic, p value and q value for each test, the last column shows whether the observed difference was significant (only significant differences shown).

gene	locus	sample_1	sample_2	value_1	value_2	log2(fold_change)	test_stat	p_value	q_value	significant
SMTNL2	groupI:1260570-1274267	control	clone XII	2,23133	26,5148	3,57082	-3,11312	0,00185121	0,0252328	yes
SCARF1	groupI:7557007-7561871	control	clone XII	1,35193	15,7684	3,54394	-3,38856	0,000702598	0,0115278	yes
SLC13A2 (1 of 2)	groupI:7578221-7584388	control	clone XII	1,19994	25,2882	4,39743	-4,48148	7,41E-06	0,000257352	yes
BLVRB	groupI:16058123-16061240	control	clone XII	163,801	78,309	-1,06469	2,98868	0,00280186	0,0350824	yes
ENSGACG00000014752	groupI:22723989-22724814	control	clone XII	73,7738	6,65285	-3,47107	3,23053	0,00123559	0,0182374	yes
ACADL	groupI:22725531-22729041	control	clone XII	8,51763	31,892	1,90467	-6,06688	1,30E-09	1,49E-07	yes
SLC1A5	groupI:9146858-9153860	control	clone XII	9,29285	105,952	3,51114	-3,97155	7,14E-05	0,00173578	yes
ENSGACG00000012245	groupI:15150060-15151052	control	clone XII	3,6098	32,9034	3,18824	-2,86121	0,00422023	0,0484099	yes
ADPRHL1	groupII:7768909-7772963	control	clone XII	0,339998	13,3579	5,29602	-3,69892	0,000216518	0,00437762	yes
RRAD	groupII:8210016-8212357	control	clone XII	2,13092	28,0373	3,7178	-3,26541	0,00109305	0,0165155	yes
ENSGACG00000016298	groupII:14551247-14569051	control	clone XII	21,2148	135,673	2,67699	-4,32536	1,52E-05	0,000473464	yes
ALPK3 (1 of 2)	groupII:15268483-15277781	control	clone XII	0,155975	5,1052	5,03258	-3,12774	0,00176154	0,0242358	yes
ENSGACG00000016813	groupII:18474082-18485088	control	clone XII	3,60304	16,2603	2,17407	-4,51455	6,35E-06	0,000225556	yes
CSRP3	groupII:19781207-19783294	control	clone XII	0,554312	55,6374	6,64921	-3,30396	0,000953306	0,014776	yes
ENSGACG00000012663	groupIII:205052-212076	control	clone XII	0,272941	4,93179	4,17545	-3,02619	0,00247655	0,0318205	yes
ENSGACG00000014960	groupIII:6336030-6349511	control	clone XII	0,194248	44,1495	7,82835	-4,27933	1,87E-05	0,000565075	yes
ENSGACG00000015175	groupIII:7098657-7106144	control	clone XII	5,7217	35,6068	2,63764	-2,89985	0,00373337	0,0440022	yes
PIP5K1C (2 of 2)	groupIII:7710662-7721459	control	clone XII	0,832627	12,8216	3,94477	-3,63847	0,000274262	0,00532287	yes
FAM102B (2 of 2)	groupIII:7039318-7046081	control	clone XII	0,911866	9,14046	3,32537	-2,93647	0,00331975	0,0401188	yes
MYLK4 (2 of 2)	groupIII:10620516-10623791	control	clone XII	1,90815	25,9336	3,76457	-3,29732	0,000976123	0,015064	yes
OBSCN	groupIII:14055165-14092796	control	clone XII	0,908085	7,90793	3,1224	-3,65116	0,000261055	0,00511107	yes
FOSL1	groupIV:5471462-5474199	control	clone XII	0,765935	69,9279	6,5125	-4,90227	9,47E-07	4,47E-05	yes
ANXA6	groupIV:13375788-13385299	control	clone XII	1,40423	16,8382	3,58389	-3,18398	0,00145263	0,0207619	yes
ACSL4	groupIV:15052252-15058258	control	clone XII	14,7063	41,9467	1,51213	-3,13875	0,00169672	0,0235202	yes
LMOD2 (1 of 2)	groupIV:17997325-18001058	control	clone XII	0,58957	22,5083	5,25465	-2,96852	0,00299241	0,0369881	yes
PFKFB3	groupIV:22147683-22153447	control	clone XII	3,48231	29,3261	3,07407	-3,4272	0,000609832	0,0102663	yes
CCRN4L (1 of 2)	groupIV:27563357-27572709	control	clone XII	5,92166	109,628	4,21047	-3,12173	0,00179793	0,0246392	yes
ENSGACG00000019744	groupIV:29692169-29694681	control	clone XII	1,68518	223,509	7,05129	-3,75056	0,00017644	0,0036912	yes
SEMA3A (2 of 2)	groupIV:29946460-29961696	control	clone XII	2,02944	29,2115	3,84738	-3,83644	0,000124828	0,0027673	yes
SLC25A43	groupIV:5559504-5568467	control	clone XII	52,8035	212,185	2,00662	-6,09712	1,08E-09	1,26E-07	yes
FHL1 (1 of 2)	groupIV:6211431-6220387	control	clone XII	10,6395	57,383	2,4312	-2,84998	0,00437216	0,0497423	yes
ENSGACG00000018662	groupIV:16111937-16112913	control	clone XII	2,14062	87,7155	5,35673	-4,59853	4,25E-06	0,000160752	yes
JUNB (2 of 2)	groupIX:15395448-15396453	control	clone XII	4,48677	102,8	4,51802	-4,28228	1,85E-05	0,000558641	yes
SLC8A1 (2 of 2)	groupIX:19919460-19931018	control	clone XII	0,248414	32,5848	7,03531	-3,06588	0,00217029	0,0286471	yes
SORBS2	groupIX:5510578-5528882	control	clone XII	0,981881	16,2959	4,05282	-3,23803	0,00120357	0,0178536	yes
SMOX	groupIX:8508224-8518163	control	clone XII	10,698	600,384	5,81048	-4,69952	2,61E-06	0,00010597	yes
MYOZ2 (1 of 2)	groupIX:8678716-8682685	control	clone XII	1,47506	47,9981	5,02413	-3,91583	9,01E-05	0,00210714	yes
DDIT4 (1 of 2)	groupV:834468-835775	control	clone XII	21,1832	742,085	5,13059	-4,64179	3,45E-06	0,000134727	yes
MYBPC2 (1 of 2)	groupV:10018871-10040862	control	clone XII	8,76993	0,757086	-3,53404	3,79472	0,00014781	0,00318601	yes
UROS	groupV:1387477-1389630	control	clone XII	51,0921	22,2045	-1,20225	3,0864	0,00202598	0,0271208	yes
SYNPO2L (2 of 2)	groupV:2058537-2067911	control	clone XII	0,190437	14,5262	6,2532	-3,81684	0,000135171	0,00295699	yes
SOCS3 (2 of 2)	groupV:2198432-2199203	control	clone XII	6,09284	140,181	4,52403	-3,96145	7,45E-05	0,00179809	yes
COL1A1 (2 of 2)	groupV:3433394-3454716	control	clone XII	57,8938	25,687	-1,17237	3,97503	7,04E-05	0,00171469	yes
COX6B1 (1 of 2)	groupV:10666447-10667261	control	clone XII	13,5583	216,876	3,99961	-3,02422	0,00249279	0,0319907	yes
ACE	groupV:11674618-11688534	control	clone XII	5,03862	11,7374	1,22001	-3,01643	0,0025577	0,032646	yes
PROM2	groupVI:8443462-8452120	control	clone XII	1,18289	10,2024	3,10851	-3,01901	0,00253599	0,0324275	yes
LDB3	groupVI:15326453-15352359	control	clone XII	0,855691	12,2005	3,8337	-3,15268	0,00161777	0,0226343	yes
BAG3	groupVI:2938723-2942498	control	clone XII	5,35787	79,0231	3,88254	-2,99119	0,00277896	0,0348567	yes
AVPI1	groupVI:9052763-9054969	control	clone XII	7,71055	48,7292	2,65988	-2,89982	0,00373378	0,0440063	yes
ENSGACG00000010126	groupVI:12376810-12424549	control	clone XII	0,044435	4,94232	6,79735	-3,82171	0,000132529	0,00290846	yes
ACTN2	groupVI:12428216-12443132	control	clone XII	0,686077	48,5092	6,14374	-3,71788	0,000200904	0,00411318	yes
HSPB1	groupVII:14587962-14593757	control	clone XII	13,0002	142,385	3,4532	-3,69091	0,000223452	0,00449394	yes
PVRL1 (2 of 2)	groupVII:21614700-21623583	control	clone XII	11,0099	0,538633	-4,35336	2,89607	0,00377867	0,0444195	yes
ENSGACG00000020727	groupVII:23258674-23261239	control	clone XII	2,31207	45,9853	4,31392	-4,50459	6,65E-06	0,000234774	yes
LRCH2	groupVII:22687320-22700908	control	clone XII	4,69888	9,75919	1,05445	-2,85663	0,00428167	0,0489486	yes
IRF4 (2 of 2)	groupVIII:3510443-3515871	control	clone XII	24,12	115,193	2,25575	-3,4215	0,000622756	0,0104458	yes
ENSGACG00000010050	groupVIII:12782195-12784724	control	clone XII	6,09156	75,6528	3,63451	-6,59751	4,18E-11	7,08E-09	yes
GADD45B (1 of 2)	groupVIII:17587366-17588960	control	clone XII	12,5456	57,864	2,20549	-2,88931	0,00386086	0,0451694	yes
IGFBP1 (1 of 2)	groupVIII:3323546-3325070	control	clone XII	6,64213	389,223	5,87281	-3,50006	0,00046516	0,00823314	yes
MYLK4 (1 of 2)	groupVIII:3765987-3770589	control	clone XII	0,69573	43,2216	5,95708	-3,35876	0,000782943	0,0125899	yes
ENSGACG00000005760	groupVIII:5120336-5122462	control	clone XII	12,2208	59,1046	2,27393	-3,918	8,93E-05	0,00209134	yes
PRAM1	groupVIII:9640470-9647257	control	clone XII	69,785	11,736	-2,57198	3,22687	0,00125151	0,0184265	yes
ELTD1	groupVIII:12041724-12054205	control	clone XII	6,45516	40,5094	2,64973	-3,93655	8,27E-05	0,00196096	yes
GZMM (3 of 5)	groupVIII:16529393-16532680	control	clone XII	406,144	56,4309	-2,84743	8,82215	0	0	yes
ENSGACG00000008242	groupX:12654371-12658742	control	clone XII	15,8101	1,72233	-3,19841	3,16156	0,00156927	0,0220915	yes
JUNB (1 of 2)	groupXI:3309436-3310423	control	clone XII	14,9186	106,155	2,83099	-2,86058	0,00422861	0,0484831	yes
ENSGACG00000007447	groupXI:4320058-4324681	control	clone XII	3,75165	9,22147	1,29747	-3,13166	0,00173823	0,0239772	yes
ENSGACG00000008064	groupXI:5057905-5058802	control	clone XII	1,52053	26,8809	4,14393	-3,49378	0,000476233	0,00839255	yes
TCAP	groupXI:6193595-6194777	control	clone XII	2,24873	51,4578	4,51621	-3,95242	7,74E-05	0,00185575	yes
MYLPF (2 of 2)	groupXI:13368252-13371517	control	clone XII	141,621	16,6078	-3,0921	2,9606	0,0030704	0,0377321	yes
ENSGACG00000008053	groupXI:5019934-5020840	control	clone XII	7,88532	75,7903	3,26477	-3,42507	0,000614647	0,0103346	yes
ENSGACG00000008055	groupXI:5020849-5021746	control	clone XII	8,90505	66,6108	2,90306	-3,18056	0,0014699	0,0209609	yes
SOCS3 (1 of 2)	groupXI:9066120-9067557	control	clone XII	8,54547	632,269	6,20923	-5,20341	1,96E-07	1,15E-05	yes
TNNC2 (1 of 2)	groupXII:3692239-3694594	control	clone XII	38,9134	3,60158	-3,43356	3,0813	0,00206096	0,0274915	yes
CA6 (1 of 2)	groupXII:9898691-9902829	control	clone XII	3,44305	103,729	4,91299	-4,85997	1,17E-06	5,37E-05	yes
CA6 (2 of 2)	groupXII:9907407-9923316	control	clone XII	1,36533	41,7585	4,93474	-3,79045	0,000150373	0,00323204	yes
PFKM (1 of 2)	groupXII:10481807-10490116	control	clone XII	4,37929	0,209271	-4,38725	2,98857	0,00280291	0,0350929	yes
TNNT2 (1 of 2)	groupXII:12120259-12127679	control	clone XII	2,03777	371,584	7,51055	-4,0485	5,15E-05	0,00132201	yes
JPH2	groupXII:15320377-15331165	control	clone XII	1,59454	15,9748	3,32459	-3,20714	0,00134064	0,0194701	yes
ENSGACG00000012228	groupXII:16055913-16064592	control	clone XII	1,18329	23,1913	4,29271	-4,20161	2,65E-05	0,000756896	yes
ENSGACG00000012654	groupXII:16665952-16666789	control	clone XII	0,857158	179,67	7,71157	-3,9767	6,99E-05	0,00170479	yes
ENSGACG00000012657	groupXII:16668755-16670927	control	clone XII	3,66469	407,78	6,79796	-3,73619	0,000186833	0,00387162	yes
HSPB7	groupXII:5795326-5797360	control	clone XII	3,66327	152,038	5,37516	-3,46454	0,000531145	0,00917649	yes
PLEKHG5	groupXII:14799330-14819560	control	clone XII	2,53525	10,3397	2,028	-4,59658	4,29E-06	0,000162043	yes
MYL7	groupXIII:1859284-1860683	control	clone XII	5,31357	741,128	7,1239	-3,83892	0,000123576	0,00274436	yes
ENTPD4	groupXIII:11854910-11860754	control	clone XII	10,0272	21,0542	1,07019	-3,40573	0,000659864	0,0109496	yes
ADAMTS13	groupXIII:13880400-13888437	control	clone XII	6,34378	11,0225	0,797039	-5,08923	3,60E-07	1,95E-05	yes
KLF9	groupXIII:11679462-11681419	control	clone XII	11,7021	155,716	3,73408	-3,54391	0,000394239	0,00718671	yes
BMP10 (2 of 2)	groupXIV:6045851-6047708	control	clone XII	0,474013	25,6253	5,7565	-3,29288	0,000991652	0,015258	yes
TPM2	groupXIV:14793584-14812370	control	clone XII	58,3256	24,7439	-1,23705	8,15291	4,44E-16	2,06E-13	yes
ANO1 (1 of 2)	groupXIX:959941-986537	control	clone XII	5,53269	14,7195	1,41167	-3,15892	0,00158354	0,0222503	yes
TJP1 (1 of 2)	groupXIX:7580595-7597632	control	clone XII	2,06437	8,8901	2,10649	-2,93363	0,00335018	0,0404128	yes
TNNI2 (1 of 5)	groupXIX:9287887-9290073	control	clone XII	36,9595	1,71019	-4,43371	3,33945	0,000839432	0,0133171	yes
TNNI2 (3 of 5)	groupXIX:9291924-9297476	control	clone XII	72,7765	3,92816	-4,21155	3,59615	0,00032296	0,00609449	yes
ALPK3 (2 of 2)	groupXIX:12932395-12942731	control	clone XII	0,154534	3,47256	4,49	-3,09719	0,00195363	0,0263343	yes
LRRC10	groupXIX:18032150-18032915	control	clone XII	0,769411	24,6017	4,99886	-3,1908	0,00169482	0,0234988	yes
DENND4A (2 of 2)	groupXIX:6453753-6469506	control	clone XII	2,7655	45,001	4,02434	-4,38376	1,17E-05	0,000377426	yes
FLNC (2 of 2)	groupXIX:11818853-11840148	control	clone XII	0,669461	17,0993	4,67479	-3,43306	0,000596803	0,0100888	yes
KLF13	groupXIX:12552459-12562563	control	clone XII	5,31155	50,9135	3,26084	-3,00108	0,00269023	0,0339758	yes
ENSGACG00000011846	groupXIX:15110438-15111429	control	clone XII	432,416	34,067	-3,66598	3,16562	0,00154755	0,0218473	yes
HGF (1 of 2)	groupXIX:16212949-16226790	control	clone XII	0,939907	13,9528	3,89189	-2,93512	0,00333421	0,0402571	yes
ZC3HAV1	groupXIX:17534449-17539112	control	clone XII	35,2857	21,6113	-0,707299	3,00616	0,00264566	0,0335286	yes
ELL (2 of 2)	groupXV:8297534-8304173	control	clone XII	6,00553	53,2518	3,14847	-3,48949	0,000483939	0,00850258	yes
FERMT2	groupXV:8881764-8912733	control	clone XII	7,96454	20,0118	1,32919	-2,93676	0,00331657	0,0400882	yes
THBS1 (2 of 2)	groupXV:2296799-2303009	control	clone XII	5,34663	37,0327	2,7921	-3,19578	0,00139455	0,0200972	yes
ENSGACG00000006109	groupXV:2485490-2488657	control	clone XII	7,00411	168,097	4,58495	-2,88636	0,00389729	0,0455018	yes
ENSGACG00000009559	groupXV:7482605-7486604	control	clone XII	9,01124	78,2175	3,11869	-2,86557	0,00416257	0,0478894	yes
LRRFIP1 (1 of 2)	groupXVI:2173128-2210140	control	clone XII	33,9855	73,6969	1,11668	-4,70818	2,50E-06	0,000102155	yes
CASQ2 (1 of 2)	groupXVI:8508601-8514553	control	clone XII	2,91036	20,2521	2,7988	-3,60492	0,00031225	0,00592639	yes
MYL1	groupXVI:12804558-12809401	control	clone XII	63,654	3,31007	-4,26532	3,58378	0,000338662	0,00633876	yes
CPO (1 of 2)	groupXVI:15032955-15040739	control	clone XII	1,08007	11,1479	3,36757	-2,93108	0,00337791	0,0406752	yes
FHL2 (1 of 2)	groupXVI:7350266-7355490	control	clone XII	1,13922	21,1583	4,21511	-3,04155	0,00235361	0,0305561	yes
SIK1	groupXVI:7764692-7770611	control	clone XII	1,81871	20,9638	3,52691	-3,68744	0,000226519	0,00454395	yes
RGCC	groupXVI:17924002-17926186	control	clone XII	28,2047	157,304	2,47955	-2,88421	0,00392402	0,0457509	yes
SLC2A9	groupXVII:8676249-8728831	control	clone XII	16,4753	24,5507	0,575455	-3,01012	0,00261144	0,0331865	yes
SLC41A3 (1 of 2)	groupXVII:10995214-11001714	control	clone XII	0,495218	20,1056	5,34339	-3,26575	0,00109175	0,0165003	yes
ENSGACG00000005023	groupXVII:3249923-3258158	control	clone XII	15,2506	0,816368	-4,2235	3,39317	0,000690882	0,0113689	yes
ENSGACG00000011845	groupXVIII:14049189-14058105	control	clone XII	0,629198	14,6913	4,5453	-3,50348	0,000459224	0,00814659	yes
ENSGACG00000007661	groupXVIII:6547159-6551089	control	clone XII	2,14093	488,653	7,83442	-3,90291	9,50E-05	0,00220425	yes
HSP90AA1 (2 of 2)	groupXVIII:14758906-14763271	control	clone XII	0,284546	13,5565	5,57397	-3,25495	0,00113414	0,0170196	yes
ENSGACG00000010476	groupXVIII:10338804-10341156	control	clone XII	1,60684	68,2926	5,40943	-4,20233	2,64E-05	0,000754814	yes
CRIP2 (2 of 2)	groupXVIII:13111961-13116856	control	clone XII	2,78183	39,5776	3,83058	-2,90048	0,00372596	0,0439342	yes
ENSGACG00000013782	groupXVIII:16190238-16196307	control	clone XII	0,146639	8,39175	5,83863	-3,62568	0,000288207	0,00554683	yes
ENSGACG00000003030	groupXX:279325-280477	control	clone XII	50,9171	2822,85	5,79286	-3,17309	0,00150825	0,021398	yes
PAFAH1B3	groupXX:10901664-10911782	control	clone XII	55,2448	37,1866	-0,571056	6,31589	2,69E-10	3,68E-08	yes
ENSGACG00000012464	groupXX:13746909-13747781	control	clone XII	12,0529	140,599	3,54413	-4,07328	4,64E-05	0,00120915	yes
NR0B2	groupXX:8131362-8132271	control	clone XII	22,4858	2,61685	-3,10311	3,01805	0,00254404	0,0325081	yes
ENSGACG00000009825	groupXX:11526900-11530777	control	clone XII	51,1364	159,246	1,63884	-5,03898	4,68E-07	2,44E-05	yes
ENSGACG00000013326	groupXX:16113437-16115257	control	clone XII	21,0658	102,228	2,27882	-2,86003	0,00423595	0,0485501	yes
CSRNP1 (1 of 2)	groupXXI:8204340-8207525	control	clone XII	4,148	40,6604	3,29314	-3,5791	0,000344783	0,00643374	yes
CCR9 (1 of 2)	groupXXI:10240244-10243103	control	clone XII	2,60228	31,9553	3,61821	-3,18989	0,00142329	0,0204245	yes
MYOM1 (1 of 2)	groupXXI:9598720-9617679	control	clone XII	1,09627	20,2937	4,21035	-5,70918	1,14E-08	9,83E-07	yes
ENSGACG00000014852	scaffold_1014:540-2461	control	clone XII	56,6342	2208,16	5,28503	-2,93527	0,00333253	0,0402405	yes
ENSGACG00000010623	scaffold_133:23068-23662	control	clone XII	7,28838	1156,12	7,30947	-3,69672	0,000218405	0,00440972	yes
ENSGACG00000001073	scaffold_150:33953-42506	control	clone XII	0,772018	41,6602	5,75389	-3,57084	0,000355838	0,00660242	yes
CKMT2 (1 of 2)	scaffold_168:135091-142324	control	clone XII	4,79413	28,2488	2,55885	-3,71015	0,000207135	0,00421821	yes
ENSGACG00000001311	scaffold_255:7830-8361	control	clone XII	17,5203	0,972986	-4,17047	2,8685	0,0041242	0,0475451	yes
CYP27B1	scaffold_27:1199139-1205181	control	clone XII	7,50464	51,4143	2,77632	-3,62713	0,000286586	0,00552035	yes
FKBP5	scaffold_27:4190407-4197823	control	clone XII	16,0105	643,702	5,3293	-4,16292	3,14E-05	0,000872874	yes
ENSGACG00000001522	scaffold_27:4394391-4405173	control	clone XII	34,2684	1,79547	-4,25436	3,46033	0,00053952	0,00929369	yes
ENSGACG00000015110	scaffold_68:419739-424812	control	clone XII	6,49059	72,4721	3,481	-3,57348	0,000352268	0,00654712	yes
TNNC1	scaffold_68:530264-533797	control	clone XII	0,849758	314,511	8,53184	-4,74459	2,09E-06	8,77E-05	yes
CASQ1 (1 of 2)	scaffold_713:7518-10179	control	clone XII	20,5969	0,602345	-5,09569	4,01581	5,92E-05	0,00148545	yes
SNRNP70	scaffold_80:215861-221505	control	clone XII	164,659	268,48	0,705332	-4,6909	2,72E-06	0,000109908	yes
MPZL3	groupI:2301707-2308184	control	clone I	20,6773	0,719246	-4,84542	2,85613	0,00428842	0,0490083	yes
ENSGACG00000006790	groupI:4118508-4121716	control	clone I	26,7257	1,47477	-4,17966	4,78474	1,71E-06	7,40E-05	yes
ENSGACG00000008353	groupI:7086896-7088717	control	clone I	79,6185	249,28	1,64659	-3,05188	0,00227412	0,0297279	yes
SCARF1	groupI:7557007-7561871	control	clone I	1,35193	7,31467	2,43577	-2,91146	0,00359746	0,0427396	yes
ENSGACG00000009173	groupI:8598219-8599405	control	clone I	302,713	2,85227	-6,7297	5,05911	4,21E-07	2,23E-05	yes
DIABLO (1 of 2)	groupI:12409077-12411083	control	clone I	184,466	1,54478	-6,89981	5,28323	1,27E-07	7,95E-06	yes
CKM (1 of 2)	groupI:15913133-15919082	control	clone I	94,7437	10,9748	-3,10984	3,50488	0,000456821	0,00811188	yes
PIPOX	groupI:19754525-19779359	control	clone I	14,2234	0,492803	-4,85111	3,37524	0,000737502	0,0119907	yes
HPX	groupI:20820541-20824484	control	clone I	398,204	4,40762	-6,49736	7,85283	4,00E-15	1,59E-12	yes
ENSGACG00000014752	groupI:22723989-22724814	control	clone I	73,7738	4,62438	-3,99578	4,49122	7,08E-06	0,000247575	yes
ACADL	groupI:22725531-22729041	control	clone I	8,51763	0,710995	-3,58254	3,6128	0,000302908	0,00577917	yes
F2	groupI:27446959-27451949	control	clone I	300,719	5,32866	-5,8185	4,21883	2,46E-05	0,000709851	yes
ENSGACG00000015464	groupI:27507091-27513914	control	clone I	87,4185	1,38394	-5,98109	9,39489	0	0	yes
ENSGACG00000015472	groupI:27514140-27518203	control	clone I	138,882	1,27932	-6,76234	6,03991	1,54E-09	1,72E-07	yes
F10	groupI:27519217-27524787	control	clone I	95,2491	0,976222	-6,60835	5,60146	2,13E-08	1,69E-06	yes
PROZ (2 of 2)	groupI:27525490-27526891	control	clone I	101,777	1,6277	-5,96644	6,12034	9,34E-10	1,11E-07	yes
ENSGACG00000004413	groupI:65278-68049	control	clone I	461,416	3,45206	-7,06247	8,11995	4,44E-16	2,06E-13	yes
ENSGACG00000004822	groupI:514071-517686	control	clone I	32,7467	1,34519	-4,60546	3,83841	0,000123834	0,00274885	yes
ENSGACG00000005067	groupI:853150-864471	control	clone I	5,9233	0,694187	-3,09301	3,0595	0,00221705	0,029132	yes
ENSGACG00000005228	groupI:992280-1011125	control	clone I	70,0829	7,95403	-3,1393	3,06372	0,00218601	0,0288072	yes
CFB	groupI:2156319-2166868	control	clone I	76,4034	3,73169	-4,35573	4,43501	9,21E-06	0,000309066	yes
APOA1	groupI:2218129-2220146	control	clone I	7751,65	85,9806	-6,49435	3,04632	0,00231658	0,0301725	yes
ZFP36	groupI:7086896-7088717	control	clone I	96,7982	289,558	1,5808	-2,9206	0,00349363	0,0417529	yes
VTN (2 of 2)	groupI:7562562-7566990	control	clone I	52,3546	0,527891	-6,63193	8,5594	0	0	yes
SLC1A5	groupI:9146858-9153860	control	clone I	9,29285	52,7302	2,50444	-4,23594	2,28E-05	0,000665632	yes
SERPINF2	groupI:11927309-11930245	control	clone I	93,4635	1,34301	-6,12086	5,97217	2,34E-09	2,48E-07	yes
TTC36	groupI:15021861-15023385	control	clone I	135,287	7,47197	-4,17839	4,32926	1,50E-05	0,00046637	yes
FOXA3	groupI:16176221-16179190	control	clone I	16,4883	0,438711	-5,23203	4,18186	2,89E-05	0,000813889	yes
CYP2C9	groupI:16588423-16592719	control	clone I	511,423	7,2575	-6,1389	3,83073	0,000127764	0,00282169	yes
ABCB11	groupI:20584817-20596751	control	clone I	43,5289	0,214603	-7,66416	5,23733	1,63E-07	9,85E-06	yes
CD2	groupI:22023621-22036587	control	clone I	33,6114	18,3557	-0,85864	2,86208	0,00420864	0,0483043	yes
ENSGACG00000015411	groupI:27359084-27364279	control	clone I	132,116	0,73691	-7,4861	8,06308	6,66E-16	3,01E-13	yes
KYNU	groupI:27953680-27957166	control	clone I	9,8258	0,762041	-3,68863	3,18597	0,00144268	0,0206476	yes
PCSK6	groupII:3557485-3572495	control	clone I	7,29586	0,159974	-5,51117	3,8576	0,000114506	0,00257593	yes
METTL21C (2 of 2)	groupII:7652953-7655990	control	clone I	8,25195	0,337914	-4,61001	3,28633	0,00101504	0,0155482	yes
ENSGACG00000016298	groupII:14551247-14569051	control	clone I	21,2148	136,593	2,68674	-5,22434	1,75E-07	1,05E-05	yes
PACSIN3	groupII:21849504-21862280	control	clone I	6,30045	0,838581	-2,90943	3,0473	0,00230907	0,030093	yes
SLC27A2 (1 of 3)	groupII:5785622-5789071	control	clone I	14,2102	0,535006	-4,73123	4,34304	1,42E-05	0,000447399	yes
MPHOSPH10	groupII:8638771-8642484	control	clone I	21,2623	35,6582	0,745934	-3,35691	0,000788192	0,0126571	yes
LIPC	groupII:11310522-11317108	control	clone I	46,3473	0,820648	-5,81958	6,42973	1,28E-10	1,91E-08	yes
ENSGACG00000016168	groupII:13640059-13643928	control	clone I	126,25	9,26598	-3,7682	3,70914	0,000207965	0,00423221	yes
TNNT3 (2 of 2)	groupII:21934735-21940961	control	clone I	21,9067	2,20437	-3,31293	3,2861	0,00101584	0,0155582	yes
ENSGACG00000012390	groupIII:57692-62220	control	clone I	12,7787	2,487	-2,36126	4,60897	4,05E-06	0,000154115	yes
IGFBP1 (2 of 2)	groupIII:599860-601840	control	clone I	88,6685	2,21784	-5,31957	4,54726	5,43E-06	0,000197849	yes
ENSGACG00000014348	groupIII:3914290-3917139	control	clone I	118,266	3,11388	-5,24718	4,82948	1,37E-06	6,11E-05	yes
CYP7A1	groupIII:5080002-5084463	control	clone I	74,8458	4,59683	-4,02521	8,35227	0	0	yes
ANGPTL3 (1 of 2)	groupIII:9794900-9798464	control	clone I	16,4538	1,00918	-4,02717	4,02858	5,61E-05	0,00141944	yes
[illegible]	[illegible]	[illegible]	[illegible]	[illegible]	[illegible]	[illegible]	[illegible]	[illegible]	[illegible]	[illegible]
ENSGACG00000007329	groupXV:4313004-4316787	control	clone I	270,55	97,3532	-1,4746	4,96273	6,95E-07	3,43E-05	yes
ENSGACG00000007640	groupXV:4787065-4788482	control	clone I	84,4548	2,99436	-4,81786	4,27625	1,90E-05	0,000571725	yes
ENSGACG00000007642	groupXV:4790253-4796567	control	clone I	80,9656	0,85093	-6,57212	4,79305	1,64E-06	7,15E-05	yes
SERPINA10 (1 of 2)	groupXV:5380572-5382577	control	clone I	181,508	1,68212	-6,75361	7,93713	2,00E-15	8,36E-13	yes
ELL (2 of 2)	groupXV:8297534-8304173	control	clone I	6,00553	37,8133	2,65453	-4,04539	5,22E-05	0,00133663	yes
SLC2A2	groupXV:10147689-10155333	control	clone I	13,2943	0	-1,80E+308	-1,80E+308	0,00418103	0,0480541	yes
ENSGACG00000005133	groupXV:1444643-1453495	control	clone I	47,8789	23,6791	-1,01577	3,22318	0,00126775	0,0186172	yes
THBS1 (2 of 2)	groupXV:2296799-2303009	control	clone I	5,34663	41,8024	2,96688	-4,56442	5,01E-06	0,000184648	yes
QPCT	groupXV:3702226-3707282	control	clone I	23,1847	1,54089	-3,91134	3,73513	0,00018762	0,00388518	yes
GSTZ1	groupXV:4856329-4857793	control	clone I	39,6961	4,83307	-3,03798	3,09735	0,00195257	0,026323	yes
ACMSD	groupXVI:4108198-4112993	control	clone I	58,21	0,654498	-6,47473	4,43204	9,33E-06	0,000312622	yes
IGFBP2 (2 of 2)	groupXVI:5906186-5912186	control	clone I	13,3543	0,6515	-4,3574	2,90323	0,00369335	0,0436332	yes
ILDR1 (1 of 2)	groupXVI:7575142-7581994	control	clone I	15,9083	0,470008	-5,08095	4,57569	4,75E-06	0,00017641	yes
ENSGACG00000005482	groupXVI:10707621-10709196	control	clone I	32,1005	7,20026	-2,15647	2,88174	0,0039549	0,0460253	yes
MYL1	groupXVI:12804558-12809401	control	clone I	63,654	3,56941	-4,15649	4,29378	1,76E-05	0,000534414	yes
HNMT (2 of 2)	groupXVI:14288130-14290745	control	clone I	140,647	20,3489	-2,78906	3,24465	0,00117596	0,0175251	yes
ENSGACG00000007337	groupXVI:14344973-14348217	control	clone I	66,692	7,88015	-3,08122	3,49024	0,000482589	0,00848312	yes
FRZB	groupXVI:16050496-16058935	control	clone I	35,4299	10,383	-1,77075	3,46421	0,000531798	0,00918551	yes
ENSGACG00000001548	groupXVI:1606062-1610800	control	clone I	19,6502	70,7863	1,84892	-3,34222	0,0008311	0,0132074	yes
ENSGACG00000005264	groupXVI:10507461-10514195	control	clone I	37,5172	0,391003	-6,58423	3,38328	0,000716258	0,0117097	yes
UPP2	groupXVI:11169073-11170928	control	clone I	135,155	3,24694	-5,37939	4,48565	7,27E-06	0,000253128	yes
F5	groupXVI:17927939-17939340	control	clone I	43,205	0,676411	-5,99715	5,02372	5,07E-07	2,61E-05	yes
PPM1J	groupXVII:3666387-3676360	control	clone I	29,2041	77,9384	1,41616	-3,17173	0,00151534	0,0214775	yes
CYP24A1	groupXVII:3843198-3847380	control	clone I	219,445	4,68869	-5,54853	8,43503	0	0	yes
CELA1 (1 of 2)	groupXVII:4113562-4116634	control	clone I	13,4619	1,28765	-3,38608	2,92097	0,00348941	0,0417143	yes
SOAT2	groupXVII:5460811-5469767	control	clone I	31,0517	3,9413	-2,97793	3,90541	9,41E-05	0,00218518	yes
ENSGACG00000009188	groupXVII:8810501-8815093	control	clone I	3,93179	19,588	2,31671	-3,18624	0,00144135	0,0206325	yes
ENSGACG00000009200	groupXVII:8817602-8822247	control	clone I	4,25078	24,2117	2,50991	-4,44337	8,86E-06	0,000299218	yes
ENSGACG00000009583	groupXVII:9731456-9734766	control	clone I	37,2941	0,724762	-5,6853	4,82374	1,41E-06	6,26E-05	yes
ENSGACG00000009592	groupXVII:9737213-9738973	control	clone I	145,57	2,07824	-6,1302	6,69448	2,16E-11	3,93E-09	yes
ENSGACG00000010478	groupXVII:10724718-10741442	control	clone I	21,1269	9,01259	-1,22907	4,54562	5,48E-06	0,000199077	yes
G0S2	groupXVII:12665596-12666123	control	clone I	154,314	2,5631	-5,91183	4,99037	6,03E-07	3,03E-05	yes
ENSGACG00000005023	groupXVII:3249923-3258158	control	clone I	15,2506	0,394136	-5,27403	3,92559	8,65E-05	0,00203782	yes
ZNFX1	groupXVII:4531154-4544248	control	clone I	6,69213	18,1483	1,4393	-3,07339	0,00211641	0,0280832	yes
ALDH1L1	groupXVII:12102049-12111544	control	clone I	25,5644	1,74773	-3,87058	3,99461	6,48E-05	0,00160033	yes
ENSGACG00000011676	groupXVII:13185594-13190366	control	clone I	11,9876	42,33	1,82014	-3,08581	0,00203001	0,0271622	yes
CYP46A1	groupXVIII:2537070-2565520	control	clone I	69,4442	22,9854	-1,59514	3,79496	0,000147666	0,00318336	yes
PLG	groupXVIII:3113722-3122456	control	clone I	216,823	2,54515	-6,41262	8,41055	0	0	yes
ENSGACG00000006829	groupXVIII:5439706-5440561	control	clone I	73,2276	1,5468	-5,56502	5,28938	1,23E-07	7,73E-06	yes
ENSGACG00000006833	groupXVIII:5443127-5444747	control	clone I	53,0978	0	-1,80E+308	-1,80E+308	0,000126401	0,00279651	yes
LRG1	groupXVIII:9292141-9294935	control	clone I	72,6284	1,60911	-5,4962	5,22648	1,73E-07	1,04E-05	yes
APOB (5 of 5)	groupXVIII:9330964-9346541	control	clone I	397,857	2,52703	-7,29866	7,87156	3,55E-15	1,43E-12	yes
APOB (1 of 5)	groupXVIII:9364878-9366348	control	clone I	142,41	1,13425	-6,97217	6,68895	2,25E-11	4,07E-09	yes
IYD	groupXVIII:11202174-11205243	control	clone I	13,5345	1,15507	-3,55059	3,22459	0,00126153	0,0185457	yes
SLC22A16	groupXVIII:11846807-11852323	control	clone I	17,5664	0,209871	-6,38717	3,20753	0,0013388	0,0194484	yes
ENSGACG00000013449	groupXVIII:15749136-15756286	control	clone I	10,4827	0,756411	-3,7927	3,45814	0,000543925	0,00935562	yes
ENSGACG00000013769	groupXVIII:16170571-16176437	control	clone I	17,6243	0,180009	-6,61335	3,73859	0,000185053	0,00384112	yes
ENSGACG00000010476	groupXVIII:10338804-10341156	control	clone I	1,60684	32,4036	4,33386	-5,80928	6,27E-09	5,86E-07	yes
ALDH8A1	groupXVIII:11082283-11085044	control	clone I	54,0471	0,675786	-6,32151	4,8986	9,65E-07	4,54E-05	yes
ENSGACG00000011882	groupXVIII:12563265-12564805	control	clone I	17,522	0,890428	-4,29853	2,91898	0,00351179	0,0419258	yes
C6orf58	groupXVIII:15059227-15061479	control	clone I	111,847	2,00818	-5,7995	5,86065	4,61E-09	4,49E-07	yes
ENSGACG00000003030	groupXX:279325-280477	control	clone I	50,9171	0,928614	-5,77693	4,13477	3,55E-05	0,000967877	yes
ENSGACG00000003461	groupXX:754241-755023	control	clone I	2974,3	36,1844	-6,36104	4,7158	2,41E-06	9,90E-05	yes
ENSGACG00000003467	groupXX:759235-760746	control	clone I	11578,4	87,0044	-7,05614	3,4627	0,000534784	0,00922841	yes
ENSGACG00000003473	groupXX:762706-763550	control	clone I	397,148	4,91197	-6,33723	4,95348	7,29E-07	3,57E-05	yes
DPYS	groupXX:2879790-2889333	control	clone I	17,7264	0,554371	-4,99891	4,11414	3,89E-05	0,00104265	yes
HPN	groupXX:5115717-5118798	control	clone I	15,9595	0,434453	-5,19908	3,82259	0,000132058	0,00289982	yes
NCOA7 (2 of 2)	groupXX:5442148-5444002	control	clone I	16,9132	91,5327	2,43613	-3,47908	0,000503136	0,00877779	yes
CROT (2 of 2)	groupXX:10855977-10863256	control	clone I	24,6045	10,1067	-1,28362	4,84789	1,25E-06	5,65E-05	yes
ENSGACG00000012464	groupXX:13746909-13747781	control	clone I	12,0529	232,877	4,27211	-5,23745	1,63E-07	9,85E-06	yes
CNDP1	groupXX:2073451-2081405	control	clone I	39,708	0,25528	-7,2812	3,10953	0,00187385	0,0254777	yes
ENSGACG00000007038	groupXX:7875940-7881458	control	clone I	80,2886	0,457291	-7,45594	3,72387	0,000196193	0,00403245	yes
NR0B2	groupXX:8131362-8132271	control	clone I	22,4858	0	-1,80E+308	-1,80E+308	0,00178359	0,0244808	yes
ABCB4	groupXX:10864559-10876110	control	clone I	29,6888	0,234929	-6,98155	5,18274	2,19E-07	1,27E-05	yes
GAPDH (2 of 2)	groupXX:11781412-11785564	control	clone I	504,309	6,26794	-6,33017	5,91368	3,35E-09	3,39E-07	yes
PKLR	groupXX:12620831-12630510	control	clone I	10,0268	2,38317	-2,07291	3,18375	0,0014538	0,020775	yes
ENSGACG00000012473	groupXX:13875775-13876264	control	clone I	103,197	13,2353	-2,96294	2,96289	0,00304768	0,0375179	yes
PDIA4	groupXXI:5734018-5738065	control	clone I	24,0489	48,1773	1,00239	-2,93055	0,00338362	0,0407297	yes
PRHOXNB	groupXXI:7564025-7564669	control	clone I	97,3277	1,08533	-6,48664	4,69215	2,70E-06	0,000109325	yes
SLC22A13	groupXXI:8131606-8134827	control	clone I	7,87499	0,215097	-5,19422	3,26534	0,00109333	0,016519	yes
CSRNP1 (1 of 2)	groupXXI:8204340-8207525	control	clone I	4,148	23,7578	2,51791	-3,19575	0,00139468	0,0200985	yes
ABCF2	groupXXI:10006210-10011276	control	clone I	25,6315	86,4485	1,75392	-3,30907	0,000936081	0,0145583	yes
C8G	groupXXI:5566442-5569736	control	clone I	564,245	4,5427	-6,95663	6,08602	1,16E-09	1,34E-07	yes
HGD	groupXXI:5895781-5903328	control	clone I	30,7561	0,46214	-6,0564	4,55514	5,24E-06	0,000191702	yes
ENSGACG00000014852	scaffold_1014:540-2461	control	clone I	56,6342	2,57792	-4,45739	4,04776	5,17E-05	0,0013255	yes
ASGR1	scaffold_129:65701-71714	control	clone I	158,575	1,67779	-6,56246	5,52556	3,28E-08	2,47E-06	yes
TFR2	scaffold_129:135237-139395	control	clone I	7,74808	0,707518	-3,453	3,21178	0,00131914	0,0192224	yes
SLC13A5 (2 of 2)	scaffold_132:188482-197401	control	clone I	59,9029	0,849427	-6,13999	5,69695	1,22E-08	1,05E-06	yes
PROC	scaffold_135:36317-39014	control	clone I	52,1754	1,9043	-4,77604	5,20197	1,97E-07	1,16E-05	yes
ENSGACG00000017758	scaffold_137:194756-195795	control	clone I	0,712969	32,0601	5,49079	-3,06092	0,0022066	0,029023	yes
TYMP	scaffold_152:16045-20928	control	clone I	50,6855	1,68005	-4,915	4,79396	1,64E-06	7,12E-05	yes
TAT	scaffold_163:104982-110801	control	clone I	89,3102	1,81298	-5,62239	5,58991	2,27E-08	1,79E-06	yes
EGFR (2 of 2)	scaffold_182:13637-23312	control	clone I	12,4591	1,61848	-2,9445	3,43908	0,000583696	0,00990652	yes
GAPDH (1 of 2)	scaffold_208:92360-97479	control	clone I	102,905	10,9058	-3,23816	6,21181	5,24E-10	6,64E-08	yes
SLC22A14	scaffold_210:56746-62418	control	clone I	12,8704	1,15026	-3,48402	3,32523	0,000883453	0,0138823	yes
ENSGACG00000001261	scaffold_224:14139-22088	control	clone I	29,7889	1,49549	-4,31609	4,10537	4,04E-05	0,00107651	yes
ENSGACG00000001736	scaffold_232:280-4420	control	clone I	15,9236	2,43078	-2,71167	2,93061	0,00338299	0,0407239	yes
ENSGACG00000001742	scaffold_232:44158-79908	control	clone I	59,1722	8,092	-2,87035	8,95839	0	0	yes
C3 (7 of 8)	scaffold_232:167843-171745	control	clone I	25,5542	3,86762	-2,72404	3,16051	0,00157496	0,0221546	yes
C3 (8 of 8)	scaffold_251:15264-34678	control	clone I	24,4008	2,94383	-3,05116	11,69	0	0	yes
ENSGACG00000000614	scaffold_27:1359322-1367941	control	clone I	7,10486	13,2012	0,893794	-2,88451	0,00392023	0,0457139	yes
ENSGACG00000000665	scaffold_27:1704181-1705739	control	clone I	24,0092	2,09472	-3,51876	3,50375	0,000458751	0,00813988	yes
SLC25A34	scaffold_27:3240261-3248556	control	clone I	8,40527	0,847894	-3,30934	3,01074	0,00260614	0,0331358	yes
HYAL2 (1 of 2)	scaffold_27:3603786-3610736	control	clone I	14,5824	32,8338	1,17095	-2,98757	0,00281202	0,0351816	yes
MST1P9	scaffold_27:3770786-3781105	control	clone I	57,3718	0,501607	-6,83764	5,18914	2,11E-07	1,23E-05	yes
PFKM (2 of 2)	scaffold_27:1780298-1786604	control	clone I	16,1929	0,722596	-4,48603	4,41896	9,92E-06	0,000329235	yes
UROC1	scaffold_27:2259743-2266069	control	clone I	56,0871	1,70649	-5,03856	5,08932	3,59E-07	1,94E-05	yes
FKBP5	scaffold_27:4190407-4197823	control	clone I	16,0105	155,126	3,27635	-5,30202	1,15E-07	7,28E-06	yes
ITIH4 (2 of 2)	scaffold_27:4635810-4639569	control	clone I	704,296	11,2468	-5,96859	4,11775	3,83E-05	0,00102914	yes
ENSGACG00000001260	scaffold_332:1942-12505	control	clone I	319,286	3,67773	-6,43989	4,73807	2,16E-06	9,02E-05	yes
C3 (1 of 8)	scaffold_332:23002-24274	control	clone I	253,781	2,14946	-6,88346	5,29805	1,17E-07	7,41E-06	yes
C3 (2 of 8)	scaffold_332:25355-28790	control	clone I	100,881	2,30492	-5,45179	4,92365	8,49E-07	4,07E-05	yes
ENSGACG00000000231	scaffold_363:6365-16886	control	clone I	6,1495	0,315208	-4,28609	3,92053	8,84E-05	0,00207364	yes
ENSGACG00000001733	scaffold_609:4313-11347	control	clone I	387,005	2,88867	-7,0658	6,86878	6,48E-12	1,34E-09	yes
ALLC	scaffold_670:4194-8968	control	clone I	35,2693	1,24827	-4,82042	4,85961	1,18E-06	5,37E-05	yes
ENSGACG00000000272	scaffold_69:195180-196587	control	clone I	14,3081	1,01784	-3,81325	3,61718	0,000297829	0,00569912	yes
CASQ1 (1 of 2)	scaffold_713:7518-10179	control	clone I	20,5969	0,53228	-5,2741	3,65749	0,000254702	0,00500872	yes
ATP7B	scaffold_74:232711-239646	control	clone I	9,66025	0,151184	-5,99769	3,28491	0,00102015	0,0156127	yes
ENSGACG00000000007	scaffold_89:26629-27862	control	clone I	123,034	0,892176	-7,10752	5,61391	1,98E-08	1,59E-06	yes
EPS8	scaffold_90:181816-196703	control	clone I	5,62855	1,14029	-2,30336	3,37236	0,000745276	0,0120925	yes
ENSGACG00000001432	scaffold_908:2132-5789	control	clone I	818,086	3,13011	-8,0299	6,93316	4,12E-12	8,94E-10	yes
F3 (1 of 2)	scaffold_95:201021-206943	control	clone I	2,33133	20,0247	3,10255	-3,80198	0,000143546	0,00310933	yes
COX7A1	groupI:746198-747195	control	clone mix	34,9527	259,582	2,89271	-3,14181	0,00167906	0,0233233	yes
MPZL3	groupI:2301707-2308184	control	clone mix	20,6773	1,38496	-3,90013	3,37103	0,000748888	0,0121397	yes
ENSGACG00000006790	groupI:4118508-4121716	control	clone mix	26,7257	1,2251	-4,44725	4,08243	4,46E-05	0,00117029	yes
SGCG	groupI:4128095-4137512	control	clone mix	6,89238	26,5571	1,94602	-2,89446	0,00379814	0,0445955	yes
IGSF5 (2 of 2)	groupI:4236564-4248993	control	clone mix	11,5596	0,419673	-4,78368	3,329	0,000871581	0,0137298	yes
ENSGACG00000009173	groupI:8598219-8599405	control	clone mix	302,713	0,471193	-9,32742	4,80176	1,57E-06	6,89E-05	yes
DIABLO (1 of 2)	groupI:12409077-12411083	control	clone mix	184,466	0,46375	-8,63579	5,42268	5,87E-08	4,08E-06	yes
XIRP2 (1 of 2)	groupI:20491287-20509048	control	clone mix	0,545556	6,80076	3,63989	-3,95942	7,51E-05	0,00181064	yes
KBTBD10 (1 of 2)	groupI:20640583-20644716	control	clone mix	2,48134	37,7373	3,9268	-3,837	0,000124546	0,00276196	yes
HPX	groupI:20820541-20824484	control	clone mix	398,204	0,802277	-8,95519	7,1417	9,22E-13	2,31E-10	yes
SPEG (2 of 2)	groupI:21630257-21663404	control	clone mix	0,838689	5,55805	2,72837	-3,23453	0,00121844	0,0180301	yes
ENSGACG00000014752	groupI:22723989-22724814	control	clone mix	73,7738	1415,66	4,26222	-3,58549	0,000336452	0,00630562	yes
DMD	groupI:25729704-25866306	control	clone mix	0,594704	5,99977	3,33467	-3,67784	0,000235213	0,00468941	yes
F2	groupI:27446959-27451949	control	clone mix	300,719	2,17808	-7,10922	4,57973	4,66E-06	0,000173562	yes
ENSGACG00000015464	groupI:27507091-27513914	control	clone mix	87,4185	0,392757	-7,79816	5,95355	2,62E-09	2,74E-07	yes
ENSGACG00000015472	groupI:27514140-27518203	control	clone mix	138,882	0,463776	-8,22622	5,47421	4,39E-08	3,17E-06	yes
F10	groupI:27519217-27524787	control	clone mix	95,2491	0,466804	-7,67275	5,48701	4,09E-08	2,98E-06	yes
PROZ (2 of 2)	groupI:27525490-27526891	control	clone mix	101,777	0,348985	-8,18804	4,2316	2,32E-05	0,000676432	yes
ENSGACG00000004413	groupI:65278-68049	control	clone mix	461,416	0,619303	-9,54121	6,82082	9,05E-12	1,81E-09	yes
ENSGACG00000004822	groupI:514071-517686	control	clone mix	32,7467	1,68245	-4,28271	3,34954	0,000809453	0,0129317	yes
USP2	groupI:825334-835930	control	clone mix	1,73185	22,3637	3,69078	-3,78322	0,000154815	0,00331004	yes
CFB	groupI:2156319-2166868	control	clone mix	76,4034	2,06039	-5,21265	4,38593	1,15E-05	0,000374249	yes
APOA1	groupI:2218129-2220146	control	clone mix	7751,65	14,5024	-9,06207	4,21406	2,51E-05	0,000722663	yes
VTN (2 of 2)	groupI:7562562-7566990	control	clone mix	52,3546	2,73782	-4,25722	5,75123	8,86E-09	7,92E-07	yes
MLF1	groupI:8668668-8676804	control	clone mix	0,781018	21,7563	4,79993	-2,85732	0,00427236	0,0488688	yes
SLC1A5	groupI:9146858-9153860	control	clone mix	9,29285	68,2247	2,8761	-3,79753	0,000146143	0,00315609	yes
SERPINF2	groupI:11927309-11930245	control	clone mix	93,4635	0,409874	-7,83308	5,56731	2,59E-08	2,01E-06	yes
ENSGACG00000012590	groupI:16094777-16096616	control	clone mix	2,75754	34,1196	3,62915	-3,4605	0,000539175	0,00928892	yes
ENSGACG00000012592	groupI:16097382-16106309	control	clone mix	1,359	12,2607	3,17343	-3,33295	0,000859312	0,0135726	yes
CYP2C9	groupI:16588423-16592719	control	clone mix	511,423	5,27448	-6,59934	3,75529	0,000173139	0,00363377	yes
RYR1 (1 of 2)	groupI:16633943-16692160	control	clone mix	0,329757	7,14389	4,43723	-4,27817	1,88E-05	0,000567537	yes
ENSGACG00000013712	groupI:19969806-19974985	control	clone mix	5,23046	14,4244	1,4635	-2,90781	0,00363968	0,043131	yes
ABCB11	groupI:20584817-20596751	control	clone mix	43,5289	0,295882	-7,20081	4,8769	1,08E-06	4,99E-05	yes
GPM6B (2 of 2)	groupI:22269224-22284819	control	clone mix	14,2643	40,0413	1,48908	-3,19525	0,00139709	0,0201277	yes
RAMP1 (2 of 2)	groupI:22543147-22546037	control	clone mix	2,05393	45,1035	4,45678	-3,04966	0,00229099	0,0299031	yes
CLIC5 (2 of 2)	groupI:23417963-23421195	control	clone mix	1,12299	19,7114	4,13361	-3,37773	0,00073087	0,0119035	yes
SPP2	groupI:25542617-25544016	control	clone mix	50,2742	0,637666	-6,30087	3,71591	0,000202476	0,00413994	yes
ENSGACG00000015411	groupI:27359084-27364279	control	clone mix	132,116	0,194702	-9,40632	4,21198	2,53E-05	0,000728333	yes
PCSK6	groupII:3557485-3572495	control	clone mix	7,29586	0,196944	-5,21122	3,83884	0,000123615	0,00274499	yes
ENSGACG00000014922	groupII:5627137-5632419	control	clone mix	14,4191	273,147	4,24362	-4,22689	2,37E-05	0,000688445	yes
ADPRHL1	groupII:7768909-7772963	control	clone mix	0,339998	38,9887	6,84139	-4,82712	1,39E-06	6,17E-05	yes
RRAD	groupII:8210016-8212357	control	clone mix	2,13092	29,8949	3,81035	-3,22917	0,0012415	0,0183088	yes
CDH15	groupII:8468768-8479404	control	clone mix	0,410623	7,74803	4,23794	-3,40261	0,000667444	0,0110521	yes
ENSGACG00000016045	groupII:12989829-13015641	control	clone mix	1,3807	62,7757	5,50674	-4,53889	5,66E-06	0,000204499	yes
ENSGACG00000016298	groupII:14551247-14569051	control	clone mix	21,2148	124,818	2,55669	-4,06965	4,71E-05	0,00122545	yes
[illegible]	[illegible]	[illegible]	[illegible]	[illegible]	[illegible]	[illegible]	[illegible]	[illegible]	[illegible]	[illegible]
