# Supplementary material for: Specific Gene Expression Responses to Parasite Genotypes Reveal Redundancy of Innate Immunity in Vertebrates
Source: PLoS One. 2014 Sep 25;9(9):e108001. doi: 10.1371/journal.pone.0108001 (PMC4177871; doi:10.1371/journal.pone.0108001)
Supplement: Table S3 — (PDF) [file pone.0108001.s003.pdf]

Supplementary table S.3 Cufflinks output, list of differentially expressed genes in gill (g) tissue of three-spined sticklebacks. Differentially expressed is defined by comparison to uninfected controls. The term „gene“ is the name for a specific gene as taken from the *G. aculeatus* reference genome, „locus“ is the location on the genome, „sample_1“ is the control group, „sample_2“ is the infection treatment group, „value_1“ are FPKM values for „sample_1“, „value_2“ FPKM values for „sample_2“, log2(fold change) displays the transformed fold change in „sample_2“ compared to „sample_1“, the next three columns show the test statistic, p value and q value for each test, the last column shows whether the observed difference was significant (only significant differences shown).

gene	locus	sample_1	sample_2	value_1	value_2	log2(fold_cha	test_stat	p_value	q_value	significant
ENSGACG00000008353	groupI:7086896-7088717	control	clone XII	50,3064	263,255	2,38765	-3,3498	0,000808706	0,012922	yes
ZFP36	groupI:7086896-7088717	control	clone XII	60,4191	286,55	2,24571	-3,1527	0,00161768	0,0226335	yes
DHRS13 (1 of 3)	groupI:19518328-19523773	control	clone XII	108,393	38,3487	-1,49902	4,56292	5,04E-06	0,000185782	yes
NID2	groupI:20844309-20873790	control	clone XII	2,46445	6,30812	1,35594	-3,55866	0,000372754	0,00686296	yes
ENSGACG00000014109	groupII:473042-478908	control	clone XII	47,5235	176,247	1,89089	-2,8524	0,00433903	0,0494517	yes
PCDH20	groupII:7099597-7102887	control	clone XII	2,12969	13,209	2,63281	-3,1474	0,00164728	0,022971	yes
ADPRHL1	groupII:7768909-7772963	control	clone XII	0,973713	25,1604	4,69151	-3,83229	0,000126958	0,0028064	yes
ALPK3 (1 of 2)	groupII:15268483-15277781	control	clone XII	0,607306	6,71075	3,46598	-3,48465	0,000492782	0,00862902	yes
ENSGACG00000012663	groupIII:205052-212076	control	clone XII	1,68504	11,123	2,72269	-3,59056	0,00032997	0,00620401	yes
ENSGACG00000014948	groupIII:6319454-6331292	control	clone XII	0,748855	204,851	8,09567	-4,16889	3,06E-05	0,000853698	yes
ENSGACG00000014960	groupIII:6336030-6349511	control	clone XII	8,46975	49,6754	2,55214	-2,9573	0,00310345	0,0380444	yes
MYOM1 (2 of 2)	groupIII:12926418-12939638	control	clone XII	29,8104	53,5753	0,845752	-2,90411	0,00368303	0,0435377	yes
FOSL1	groupIV:5471462-5474199	control	clone XII	1,06039	22,6561	4,41723	-3,10783	0,00188469	0,0255974	yes
TSC22D3	groupIV:8520481-8545266	control	clone XII	34,2229	156,38	2,19202	-3,31443	0,000918313	0,0143307	yes
ACSL4	groupIV:15052252-15058258	control	clone XII	12,7385	28,8263	1,17819	-4,24316	2,20E-05	0,000647636	yes
ENSGACG00000018580	groupIV:15794115-15800925	control	clone XII	4,97784	24,6011	2,30513	-4,29601	1,74E-05	0,000529942	yes
LMOD2 (1 of 2)	groupIV:17997325-18001058	control	clone XII	3,49819	22,0856	2,65843	-3,62843	0,00028515	0,00549748	yes
NAV3	groupIV:25318370-25416178	control	clone XII	1,46705	9,68982	2,72356	-4,33458	1,46E-05	0,000456931	yes
SLC25A43	groupIV:5559504-5568467	control	clone XII	272,766	595,868	1,12733	-4,31428	1,60E-05	0,000494187	yes
FHL1 (1 of 2)	groupIV:6211431-6220387	control	clone XII	124,105	305,306	1,29869	-3,14521	0,00165965	0,0231076	yes
F8	groupIV:8344801-8357245	control	clone XII	5,13808	29,6584	2,52914	-3,33818	0,000843296	0,0133667	yes
GPX3	groupIV:13399030-13402595	control	clone XII	7,77586	57,3863	2,88363	-2,87865	0,00399387	0,0463754	yes
JUNB (2 of 2)	groupIX:15395448-15396453	control	clone XII	15,1382	63,0149	2,0575	-3,07201	0,00212623	0,0281882	yes
SORBS2	groupIX:5510578-5528882	control	clone XII	0,712431	12,4005	4,1215	-3,63341	0,000279704	0,00541025	yes
EDNRA	groupIX:6671382-6677679	control	clone XII	11,7722	51,1584	2,11959	-2,93187	0,00336931	0,0405912	yes
SMOX	groupIX:8508224-8518163	control	clone XII	7,5081	68,1363	3,1819	-7,55141	4,31E-14	1,42E-11	yes
DDIT4 (1 of 2)	groupV:834468-835775	control	clone XII	71,5376	923,628	3,69054	-4,7434	2,10E-06	8,82E-05	yes
SOCS3 (2 of 2)	groupV:2198432-2199203	control	clone XII	8,34668	47,5986	2,51164	-3,07035	0,00213805	0,0283133	yes
ENSGACG00000006530	groupV:8380543-8394690	control	clone XII	3,6377	14,3726	1,98222	-3,27455	0,00105829	0,0160868	yes
ENSGACG00000009715	groupV:11520658-11524884	control	clone XII	7,69285	20,9397	1,44465	-3,70777	0,000209094	0,00425162	yes
BAG3	groupVI:2938723-2942498	control	clone XII	5,59052	29,545	2,40186	-3,2849	0,00102019	0,0156133	yes
ADAM8 (1 of 2)	groupVI:8010221-8017184	control	clone XII	1,99632	14,0493	2,81508	-3,46488	0,000530467	0,00916727	yes
ENSGACG00000018840	groupVII:1415817-1425837	control	clone XII	7,80816	44,0267	2,49532	-5,15229	2,57E-07	1,46E-05	yes
ENSGACG00000019951	groupVII:8986295-8988589	control	clone XII	16,1539	85,6633	2,4068	-2,89957	0,0037367	0,0440323	yes
UCP2	groupVII:17373494-17381519	control	clone XII	6,97669	28,4305	2,02682	-3,10098	0,00192882	0,0260708	yes
ADAMTS15 (2 of 2)	groupVII:18219271-18228512	control	clone XII	1,72939	9,21868	2,4143	-2,86653	0,00414994	0,0477789	yes
ENSGACG00000018802	groupVII:1133366-1137045	control	clone XII	25,1082	112,963	2,16961	-3,65585	0,000256331	0,00503493	yes
ENSGACG00000019023	groupVII:2316335-2322112	control	clone XII	155,522	243,615	0,647488	-3,19399	0,00140321	0,0201973	yes
SLC3A2 (2 of 2)	groupVII:7463599-7468791	control	clone XII	57,6051	203,394	1,82001	-3,04591	0,0023198	0,0302066	yes
ENSGACG00000019819	groupVII:7739782-7745404	control	clone XII	330,117	95,5604	-1,78849	2,95049	0,00317272	0,0387159	yes
ENSGACG00000019952	groupVII:8986295-8988589	control	clone XII	20,2603	113,156	2,48159	-3,90422	9,45E-05	0,00219424	yes
ENSGACG00000020145	groupVII:12192535-12195407	control	clone XII	6,81232	60,9808	3,16214	-4,90661	9,27E-07	4,39E-05	yes
ENSGACG00000020852	groupVII:26873887-26878683	control	clone XII	7,63033	28,3403	1,89304	-3,15518	0,001604	0,0224793	yes
KLHL30	groupVIII:1730936-1734754	control	clone XII	6,39318	30,1863	2,23929	-3,43612	0,000590105	0,009995	yes
SLCO2A1	groupVIII:1818222-1828872	control	clone XII	11,3801	73,0033	2,68145	-4,07361	4,63E-05	0,00120776	yes
ENSGACG00000006048	groupVIII:6015396-6017317	control	clone XII	11,9513	76,9368	2,6865	-3,03342	0,00241797	0,0312234	yes
ENSGACG00000010050	groupVIII:12782195-12784724	control	clone XII	75,8464	356,62	2,23323	-4,51067	6,46E-06	0,000229088	yes
ABL2	groupVIII:13765906-13784759	control	clone XII	17,2845	56,791	1,71619	-2,94621	0,00321698	0,0391373	yes
GADD45B (1 of 2)	groupVIII:17587366-17588960	control	clone XII	37,5126	109,627	1,54716	-2,89483	0,0037936	0,0445552	yes
IGFBP1 (1 of 2)	groupVIII:3323546-3325070	control	clone XII	4,18529	28,4501	2,76504	-2,8579	0,00426454	0,0488	yes
MYLK4 (1 of 2)	groupVIII:3765987-3770589	control	clone XII	2,19647	27,7815	3,66086	-3,69668	0,000218435	0,00441022	yes
ARRDC2	groupVIII:9263821-9271184	control	clone XII	56,2373	305,752	2,44276	-3,15408	0,00161006	0,0225474	yes
ENSGACG00000011294	groupVIII:14348055-14365334	control	clone XII	311,59	892,933	1,5189	-4,39237	1,12E-05	0,000365142	yes
PPT2	groupX:3202821-3206898	control	clone XII	19,0794	70,2332	1,88014	-2,86336	0,00419174	0,0481513	yes
RNF19B	groupX:1428392-1440080	control	clone XII	7,9907	18,6609	1,22362	-3,42391	0,000617262	0,0103695	yes
FHOD3 (1 of 2)	groupX:11325772-11366320	control	clone XII	1,50954	8,19325	2,44033	-3,19577	0,00139457	0,0200974	yes
PDK2 (2 of 2)	groupXI:2022608-2030635	control	clone XII	14,6456	72,6066	2,30963	-3,52154	0,000429042	0,00770546	yes
ENSGACG00000007447	groupXI:4320058-4324681	control	clone XII	47,5903	14,7842	-1,68661	3,51511	0,000439578	0,00786092	yes
AOC2	groupXI:2185180-2193569	control	clone XII	4,95629	28,235	2,51015	-4,6096	4,03E-06	0,000153727	yes
CSNK1E (2 of 2)	groupXI:4260951-4268829	control	clone XII	27,8059	54,0266	0,95828	-3,09944	0,00193884	0,0261808	yes
TEF (1 of 2)	groupXI:4665255-4667381	control	clone XII	14,2325	66,4408	2,22288	-3,13793	0,00170145	0,0235716	yes
CBX7 (2 of 2)	groupXI:4748361-4752125	control	clone XII	15,2563	40,6416	1,41355	-3,37552	0,000736774	0,0119811	yes
NAPSA	groupXI:5068125-5071647	control	clone XII	20,8276	48,4569	1,21821	-5,05326	4,34E-07	2,29E-05	yes
SOCS3 (1 of 2)	groupXI:9066120-9067557	control	clone XII	25,2321	250,36	3,31067	-4,5713	4,85E-06	0,000179579	yes
MKL2 (2 of 2)	groupXI:13726296-13732972	control	clone XII	4,35613	18,2684	2,06823	-2,98702	0,00281709	0,0352331	yes
FMNL1 (2 of 2)	groupXI:14614250-14626268	control	clone XII	26,6017	40,5556	0,608382	-2,93727	0,00331117	0,0400362	yes
SCN4A (2 of 2)	groupXI:16076435-16099888	control	clone XII	0,783534	9,48819	3,59807	-4,45378	8,44E-06	0,000287059	yes
TNNT2 (1 of 2)	groupXII:12120259-12127679	control	clone XII	4,09459	297,459	6,18283	-5,12725	2,94E-07	1,64E-05	yes
ITGA5 (1 of 2)	groupXII:13865836-13893682	control	clone XII	1,8522	12,0105	2,69698	-3,01138	0,00260064	0,0330809	yes
CEBPB	groupXII:15595556-15596471	control	clone XII	38,5144	206,94	2,42574	-3,84504	0,000120531	0,00268807	yes
ENSGACG00000012657	groupXII:16668755-16670927	control	clone XII	3,19606	563,882	7,46295	-5,62532	1,85E-08	1,50E-06	yes
ENSGACG00000003612,STK35 (1 of 2)	groupXII:2314771-2325238	control	clone XII	13,3264	46,9256	1,81609	-5,20731	1,92E-07	1,13E-05	yes
MAD2L2	groupXII:5896524-5900121	control	clone XII	69,4241	23,9606	-1,53477	3,43981	0,000582133	0,0098846	yes
MMP9	groupXII:10243687-10248615	control	clone XII	36,6081	131,094	1,84036	-3,48901	0,000484807	0,00851444	yes
ENSGACG00000011767	groupXII:15482374-15483481	control	clone XII	1,98912	22,9263	3,52681	-2,91863	0,00351571	0,0419643	yes
MYL7	groupXIII:1859284-1860683	control	clone XII	4,22019	1081,87	8,00201	-5,04881	4,45E-07	2,33E-05	yes
GADD45G (1 of 3)	groupXIII:5635193-5636935	control	clone XII	19,0365	76,9361	2,01489	-3,45305	0,000554291	0,00949883	yes
KLF9	groupXIII:11679462-11681419	control	clone XII	10,8009	111,085	3,36244	-4,79182	1,65E-06	7,18E-05	yes
BMP1 (1 of 2)	groupXIII:12056150-12080196	control	clone XII	11,1381	35,1266	1,65706	-2,87931	0,00398548	0,0462977	yes
PDLIM2	groupXIII:12245916-12272753	control	clone XII	24,0273	45,7643	0,929548	-3,23712	0,00120742	0,0178988	yes
C6	groupXIV:10371246-10378853	control	clone XII	3,91636	10,9939	1,48911	-3,26709	0,00108661	0,0164358	yes
DAB2	groupXIV:1628592-1635733	control	clone XII	5,07582	14,08	1,47193	-4,00638	6,17E-05	0,00153559	yes
ENSGACG00000002902	groupXIX:2484536-2497605	control	clone XII	5,37291	39,9681	2,89507	-3,67728	0,000235732	0,00469788	yes
ENSGACG00000002933	groupXIX:2501528-2511962	control	clone XII	3,95259	39,8334	3,33311	-4,76216	1,92E-06	8,14E-05	yes
ENSGACG00000003003	groupXIX:2530796-2539506	control	clone XII	34,5327	385,531	3,48081	-2,96166	0,00305981	0,0376296	yes
SLC25A44 (2 of 2)	groupXIX:8533460-8537695	control	clone XII	47,2763	24,9795	-0,920373	3,48198	0,000497718	0,00869976	yes
ENSGACG00000008872	groupXIX:9986807-9990671	control	clone XII	45,0013	19,255	-1,22474	2,88114	0,00396237	0,0460921	yes
ALPK3 (2 of 2)	groupXIX:12932395-12942731	control	clone XII	1,80604	8,00938	2,14886	-2,90457	0,00367762	0,0434877	yes
ENSGACG00000004200	groupXIX:5960032-5963222	control	clone XII	14,8465	74,0679	2,31873	-3,50072	0,000464002	0,00821685	yes
DENND4A (2 of 2)	groupXIX:6453753-6469506	control	clone XII	0,605334	6,62834	3,45285	-3,9184	8,91E-05	0,00208854	yes
ANO9 (1 of 2)	groupXIX:7253474-7258006	control	clone XII	27,2713	16,2289	-0,748815	2,89568	0,00378342	0,044463	yes
PPP1R15B	groupXIX:8013636-8017213	control	clone XII	15,1615	64,9098	2,09803	-3,54197	0,000397146	0,00723149	yes
ENSGACG00000008779	groupXIX:9929495-9931303	control	clone XII	36,0468	18,1253	-0,99187	3,9443	8,00E-05	0,00190918	yes
EHF	groupXIX:14128181-14131907	control	clone XII	78,5776	19,4997	-2,01067	9,76523	0	0	yes
HGF (1 of 2)	groupXIX:16212949-16226790	control	clone XII	1,65226	13,7584	3,05781	-3,55949	0,000371571	0,0068446	yes
TMEM54 (1 of 2)	groupXV:4129199-4132227	control	clone XII	185,066	111,189	-0,735023	4,76415	1,90E-06	8,07E-05	yes
THBS1 (2 of 2)	groupXV:2296799-2303009	control	clone XII	2,65074	34,5915	3,70595	-4,24713	2,17E-05	0,000638121	yes
ENSGACG00000006109	groupXV:2485490-2488657	control	clone XII	5,19758	204,195	5,29596	-4,50709	6,57E-06	0,000232431	yes
ENSGACG00000009229	groupXV:7000917-7002139	control	clone XII	608,675	143,63	-2,08331	3,56185	0,000368246	0,00679296	yes
TRIM63	groupXVI:2043746-2051139	control	clone XII	12,979	55,0919	2,08566	-3,20417	0,00135454	0,0196346	yes
LRRFIP1 (1 of 2)	groupXVI:2173128-2210140	control	clone XII	13,4121	37,4832	1,48271	-5,28189	1,28E-07	8,00E-06	yes
OBSL1 (2 of 2)	groupXVI:5028195-5052297	control	clone XII	1,93798	6,14302	1,66439	-3,1555	0,00160222	0,0224604	yes
IRS2 (1 of 2)	groupXVI:7915269-7918611	control	clone XII	7,03819	29,5228	2,06855	-3,10279	0,00191706	0,0259422	yes
CASQ2 (1 of 2)	groupXVI:8508601-8514153	control	clone XII	8,45874	22,4406	1,40759	-2,95974	0,00307895	0,0378144	yes
GLB1L	groupXVI:11568968-11574338	control	clone XII	2,38445	9,88663	2,05182	-3,92567	8,65E-05	0,00203729	yes
ENSGACG00000005904	groupXVII:4156230-4161206	control	clone XII	5,05746	39,2117	2,9548	-4,45447	8,41E-06	0,000286309	yes
ENSGACG00000010478	groupXVII:10724718-10741442	control	clone XII	4,98245	19,7025	1,98345	-2,86906	0,00411691	0,04748	yes
SLC41A3 (1 of 2)	groupXVII:10995214-11001714	control	clone XII	0,831938	22,2157	4,73896	-3,41491	0,000638038	0,0106543	yes
ALPL	groupXVII:2447938-2457151	control	clone XII	8,11118	18,4409	1,18492	-2,91511	0,00355568	0,0423446	yes
ENSGACG00000008661,ENSGACG00000008663	groupXVII:8034011-8039296	control	clone XII	20,0964	36,9636	0,879168	-2,97232	0,00295562	0,0366216	yes
ENSGACG00000007661	groupXVII:6547159-6551089	control	clone XII	9,1662	480,502	5,71207	-4,16627	3,10E-05	0,000862009	yes
MRPS10	groupXVIII:8423428-8425214	control	clone XII	203,59	105,41	-0,949657	3,27384	0,00106097	0,0161199	yes
ACP1	groupXVIII:11653717-11661763	control	clone XII	288,366	150,671	-0,936496	3,16095	0,00157257	0,0221283	yes
HSP90AA1 (2 of 2)	groupXVIII:14758906-14763271	control	clone XII	2,48733	17,7599	2,83595	-3,26731	0,00108575	0,0164249	yes
ENSGACG00000010476	groupXVIII:10338804-10341156	control	clone XII	7,90894	140,49	4,15084	-6,39497	1,61E-10	2,33E-08	yes
RPS29	groupXVIII:14743703-14744593	control	clone XII	18752,2	9755,8	-0,94273	3,23122	0,00123265	0,0182012	yes
DIO3	groupXVIII:14792966-14793368	control	clone XII	10,7309	86,5018	3,01096	-3,40543	0,000660609	0,0109589	yes
ENSGACG00000013782	groupXVIII:16190238-16196307	control	clone XII	1,09741	15,0298	3,77565	-4,38576	1,16E-05	0,000374475	yes
KIAA0907	groupXX:12578997-12584703	control	clone XII	19,0056	39,204	1,04458	-3,63603	0,00027687	0,00536508	yes
KLHL38 (2 of 2)	groupXX:5618665-5622627	control	clone XII	3,80745	31,7783	3,06114	-3,84906	0,000118572	0,00265181	yes
C4A	groupXX:9276660-9289490	control	clone XII	26,6354	59,4999	1,15954	-5,83678	5,32E-09	5,08E-07	yes
ENSGACG00000009520,ENSGACG00000009524	groupXX:11267260-11284958	control	clone XII	66,678	134,042	1,0074	-2,9619	0,0030575	0,0376081	yes
ENSGACG00000009825	groupXX:11526900-11530777	control	clone XII	16,3629	77,094	2,23619	-5,22212	1,77E-07	1,06E-05	yes
NR1D2 (2 of 2)	groupXX:14965102-14970216	control	clone XII	20,0526	111,984	2,48143	-3,73097	0,000190746	0,00393907	yes
ENSGACG00000013327	groupXX:16118213-16120496	control	clone XII	2,56854	25,6165	3,31805	-3,64792	0,000264369	0,00516375	yes
BAG6 (2 of 2)	groupXX:16125096-16135132	control	clone XII	21,5661	31,1587	0,530867	-2,87391	0,0040542	0,0469169	yes
ENSGACG00000003464	groupXXI:8029733-8038141	control	clone XII	3,94381	10,7657	1,44878	-2,89779	0,00375806	0,0442279	yes
SLC4A2 (3 of 3)	groupXXI:10036568-10048067	control	clone XII	1,31913	7,5689	2,5205	-3,64605	0,000266301	0,00519505	yes
WAC (1 of 2)	groupXXI:557602-579035	control	clone XII	13,0304	29,4546	1,17662	-3,89902	9,66E-05	0,00223429	yes
ENSGACG00000004079	groupXXI:9448708-9457454	control	clone XII	1,42339	32,1966	4,49951	-4,45375	8,44E-06	0,00028709	yes
BAG6 (1 of 2)	scaffold_101:157721-177579	control	clone XII	8,76521	15,7923	0,849364	-3,1912	0,00141682	0,0203509	yes
ENSGACG00000010623	scaffold_133:23068-23662	control	clone XII	3,27134	1400,79	8,74215	-5,38181	7,37E-08	4,97E-06	yes
TG	scaffold_157:35547-44817	control	clone XII	3,25152	18,8399	2,53461	-3,12325	0,00178864	0,0245357	yes
ITGB1 (1 of 2)	scaffold_169:109603-124646	control	clone XII	35,9397	55,5941	0,629352	-4,21146	2,54E-05	0,000729761	yes
ENSGACG00000010501	scaffold_185:398-145602	control	clone XII	108,909	37,7797	-1,52744	4,57405	4,78E-06	0,000177573	yes
EIF5B	scaffold_210:40893-49514	control	clone XII	12,4233	20,4365	0,718096	-3,32644	0,000879615	0,0138336	yes
TIMELESS	scaffold_27:1143833-1158386	control	clone XII	1,15246	12,361	3,42301	-3,03017	0,00244414	0,0314901	yes
VWA1 (2 of 2)	scaffold_27:3166036-3170998	control	clone XII	77,0431	44,9176	-0,778383	7,10095	1,24E-12	3,03E-10	yes
PTGIS (2 of 2)	scaffold_27:3833602-3840073	control	clone XII	38,8	123,16	1,66641	-3,04271	0,00234458	0,0304657	yes
KCNB1	scaffold_27:3843350-3862320	control	clone XII	3,70186	37,9268	3,35689	-5,51314	3,52E-08	2,62E-06	yes
FKBP5	scaffold_27:4190407-4197823	control	clone XII	21,4832	479,394	4,47993	-5,68876	1,28E-08	1,09E-06	yes
SVIL (1 of 2)	scaffold_37:1988686-2023111	control	clone XII	4,54125	10,2776	1,17834	-2,98989	0,00279078	0,0349739	yes
ENSGACG00000001749	scaffold_434:1322-4701	control	clone XII	2,47946	19,4573	2,97222	-3,59272	0,000327239	0,00616061	yes
ENSGACG00000000208	scaffold_459:10291-13858	control	clone XII	1,97336	21,4346	3,44122	-3,97358	7,08E-05	0,00172361	yes
PTRF (1 of 2)	scaffold_48:1611748-1628972	control	clone XII	11,0336	41,6631	1,91687	-3,46949	0,000521443	0,00903934	yes
TNNC1	scaffold_68:530264-533797	control	clone XII	2,48825	256,551	6,68797	-5,17049	2,33E-07	1,34E-05	yes
ENSGACG00000000300	scaffold_69:420454-477489	control	clone XII	15,6926	73,0151	2,21811	-3,18148	0,00146525	0,0209078	yes
ENSGACG00000000272	scaffold_69:195180-196587	control	clone XII	48,5113	196,966	2,02155	-3,38432	0,000713554	0,011674	yes
MYLK	scaffold_69:420454-477489	control	clone XII	6,77157	25,454	1,91033	-2,95588	0,00311783	0,03818	yes
ENSGACG00000000849	scaffold_863:4798-7415	control	clone XII	1,56157	15,2263	3,2855	-3,07291	0,00211981	0,0281181	yes
ENSGACG00000008353	groupI:7086896-7088717	control	clone I	50,3064	152,828	1,60309	-3,21835	0,00128929	0,0188717	yes
ENSGACG00000009417	groupI:9066262-9069851	control	clone I	33,9764	96,728	1,5094	-3,18855	0,00142986	0,0204997	yes
ZFP36	groupI:7086896-7088717	control	clone I	60,4191	178,19	1,56034	-3,3816	0,00072066	0,0117672	yes
ADPRHL1	groupII:7768909-7772963	control	clone I	0,973713	10,4742	3,4272	-2,88171	0,00395527	0,0460285	yes
BTBD10 (1 of 2)	groupII:10369562-10375886	control	clone I	42,4814	33,5504	-0,3405	3,20409	0,0013549	0,0196386	yes
ENSGACG00000015897	groupII:11731640-11741866	control	clone I	290,256	210,086	-0,466343	10,5902	0	0	yes
PAQR5	groupII:18965510-18973830	control	clone I	50,2725	32,6755	-0,621558	3,89814	9,69E-05	0,00224123	yes
ENSGACG00000014948	groupIII:6319454-6331292	control	clone I	0,748855	45,4512	5,92349	-6,0245	1,70E-09	1,87E-07	yes
RGS13 (2 of 2)	groupIII:9436194-9438496	control	clone I	33,8673	87,5836	1,37077	-2,86336	0,00419181	0,0481519	yes
COL11A1 (2 of 2)	groupIII:11565982-11627261	control	clone I	2,11762	7,57361	1,83854	-3,22625	0,00125422	0,0184586	yes
CEBPD	groupIII:16376601-16377524	control	clone I	221,489	636,103	1,52203	-3,18623	0,00144139	0,0206331	yes
TSC22D3	groupIV:8520481-8545266	control	clone I	34,2229	155,332	2,18233	-4,48824	7,18E-06	0,000250547	yes
CASP3 (4 of 4)	groupIV:17650265-17655564	control	clone I	450,008	269,981	-0,73709	3,80738	0,000140448	0,00305316	yes
ENSGACG00000019744	groupIV:29692169-29694681	control	clone I	72,2423	167,575	1,21389	-3,48334	0,000495206	0,00866407	yes
SLC25A43	groupIV:5559504-5568467	control	clone I	272,766	514,594	0,915771	-4,26234	2,02E-05	0,000602634	yes
MYBPC1	groupIV:30979682-30996951	control	clone I	18,1733	25,4958	0,488438	-4,20536	2,61E-05	0,000746637	yes
NDUFS8 (2 of 2)	groupIX:4125131-4133669	control	clone I	187,361	150,683	-0,314304	4,30529	1,67E-05	0,000511479	yes
ENSGACG00000018993	groupIX:15088279-15089729	control	clone I	344,064	125,179	-1,45868	3,45266	0,000555097	0,00950987	yes
SLC8A1 (2 of 2)	groupIX:19919460-19931018	control	clone I	0	9,28237	1,80E+308	1,80E+308	0,000275115	0,00533715	yes
ENSGACG00000016379	groupIX:3210035-3214271	control	clone I	23,9364	48,1527	1,00841	-3,16222	0,00156572	0,0220528	yes
SMOX	groupIX:8508224-8518163	control	clone I	7,5081	24,6879	1,71728	-4,71066	2,47E-06	0,000101125	yes
ENSGACG00000019252	groupIX:16121523-16124104	control	clone I	38,0484	94,8713	1,31814	-3,01611	0,00256043	0,0326731	yes
DDIT4 (1 of 2)	groupV:834468-835775	control	clone I	71,5376	801,165	3,48533	-6,69833	2,11E-11	3,84E-09	yes
ENTPD1	groupV:1673682-1685493	control	clone I	43,7735	57,9659	0,405147	-3,36887	0,000754777	0,0122171	yes
ENSGACG00000009409	groupV:11304062-11306049	control	clone I	60,862	359,262	2,56142	-3,37859	0,000728758	0,0118543	yes
COL1A1 (2 of 2)	groupV:3433394-3454716	control	clone I	120,383	195,736	0,701278	-3,1774	0,001486	0,0211432	yes
ACE	groupVI:11674618-11688534	control	clone I	7,8532	17,9434	1,1921	-4,51454	6,35E-06	0,000225561	yes
CYP1B1	groupVI:2417128-2420132	control	clone I	21,7909	53,6428	1,29966	-2,8951	0,00379036	0,0445283	yes
UBTD1 (2 of 2)	groupVI:8353732-8359824	control	clone I	36,8727	19,0522	-0,9526	3,70876	0,000208281	0,00423736	yes
SULT6B1	groupVI:14351689-14358209	control	clone I	23,3	179,706	2,94725	-4,69467	2,67E-06	0,000108152	yes
ENSGACG00000018557,ENSGACG00000018560	groupVII:127090-135192	control	clone I	72,4599	126,103	0,799344	-5,45935	4,78E-08	3,41E-06	yes
ENSGACG00000018840	groupVII:1415817-1425837	control	clone I	7,80816	34,3123	2,13567	-4,90975	9,12E-07	4,32E-05	yes
SLC7A8	groupVII:2888331-2902598	control	clone I	18,4292	32,9228	0,837094	-3,65115	0,000261067	0,00511129	yes
ENSGACG00000020181	groupVII:12872156-12875169	control	clone I	7,77078	39,0872	2,33058	-4,69983	2,60E-06	0,000105829	yes
ASL	groupVII:19028240-19050014	control	clone I	50,9574	32,7955	-0,635794	2,97306	0,00294843	0,0365495	yes
DYNLL1	groupVII:21537345-21538705	control	clone I	155,314	92,8507	-0,742203	4,28614	1,82E-05	0,000550311	yes
ENSGACG00000018802	groupVII:1133366-1137045	control	clone I	25,1082	96,307	1,93948	-4,294	1,75E-05	0,000533964	yes
ENSGACG00000019023	groupVII:2316335-2322112	control	clone I	155,522	212,996	0,453711	-3,49765	0,000469374	0,00829426	yes
ENSGACG00000019952	groupVII:8986295-8988589	control	clone I	20,2603	58,3928	1,52714	-2,96814	0,00299605	0,0370231	yes
ENSGACG00000020145	groupVII:12192535-12195407	control	clone I	6,81232	37,8291	2,47328	-4,64048	3,48E-06	0,000135431	yes
TIPRL	groupVII:12553814-12557405	control	clone I	85,6319	66,2415	-0,370412	3,29567	0,000981861	0,0151353	yes
F11R	groupVII:18366838-18372450	control	clone I	282,828	187,116	-0,595993	3,60495	0,000312221	0,00592568	yes
SLCO2A1	groupVIII:1818222-1828872	control	clone I	11,3801	30,8305	1,43784	-2,93061	0,00338298	0,0407239	yes
ENSGACG00000006048	groupVIII:6015396-6017317	control	clone I	11,9513	60,3035	2,33507	-3,55757	0,000374297	0,00688626	yes
ENSGACG00000010050	groupVIII:12782195-12784724	control	clone I	75,8464	207,112	1,44932	-2,96708	0,00300704	0,0371464	yes
SELE	groupVIII:15021466-15028887	control	clone I	3,9964	13,5434	1,76081	-3,33765	0,000844904	0,0133884	yes
GADD45B (1 of 2)	groupVIII:17587366-17588960	control	clone I	37,5126	91,567	1,28745	-3,50956	0,000448855	0,00799541	yes
PDGFRA	groupVIII:18671403-18683235	control	clone I	9,20952	18,4715	1,0041	-3,41096	0,000647347	0,010791	yes
ENSGACG00000006044	groupVIII:6015396-6017317	control	clone I	7,57242	68,6483	3,1804	-4,44766	8,68E-06	0,000294079	yes
ARRDC2	groupVIII:9263821-9271184	control	clone I	56,2373	307,302	2,45006	-5,27545	1,32E-07	8,25E-06	yes
CDCP1 (1 of 3),CDCP1 (3 of 3)	groupX:3789001-3798174	control	clone I	36,367	19,5237	-0,897406	2,91529	0,00355355	0,0423252	yes
ENSGACG00000007447	groupXI:4320058-4324681	control	clone I	47,5903	12,4038	-1,93988	5,13016	2,89E-07	1,62E-05	yes
ENSGACG00000007449	groupXI:4378240-4381177	control	clone I	49,7487	10,7997	-2,20367	4,42218	2,41E-05	0,000698223	yes
AOC2	groupXI:2185180-2193569	control	clone I	4,95629	14,6364	1,56223	-3,09746	0,00195189	0,0263151	yes
ENSGACG00000007454	groupXI:4500951-4505292	control	clone I	19,1988	6,96127	-1,46359	2,98523	0,00283364	0,0354025	yes
SOCS3 (1 of 2)	groupXI:9066120-9067557	control	clone I	25,2321	151,034	2,58154	-5,44358	5,22E-08	3,68E-06	yes
CANT1 (1 of 2)	groupXI:9138284-9143907	control	clone I	100,172	63,3602	-0,659222	2,94005	0,00328156	0,0397541	yes
PCTP	groupXI:15198609-15203999	control	clone I	136,089	96,8768	-0,490294	3,78145	0,000155918	0,00332977	yes
TNNT2 (1 of 2)	groupXII:12120259-12127679	control	clone I	4,09459	154,97	5,24213	-5,57942	2,41E-08	1,89E-06	yes
CEBPB	groupXII:15595556-15596471	control	clone I	38,5144	144,65	1,90904	-4,27259	1,93E-05	0,000579733	yes
ENSGACG00000012654	groupXII:16665952-16666789	control	clone I	0	133,412	1,80E+308	1,80E+308	1,31E-06	5,90E-05	yes
ENSGACG00000012657	groupXII:16668755-16670927	control	clone I	3,19606	261,003	6,35163	-5,95851	2,55E-09	2,67E-07	yes
SULT1E1	groupXII:1048503-1051783	control	clone I	100,525	77,559	-0,374183	3,52641	0,000421237	0,00758966	yes
ENSGACG00000003612,STK35 (1 of 2)	groupXII:2314771-2325238	control	clone I	13,3264	40,7432	1,61227	-4,03183	5,53E-05	0,00140301	yes
EPHA2 (1 of 2)	groupXII:5828450-5848775	control	clone I	24,5537	18,464	-0,411225	3,22585	0,00125599	0,0184807	yes
ENSGACG00000011767	groupXII:15482374-15483481	control	clone I	1,98912	15,7012	2,98067	-2,87822	0,00399931	0,0464287	yes
MYL7	groupXIII:1859284-1860683	control	clone I	4,22019	1059,85	7,97234	-6,30546	2,87E-10	3,91E-08	yes
ENSGACG00000003503	groupXIII:424304-426146	control	clone I	107,721	22,4894	-2,25998	4,26478	2,00E-05	0,000597145	yes
GCNT1	groupXIII:8044619-8046408	control	clone I	0,729881	98,149	7,07117	-4,56459	5,00E-06	0,000184514	yes
KLF9	groupXIII:11679462-11681419	control	clone I	10,8009	73,6683	2,76989	-4,90646	9,27E-07	4,39E-05	yes
PIK3R1 (1 of 2)	groupXIV:1222717-1239465	control	clone I	32,8272	76,9047	1,22818	-3,80019	0,000144583	0,00312788	yes
ENSGACG00000017093	groupXIV:6818761-6821900	control	clone I	27,3379	40,7239	0,574976	-3,18429	0,00145109	0,020743	yes
ENSGACG00000002902	groupXIX:2484536-2497605	control	clone I	5,37291	53,1071	3,30513	-4,90433	9,37E-07	4,43E-05	yes
ENSGACG00000002933	groupXIX:2501528-2511962	control	clone I	3,95259	46,592	3,55921	-5,44172	5,28E-08	3,72E-06	yes
ENSGACG00000002955	groupXIX:2513167-2523323	control	clone I	21,0012	276,3	3,71769	-21,5273	0	0	yes
ENSGACG00000003003	groupXIX:2530796-2539506	control	clone I	34,5327	377,214	3,44935	-5,98758	2,13E-09	2,28E-07	yes
CKMT1A	groupXIX:8174514-8181627	control	clone I	20,2189	39,5468	0,96786	-3,28692	0,00101288	0,0155223	yes
ENSGACG00000002962	groupXIX:16699629-16700761	control	clone I	2,03101	108,076	5,7337	-4,05452	5,02E-05	0,00129382	yes
ENSGACG00000004200	groupXIX:5960032-5963222	control	clone I	14,8465	217,445	3,87245	-5,66666	1,46E-08	1,22E-06	yes
ENSGACG00000004203	groupXIX:5991571-5993364	control	clone I	0	25,6898	1,80E+308	1,80E+308	0,000454677	0,00807937	yes
PPP1R15B	groupXIX:8013636-8017213	control	clone I	15,1615	41,9031	1,46665	-3,43914	0,000583557	0,00990473	yes
EIF4G2 (1 of 2)	groupXIX:10640524-10650421	control	clone I	51,2644	67,4917	0,396751	-3,03665	0,00239283	0,0433112	yes
FLNC (2 of 2)	groupXIX:11818853-11840148	control	clone I	8,06818	21,4842	1,41296	-5,16736	2,37E-07	1,36E-05	yes
KLF13	groupXIX:12552459-12562563	control	clone I	0	50,9034	1,80E+308	1,80E+308	0,000386751	0,00707433	yes
EHF	groupXIX:14128181-14131907	control	clone I	78,5776	45,9294	-0,774701	3,824	0,000131196	0,00285734	yes
THBS1 (2 of 2)	groupXV:2296799-2303009	control	clone I	2,65074	14,2046	2,42189	-4,41671	1,00E-05	0,000332163	yes
ENSGACG00000006109	groupXV:2485490-2488657	control	clone I	5,19758	56,7884	3,44969	-4,83351	1,34E-06	6,01E-05	yes
IDH1	groupXV:17770455-17775942	control	clone I	119,821	83,8107	-0,515671	2,89973	0,00373478	0,0440211	yes
ENSGACG00000003420	groupXVI:7720530-7724761	control	clone I	80,8451	32,3254	-1,32249	3,29217	0,000994	0,0152893	yes
ENSGACG00000005904	groupXVII:4156230-4161206	control	clone I	5,05746	17,9092	1,82422	-3,49482	0,000474513	0,00836959	yes
ENSGACG00000007661	groupXVII:6547159-6551089	control	clone I	9,1662	137,088	3,90263	-5,2886	1,23E-07	7,79E-06	yes
NFKBIA (1 of 2)	groupXVIII:1969539-1972310	control	clone I	101,293	256,941	1,3429	-3,33047	0,000866986	0,0136703	yes
ENSGACG00000010476	groupXVIII:10338804-10341156	control	clone I	7,90894	77,0277	3,28382	-6,52672	6,72E-11	1,08E-08	yes
DIO3	groupXVIII:14792966-14793368	control	clone I	10,7309	53,1938	2,30949	-3,02741	0,00246666	0,0317198	yes
NCOA7 (2 of 2)	groupXX:5442148-5444002	control	clone I	75,0603	26,1798	-1,51921	3,27578	0,00105372	0,0160297	yes
FCGBP (1 of 2)	groupXX:9159953-9170909	control	clone I	33,2968	65,2253	0,905723	-3,01224	0,00259327	0,0330062	yes
TUFT1 (2 of 2)	groupXX:13275796-13285064	control	clone I	54,1856	101,515	0,905713	-3,51924	0,000432806	0,00776053	yes
PNISR	groupXX:3265740-3273201	control	clone I	30,5305	48,4171	0,665106	-3,32515	0,000883694	0,0138953	yes
C4A	groupXX:9276660-9289490	control	clone I	26,6354	44,8943	0,753185	-3,20909	0,00133154	0,0193541	yes
NR1D2 (2 of 2)	groupXX:14965102-14970216	control	clone I	20,0526	119,28	2,57249	-5,81914	5,92E-09	5,57E-07	yes
ENSGACG00000013327	groupXX:16118213-16120496	control	clone I	2,56854	19,9933	2,9605	-3,78685	0,000152568	0,00327108	yes
FZD1	groupXXI:18887910-18889485	control	clone I	32,5759	13,0693	-1,31523	2,86873	0,00412123	0,0475169	yes
CCR9 (1 of 2)	groupXXI:10240244-10243103	control	clone I	15,7446	43,8927	1,47913	-3,59594	0,000323218	0,00609843	yes
ENSGACG00000004079	groupXXI:9448708-9457454	control	clone I	1,42339	16,3315	3,52026	-4,71636	2,40E-06	9,88E-05	yes
MYOM1 (1 of 2)	groupXXI:9598720-9617679	control	clone I	8,20821	15,9489	0,958314	-3,14872	0,0016399	0,0228865	yes
ENSGACG00000010623	scaffold_133:23068-23662	control	clone I	3,27134	646,134	7,62581	-6,07484	1,24E-09	1,42E-07	yes
TG	scaffold_157:35547-44817	control	clone I	3,25152	13,5753	2,06179	-3,15575	0,00160065	0,0224392	yes
ENSGACG00000018049	scaffold_164:207395-210089	control	clone I	4,94558	153,111	5,07397	-3,79518	0,000147534	0,00318105	yes
ENSGACG00000010501	scaffold_185:398-145602	control	clone I	108,909	49,6984	-1,13185	4,79812	1,60E-06	6,98E-05	yes
NEB	scaffold_202:3792-65875	control	clone I	13,3165	19,3005	0,579602	-3,69123	0,000223176	0,00448927	yes
ENSGACG00000000614	scaffold_27:1359322-1367941	control	clone I	13,1427	55,3434	2,07415	-8,03576	8,88E-16	3,93E-13	yes
VWA1 (2 of 2)	scaffold_27:3166036-3170998	control	clone I	77,0431	40,8314	-0,915985	4,87272	1,10E-06	5,08E-05	yes
GATA2 (1 of 2)	scaffold_27:2199380-2206673	control	clone I	11,0657	64,4838	2,54288	-4,05452	5,02E-05	0,00129391	yes
FKBP5	scaffold_27:4190407-4197823	control	clone I	21,4832	365,986	4,09051	-6,82313	8,91E-12	1,78E-09	yes
ENSGACG00000001198	scaffold_419:5110-7209	control	clone I	1,79675	37,243	4,37351	-3,20918	0,00133122	0,0193559	yes
ENSGACG00000001749	scaffold_434:1322-4701	control	clone I	2,47946	18,6597	2,91183	-3,799	0,000145292	0,00314077	yes
ENSGACG00000001763	scaffold_434:8775-11798	control	clone I	2,65739	23,3681	3,13646	-4,83376	1,34E-06	6,00E-05	yes
ENSGACG00000000208	scaffold_459:10291-13858	control	clone I	1,97336	15,9716	3,01676	-3,41469	0,000638552	0,0106607	yes
ENSGACG00000001671	scaffold_664:2105-3998	control	clone I	22,6981	2,84531	-2,99591	3,48108	0,000499433	0,00872448	yes
TNNC1	scaffold_68:530264-533797	control	clone I	2,48825	74,4447	4,90297	-5,40798	6,37E-08	4,39E-06	yes
ENSGACG00000001127	scaffold_804:3126-5192	control	clone I	0	74,4954	1,80E+308	1,80E+308	4,47E-06	0,000167557	yes
ENSGACG00000000849	scaffold_863:4798-7415	control	clone I	1,56157	14,3288	3,19785	-3,36384	0,000768867	0,0124025	yes
RNPEPL1	groupI:427702-435209	control	clone mix	22,4259	53,9446	1,26631	-3,11276	0,00185349	0,025258	yes
ENSGACG00000004731	groupI:452298-459852	control	clone mix	17,8608	36,1187	1,01595	-3,64062	0,000271984	0,00528624	yes
SUPT5H	groupI:726931-740276	control	clone mix	14,7933	24,2284	0,711749	-4,01424	5,96E-05	0,00149382	yes
PIK3C3	groupI:1835797-1849578	control	clone mix	18,7046	43,1089	1,2076	-3,09235	0,00198578	0,026683	yes
SAMSN1	groupI:3080901-3085572	control	clone mix	13,324	33,5059	1,32175	-3,58072	0,000342645	0,00640868	yes
POLR2I	groupI:4056680-4058563	control	clone mix	95,7701	26,9368	-1,83	4,00505	6,20E-05	0,00154256	yes
SNRPD2	groupI:4066932-4068501	control	clone mix	520,325	165,878	-1,64929	4,17526	2,98E-05	0,000835711	yes
ENSGACG00000006908	groupI:4256609-4274453	control	clone mix	67,2342	8,69163	-2,9515	6,56465	5,22E-11	8,61E-09	yes
CCNL1	groupI:4706528-4713986	control	clone mix	31,4943	14,8537	-1,08429	4,48719	7,22E-06	0,000251578	yes
