# Supplementary material for: Specific Gene Expression Responses to Parasite Genotypes Reveal Redundancy of Innate Immunity in Vertebrates
Source: PLoS One. 2014 Sep 25;9(9):e108001. doi: 10.1371/journal.pone.0108001 (PMC4177871; doi:10.1371/journal.pone.0108001)
Supplement: Table S4 — Putative immune system related genes in three-spined stickleback. Gene names were obtained either via the ensembl biomart filter, querying the Homo sapiens transcriptome for genes belonging to ”immune system process” (GO:0002376), or via a published list containing immune genes identified in cod (Gadus morhua) and pipefish (Syngnathus typhle). (PDF) [file pone.0108001.s004.pdf]

Supplementary table S.4 Putative immune system related genes in three-spined stickleback. Gene names were obtained either via the ensembl biomart filter, querying the Homo sapiens transcriptome for genes belonging to „immune system process“ (GO:0002376), or via a published list containing immune genes identified in cod (*Gadus morhua*) and pipefish (*Syngnathus typhle*).

stickleback_gene_name	human_gene_name	source
ACIN1	ACIN1	ensembl biomart, HSA, GO:0002376
ACTR1B	ACTR1B	ensembl biomart, HSA, GO:0002376
ADA	ADA	ensembl biomart, HSA, GO:0002376
ADAM10	ADAM10	ensembl biomart, HSA, GO:0002376
ADAM17 (1 of 2)	ADAM17	ensembl biomart, HSA, GO:0002376
ADAM17 (2 of 2)	ADAM17	ensembl biomart, HSA, GO:0002376
ADAM8 (1 of 2)	ADAM8	ensembl biomart, HSA, GO:0002376
ADAM8 (2 of 2)	ADAM8	ensembl biomart, HSA, GO:0002376
ADAM9	ADAM9	ensembl biomart, HSA, GO:0002376
ADAMTS13	ADAMTS13	ensembl biomart, HSA, GO:0002376
ADAR	ADAR	ensembl biomart, HSA, GO:0002376
ADORA3 (1 of 2)	ADORA3	ensembl biomart, HSA, GO:0002376
ADORA3 (2 of 2)	ADORA3	ensembl biomart, HSA, GO:0002376
ADSS	ADSS	ensembl biomart, HSA, GO:0002376
ADSSL1	ADSSL1	ensembl biomart, HSA, GO:0002376
AICDA	AICDA	ensembl biomart, HSA, GO:0002376
AIMP1 (1 of 2)	AIMP1	ensembl biomart, HSA, GO:0002376
AIMP1 (2 of 2)	AIMP1	ensembl biomart, HSA, GO:0002376
AK7 (1 of 2)	AK7	ensembl biomart, HSA, GO:0002376
AK7 (2 of 2)	AK7	ensembl biomart, HSA, GO:0002376
AKIRIN2 (1 of 2)	AKIRIN2	ensembl biomart, HSA, GO:0002376
AKIRIN2 (2 of 2)	AKIRIN2	ensembl biomart, HSA, GO:0002376
AKT1	AKT1	ensembl biomart, HSA, GO:0002376
ALAS2	ALAS2	ensembl biomart, HSA, GO:0002376
ANGPT1	ANGPT1	ensembl biomart, HSA, GO:0002376
ANGPT2	ANGPT2	ensembl biomart, HSA, GO:0002376
ANK1 (1 of 2)	ANK1	ensembl biomart, HSA, GO:0002376
ANK1 (2 of 2)	ANK1	ensembl biomart, HSA, GO:0002376
ANXA1 (1 of 2)	ANXA1	ensembl biomart, HSA, GO:0002376
ANXA1 (2 of 2)	ANXA1	ensembl biomart, HSA, GO:0002376
ANXA3 (1 of 2)	ANXA3	ensembl biomart, HSA, GO:0002376
ANXA3 (2 of 2)	ANXA3	ensembl biomart, HSA, GO:0002376
AP1B1	AP1B1	ensembl biomart, HSA, GO:0002376
AP1G1	AP1G1	ensembl biomart, HSA, GO:0002376
AP1M1	AP1M1	ensembl biomart, HSA, GO:0002376
AP1M2	AP1M2	ensembl biomart, HSA, GO:0002376
AP1S1	AP1S1	ensembl biomart, HSA, GO:0002376
AP1S2 (1 of 2)	AP1S2	ensembl biomart, HSA, GO:0002376
AP1S2 (2 of 2)	AP1S2	ensembl biomart, HSA, GO:0002376
AP1S3 (1 of 2)	AP1S3	ensembl biomart, HSA, GO:0002376
AP2A1	AP2A1	ensembl biomart, HSA, GO:0002376
AP2A2	AP2A2	ensembl biomart, HSA, GO:0002376
AP2B1	AP2B1	ensembl biomart, HSA, GO:0002376
AP2M1	AP2M1	ensembl biomart, HSA, GO:0002376
AP2S1	AP2S1	ensembl biomart, HSA, GO:0002376
AP3B1 (1 of 2)	AP3B1	ensembl biomart, HSA, GO:0002376
AP3B1 (2 of 2)	AP3B1	ensembl biomart, HSA, GO:0002376
AP3D1	AP3D1	ensembl biomart, HSA, GO:0002376
APC	APC	ensembl biomart, HSA, GO:0002376
APOB (1 of 5)	APOB	ensembl biomart, HSA, GO:0002376
APOB (2 of 5)	APOB	ensembl biomart, HSA, GO:0002376
APOB (3 of 5)	APOB	ensembl biomart, HSA, GO:0002376
APOB (4 of 5)	APOB	ensembl biomart, HSA, GO:0002376
APOB (5 of 5)	APOB	ensembl biomart, HSA, GO:0002376
APP (1 of 2)	APP	ensembl biomart, HSA, GO:0002376
APP (2 of 2)	APP	ensembl biomart, HSA, GO:0002376
AQP4	AQP4	ensembl biomart, HSA, GO:0002376
AQP9 (1 of 2)	AQP9	ensembl biomart, HSA, GO:0002376
AQP9 (2 of 2)	AQP9	ensembl biomart, HSA, GO:0002376
ARG2	ARG2	ensembl biomart, HSA, GO:0002376
ARHGEF5	ARHGEF5	ensembl biomart, HSA, GO:0002376
ASS1	ASS1	ensembl biomart, HSA, GO:0002376
ATF1	ATF1	ensembl biomart, HSA, GO:0002376
ATF2	ATF2	ensembl biomart, HSA, GO:0002376
ATG12	ATG12	ensembl biomart, HSA, GO:0002376
ATG5	ATG5	ensembl biomart, HSA, GO:0002376
ATG9A	ATG9A	ensembl biomart, HSA, GO:0002376
ATM (1 of 2)	ATM	ensembl biomart, HSA, GO:0002376
ATM (2 of 2)	ATM	ensembl biomart, HSA, GO:0002376
ATP1B1 (1 of 2)	ATP1B1	ensembl biomart, HSA, GO:0002376
ATP1B1 (2 of 2)	ATP1B1	ensembl biomart, HSA, GO:0002376
ATP1B2 (1 of 2)	ATP1B2	ensembl biomart, HSA, GO:0002376
ATP1B2 (2 of 2)	ATP1B2	ensembl biomart, HSA, GO:0002376
ATP1B3	ATP1B3	ensembl biomart, HSA, GO:0002376
ATP6V0A2 (1 of 2)	ATP6V0A2	ensembl biomart, HSA, GO:0002376
ATP6V0A2 (2 of 2)	ATP6V0A2	ensembl biomart, HSA, GO:0002376
ATP7A	ATP7A	ensembl biomart, HSA, GO:0002376
AXL	AXL	ensembl biomart, HSA, GO:0002376
B4GALT1	B4GALT1	ensembl biomart, HSA, GO:0002376
BANK1	BANK1	ensembl biomart, HSA, GO:0002376
BARX1	BARX1	ensembl biomart, HSA, GO:0002376
BAX	BAX	ensembl biomart, HSA, GO:0002376
BCAP31	BCAP31	ensembl biomart, HSA, GO:0002376
BCL10	BCL10	ensembl biomart, HSA, GO:0002376
BCL11A (1 of 2)	BCL11A	ensembl biomart, HSA, GO:0002376
BCL11A (2 of 2)	BCL11A	ensembl biomart, HSA, GO:0002376
BCL2	BCL2	ensembl biomart, HSA, GO:0002376
BCL2L1 (1 of 2)	BCL2L1	ensembl biomart, HSA, GO:0002376
BCL2L1 (2 of 2)	BCL2L1	ensembl biomart, HSA, GO:0002376
BCL6 (1 of 2)	BCL6	ensembl biomart, HSA, GO:0002376
BCL6 (2 of 2)	BCL6	ensembl biomart, HSA, GO:0002376
BDKRB2 (1 of 4)	BDKRB2	ensembl biomart, HSA, GO:0002376
BDKRB2 (2 of 4)	BDKRB2	ensembl biomart, HSA, GO:0002376
BDKRB2 (3 of 4)	BDKRB2	ensembl biomart, HSA, GO:0002376
BDKRB2 (4 of 4)	BDKRB2	ensembl biomart, HSA, GO:0002376
BECN1	BECN1	ensembl biomart, HSA, GO:0002376
BLM	BLM	ensembl biomart, HSA, GO:0002376
BLMH	BLMH	ensembl biomart, HSA, GO:0002376
BLNK	BLNK	ensembl biomart, HSA, GO:0002376
BMI1	BMI1	ensembl biomart, HSA, GO:0002376
BMP4	BMP4	ensembl biomart, HSA, GO:0002376
BMP6	BMP6	ensembl biomart, HSA, GO:0002376
BMPR1A	BMPR1A	ensembl biomart, HSA, GO:0002376
BNIP3	BNIP3	ensembl biomart, HSA, GO:0002376
BNIP3L (1 of 2)	BNIP3L	ensembl biomart, HSA, GO:0002376
BNIP3L (2 of 2)	BNIP3L	ensembl biomart, HSA, GO:0002376
BPGM	BPGM	ensembl biomart, HSA, GO:0002376
BRCA2	BRCA2	ensembl biomart, HSA, GO:0002376
BTK	BTK	ensembl biomart, HSA, GO:0002376
C11orf82	C11orf82	ensembl biomart, HSA, GO:0002376
C1QBP	C1QBP	ensembl biomart, HSA, GO:0002376
C1QC	C1QC	ensembl biomart, HSA, GO:0002376
C3 (1 of 8)	C3	ensembl biomart, HSA, GO:0002376
C3 (2 of 8)	C3	ensembl biomart, HSA, GO:0002376
C3 (3 of 8)	C3	ensembl biomart, HSA, GO:0002376
C3 (4 of 8)	C3	ensembl biomart, HSA, GO:0002376
C3 (5 of 8)	C3	ensembl biomart, HSA, GO:0002376
C3 (6 of 8)	C3	ensembl biomart, HSA, GO:0002376
C3 (7 of 8)	C3	ensembl biomart, HSA, GO:0002376
C3 (8 of 8)	C3	ensembl biomart, HSA, GO:0002376
C5AR1	C5AR1	ensembl biomart, HSA, GO:0002376
C6	C6	ensembl biomart, HSA, GO:0002376
C7 (1 of 2)	C7	ensembl biomart, HSA, GO:0002376
C7 (2 of 2)	C7	ensembl biomart, HSA, GO:0002376
C8A	C8A	ensembl biomart, HSA, GO:0002376
C8B	C8B	ensembl biomart, HSA, GO:0002376
C8G	C8G	ensembl biomart, HSA, GO:0002376
C9	C9	ensembl biomart, HSA, GO:0002376
CACNB4 (1 of 2)	CACNB4	ensembl biomart, HSA, GO:0002376
CACNB4 (2 of 2)	CACNB4	ensembl biomart, HSA, GO:0002376
CALCA	CALCA	ensembl biomart, HSA, GO:0002376
CALCR	CALCR	ensembl biomart, HSA, GO:0002376
CAMK2A	CAMK2A	ensembl biomart, HSA, GO:0002376
CAMK4	CAMK4	ensembl biomart, HSA, GO:0002376
CANX	CANX	ensembl biomart, HSA, GO:0002376
CARD11	CARD11	ensembl biomart, HSA, GO:0002376
CARD9	CARD9	ensembl biomart, HSA, GO:0002376
CASP3 (1 of 4)	CASP3	ensembl biomart, HSA, GO:0002376
CASP3 (2 of 4)	CASP3	ensembl biomart, HSA, GO:0002376
CASP3 (3 of 4)	CASP3	ensembl biomart, HSA, GO:0002376
CASP3 (4 of 4)	CASP3	ensembl biomart, HSA, GO:0002376
CASP8	CASP8	ensembl biomart, HSA, GO:0002376
CAV1	CAV1	ensembl biomart, HSA, GO:0002376
CBFA2T3	CBFA2T3	ensembl biomart, HSA, GO:0002376
CBFB	CBFB	ensembl biomart, HSA, GO:0002376
CBLB	CBLB	ensembl biomart, HSA, GO:0002376
CCNB2 (1 of 2)	CCNB2	ensembl biomart, HSA, GO:0002376
CCNB2 (2 of 2)	CCNB2	ensembl biomart, HSA, GO:0002376
CCR6 (1 of 2)	CCR6	ensembl biomart, HSA, GO:0002376
CCR6 (2 of 2)	CCR6	ensembl biomart, HSA, GO:0002376
CCR9 (1 of 2)	CCR9	ensembl biomart, HSA, GO:0002376
CCR9 (2 of 2)	CCR9	ensembl biomart, HSA, GO:0002376
CCRL1 (1 of 2)	CCRL1	ensembl biomart, HSA, GO:0002376
CCRL1 (2 of 2)	CCRL1	ensembl biomart, HSA, GO:0002376
CD164	CD164	ensembl biomart, HSA, GO:0002376
CD276	CD276	ensembl biomart, HSA, GO:0002376
CD74 (1 of 2)	CD74	ensembl biomart, HSA, GO:0002376
CD93	CD93	ensembl biomart, HSA, GO:0002376
CD97 (1 of 2)	CD97	ensembl biomart, HSA, GO:0002376
CD97 (2 of 2)	CD97	ensembl biomart, HSA, GO:0002376
CDK1	CDK1	ensembl biomart, HSA, GO:0002376
CDK13	CDK13	ensembl biomart, HSA, GO:0002376
CDK6	CDK6	ensembl biomart, HSA, GO:0002376
CENPE	CENPE	ensembl biomart, HSA, GO:0002376
CFB	CFB	ensembl biomart, HSA, GO:0002376
CFI	CFI	ensembl biomart, HSA, GO:0002376
CFP	CFP	ensembl biomart, HSA, GO:0002376
CHD7	CHD7	ensembl biomart, HSA, GO:0002376
CHIA	CHIA	ensembl biomart, HSA, GO:0002376
CHID1	CHID1	ensembl biomart, HSA, GO:0002376
CHRNA4	CHRNA4	ensembl biomart, HSA, GO:0002376
CHRNB2	CHRNB2	ensembl biomart, HSA, GO:0002376
CHST3 (1 of 2)	CHST3	ensembl biomart, HSA, GO:0002376
CHST3 (2 of 2)	CHST3	ensembl biomart, HSA, GO:0002376
CHUK (1 of 2)	CHUK	ensembl biomart, HSA, GO:0002376
CHUK (2 of 2)	CHUK	ensembl biomart, HSA, GO:0002376
CIAPIN1	CIAPIN1	ensembl biomart, HSA, GO:0002376
CLEC4M	CLEC4M	ensembl biomart, HSA, GO:0002376
CLNK	CLNK	ensembl biomart, HSA, GO:0002376
CLTA	CLTA	ensembl biomart, HSA, GO:0002376
CLU	CLU	ensembl biomart, HSA, GO:0002376
CMKLR1 (1 of 2)	CMKLR1	ensembl biomart, HSA, GO:0002376
CMKLR1 (2 of 2)	CMKLR1	ensembl biomart, HSA, GO:0002376
CNN2	CNN2	ensembl biomart, HSA, GO:0002376
COL1A1 (1 of 2)	COL1A1	ensembl biomart, HSA, GO:0002376
COL1A1 (2 of 2)	COL1A1	ensembl biomart, HSA, GO:0002376
COL1A2	COL1A2	ensembl biomart, HSA, GO:0002376
COL4A3BP (1 of 2)	COL4A3BP	ensembl biomart, HSA, GO:0002376
COL4A3BP (2 of 2)	COL4A3BP	ensembl biomart, HSA, GO:0002376
COLEC12 (1 of 2)	COLEC12	ensembl biomart, HSA, GO:0002376
COLEC12 (2 of 2)	COLEC12	ensembl biomart, HSA, GO:0002376
CORO1A	CORO1A	ensembl biomart, HSA, GO:0002376
CPLX2	CPLX2	ensembl biomart, HSA, GO:0002376
CRCP	CRCP	ensembl biomart, HSA, GO:0002376
CREB1 (1 of 2)	CREB1	ensembl biomart, HSA, GO:0002376
CREB1 (2 of 2)	CREB1	ensembl biomart, HSA, GO:0002376
CREBBP (1 of 2)	CREBBP	ensembl biomart, HSA, GO:0002376
CREBBP (2 of 2)	CREBBP	ensembl biomart, HSA, GO:0002376
CRHR1	CRHR1	ensembl biomart, HSA, GO:0002376
CRIP2 (1 of 2)	CRIP2	ensembl biomart, HSA, GO:0002376
CRIP2 (2 of 2)	CRIP2	ensembl biomart, HSA, GO:0002376
CRISP3	CRISP3	ensembl biomart, HSA, GO:0002376
CRKL	CRKL	ensembl biomart, HSA, GO:0002376
CSF1R (1 of 2)	CSF1R	ensembl biomart, HSA, GO:0002376
CSF1R (2 of 2)	CSF1R	ensembl biomart, HSA, GO:0002376
CSK (1 of 2)	CSK	ensembl biomart, HSA, GO:0002376
CSK (2 of 2)	CSK	ensembl biomart, HSA, GO:0002376
CTNNBL1	CTNNBL1	ensembl biomart, HSA, GO:0002376
CTSB	CTSB	ensembl biomart, HSA, GO:0002376
CTSC	CTSC	ensembl biomart, HSA, GO:0002376
CTSD	CTSD	ensembl biomart, HSA, GO:0002376
CTSE	CTSE	ensembl biomart, HSA, GO:0002376
CTSF	CTSF	ensembl biomart, HSA, GO:0002376
CTSH	CTSH	ensembl biomart, HSA, GO:0002376
CTSK	CTSK	ensembl biomart, HSA, GO:0002376
CTSS (1 of 2)	CTSS	ensembl biomart, HSA, GO:0002376
CTSS (2 of 2)	CTSS	ensembl biomart, HSA, GO:0002376
CXADR	CXADR	ensembl biomart, HSA, GO:0002376
CXCL12 (1 of 2)	CXCL12	ensembl biomart, HSA, GO:0002376
CXCL12 (2 of 2)	CXCL12	ensembl biomart, HSA, GO:0002376
CXCR4 (1 of 2)	CXCR4	ensembl biomart, HSA, GO:0002376
CXCR4 (2 of 2)	CXCR4	ensembl biomart, HSA, GO:0002376
CYBA	CYBA	ensembl biomart, HSA, GO:0002376
CYBB	CYBB	ensembl biomart, HSA, GO:0002376
CYFIP2	CYFIP2	ensembl biomart, HSA, GO:0002376
CYP19A1 (1 of 2)	CYP19A1	ensembl biomart, HSA, GO:0002376
CYP19A1 (2 of 2)	CYP19A1	ensembl biomart, HSA, GO:0002376
CYP27B1	CYP27B1	ensembl biomart, HSA, GO:0002376
CYSLTR2	CYSLTR2	ensembl biomart, HSA, GO:0002376
DAB2	DAB2	ensembl biomart, HSA, GO:0002376
DCLRE1C	DCLRE1C	ensembl biomart, HSA, GO:0002376
DCTN1 (1 of 2)	DCTN1	ensembl biomart, HSA, GO:0002376
DCTN1 (2 of 2)	DCTN1	ensembl biomart, HSA, GO:0002376
DCTN6 (1 of 2)	DCTN6	ensembl biomart, HSA, GO:0002376
DCTN6 (2 of 2)	DCTN6	ensembl biomart, HSA, GO:0002376
DDOST	DDOST	ensembl biomart, HSA, GO:0002376
DICER1	DICER1	ensembl biomart, HSA, GO:0002376
DNAJA3 (1 of 2)	DNAJA3	ensembl biomart, HSA, GO:0002376
DNAJA3 (2 of 2)	DNAJA3	ensembl biomart, HSA, GO:0002376
DNASE2	DNASE2	ensembl biomart, HSA, GO:0002376
DNM2 (1 of 2)	DNM2	ensembl biomart, HSA, GO:0002376
DNM2 (2 of 2)	DNM2	ensembl biomart, HSA, GO:0002376
DOCK2	DOCK2	ensembl biomart, HSA, GO:0002376
DOK2	DOK2	ensembl biomart, HSA, GO:0002376
DUSP3	DUSP3	ensembl biomart, HSA, GO:0002376
DUSP4	DUSP4	ensembl biomart, HSA, GO:0002376
DUSP6	DUSP6	ensembl biomart, HSA, GO:0002376
DUSP7 (1 of 2)	DUSP7	ensembl biomart, HSA, GO:0002376
DYNC1H1	DYNC1H1	ensembl biomart, HSA, GO:0002376
DYNC1I1	DYNC1I1	ensembl biomart, HSA, GO:0002376
DYNC1I2 (1 of 2)	DYNC1I2	ensembl biomart, HSA, GO:0002376
DYNC1I2 (2 of 2)	DYNC1I2	ensembl biomart, HSA, GO:0002376
DYNC1LI2	DYNC1LI2	ensembl biomart, HSA, GO:0002376
DYNC2H1	DYNC2H1	ensembl biomart, HSA, GO:0002376
DYNC2LI1	DYNC2LI1	ensembl biomart, HSA, GO:0002376
DYNLL2	DYNLL2	ensembl biomart, HSA, GO:0002376
EBI3	EBI3	ensembl biomart, HSA, GO:0002376
EDA	EDA	ensembl biomart, HSA, GO:0002376
EDN2	EDN2	ensembl biomart, HSA, GO:0002376
EDNRB (1 of 2)	EDNRB	ensembl biomart, HSA, GO:0002376
EDNRB (2 of 2)	EDNRB	ensembl biomart, HSA, GO:0002376
EFNA2	EFNA2	ensembl biomart, HSA, GO:0002376
EGR1	EGR1	ensembl biomart, HSA, GO:0002376
EIF2AK2 (1 of 2)	EIF2AK2	ensembl biomart, HSA, GO:0002376
EIF2AK2 (2 of 2)	EIF2AK2	ensembl biomart, HSA, GO:0002376
ELK1	ELK1	ensembl biomart, HSA, GO:0002376
ELMOD2	ELMOD2	ensembl biomart, HSA, GO:0002376
EMR2	EMR2	ensembl biomart, HSA, GO:0002376
ENDOU	ENDOU	ensembl biomart, HSA, GO:0002376
ENPP1	ENPP1	ensembl biomart, HSA, GO:0002376
ENPP2 (1 of 2)	ENPP2	ensembl biomart, HSA, GO:0002376
ENPP2 (2 of 2)	ENPP2	ensembl biomart, HSA, GO:0002376
EOMES (1 of 2)	EOMES	ensembl biomart, HSA, GO:0002376
EOMES (2 of 2)	EOMES	ensembl biomart, HSA, GO:0002376
EP300 (1 of 2)	EP300	ensembl biomart, HSA, GO:0002376
EP300 (2 of 2)	EP300	ensembl biomart, HSA, GO:0002376
EPAS1	EPAS1	ensembl biomart, HSA, GO:0002376
EPHA2 (1 of 2)	EPHA2	ensembl biomart, HSA, GO:0002376
EPHA2 (2 of 2)	EPHA2	ensembl biomart, HSA, GO:0002376
EPHB3 (1 of 2)	EPHB3	ensembl biomart, HSA, GO:0002376
EPHB3 (2 of 2)	EPHB3	ensembl biomart, HSA, GO:0002376
EPO	EPO	ensembl biomart, HSA, GO:0002376
ERAP1	ERAP1	ensembl biomart, HSA, GO:0002376
ERAP2	ERAP2	ensembl biomart, HSA, GO:0002376
ERCC1	ERCC1	ensembl biomart, HSA, GO:0002376
ERCC2	ERCC2	ensembl biomart, HSA, GO:0002376
ESAM	ESAM	ensembl biomart, HSA, GO:0002376
ETS1	ETS1	ensembl biomart, HSA, GO:0002376
EXO1	EXO1	ensembl biomart, HSA, GO:0002376
EXOC6	EXOC6	ensembl biomart, HSA, GO:0002376
EXOSC3	EXOSC3	ensembl biomart, HSA, GO:0002376
EXOSC4	EXOSC4	ensembl biomart, HSA, GO:0002376
EXOSC5	EXOSC5	ensembl biomart, HSA, GO:0002376
EXOSC6	EXOSC6	ensembl biomart, HSA, GO:0002376
EXOSC9	EXOSC9	ensembl biomart, HSA, GO:0002376
F11R	F11R	ensembl biomart, HSA, GO:0002376
FAM20C	FAM20C	ensembl biomart, HSA, GO:0002376
FAM65C	FAM65C	ensembl biomart, HSA, GO:0002376
FANCC	FANCC	ensembl biomart, HSA, GO:0002376
FECH	FECH	ensembl biomart, HSA, GO:0002376
FGF10 (1 of 2)	FGF10	ensembl biomart, HSA, GO:0002376
FGF10 (2 of 2)	FGF10	ensembl biomart, HSA, GO:0002376
FGF3	FGF3	ensembl biomart, HSA, GO:0002376
FGFR2	FGFR2	ensembl biomart, HSA, GO:0002376
FKBP1B	FKBP1B	ensembl biomart, HSA, GO:0002376
FLVCR1	FLVCR1	ensembl biomart, HSA, GO:0002376
FN1 (1 of 2)	FN1	ensembl biomart, HSA, GO:0002376
FN1 (2 of 2)	FN1	ensembl biomart, HSA, GO:0002376
FOS (1 of 2)	FOS	ensembl biomart, HSA, GO:0002376
FOS (2 of 2)	FOS	ensembl biomart, HSA, GO:0002376
FOXJ1	FOXJ1	ensembl biomart, HSA, GO:0002376
FOXN1	FOXN1	ensembl biomart, HSA, GO:0002376
FOXP1	FOXP1	ensembl biomart, HSA, GO:0002376
FST	FST	ensembl biomart, HSA, GO:0002376
FYB	FYB	ensembl biomart, HSA, GO:0002376
FYN	FYN	ensembl biomart, HSA, GO:0002376
FZD5	FZD5	ensembl biomart, HSA, GO:0002376
FZD7	FZD7	ensembl biomart, HSA, GO:0002376
FZD8	FZD8	ensembl biomart, HSA, GO:0002376
FZD9	FZD9	ensembl biomart, HSA, GO:0002376
G6PD (1 of 2)	G6PD	ensembl biomart, HSA, GO:0002376
G6PD (2 of 2)	G6PD	ensembl biomart, HSA, GO:0002376
GAB2	GAB2	ensembl biomart, HSA, GO:0002376
GAB3	GAB3	ensembl biomart, HSA, GO:0002376
GALNT2	GALNT2	ensembl biomart, HSA, GO:0002376
GAS6	GAS6	ensembl biomart, HSA, GO:0002376
GATA2 (1 of 2)	GATA2	ensembl biomart, HSA, GO:0002376
GATA2 (2 of 2)	GATA2	ensembl biomart, HSA, GO:0002376
GCH1	GCH1	ensembl biomart, HSA, GO:0002376
GCNT3 (1 of 2)	GCNT3	ensembl biomart, HSA, GO:0002376
GCNT3 (2 of 2)	GCNT3	ensembl biomart, HSA, GO:0002376
GEM	GEM	ensembl biomart, HSA, GO:0002376
GLI3	GLI3	ensembl biomart, HSA, GO:0002376
GLRX5	GLRX5	ensembl biomart, HSA, GO:0002376
GNL1	GNL1	ensembl biomart, HSA, GO:0002376
GPAM	GPAM	ensembl biomart, HSA, GO:0002376
GPC3	GPC3	ensembl biomart, HSA, GO:0002376
GPR183	GPR183	ensembl biomart, HSA, GO:0002376
GPR65	GPR65	ensembl biomart, HSA, GO:0002376
GRAP2	GRAP2	ensembl biomart, HSA, GO:0002376
GRB14	GRB14	ensembl biomart, HSA, GO:0002376
GRB2	GRB2	ensembl biomart, HSA, GO:0002376
GRB7	GRB7	ensembl biomart, HSA, GO:0002376
GTPBP1	GTPBP1	ensembl biomart, HSA, GO:0002376
HAND2	HAND2	ensembl biomart, HSA, GO:0002376
HCK	HCK	ensembl biomart, HSA, GO:0002376
HCLS1	HCLS1	ensembl biomart, HSA, GO:0002376
HDAC4 (1 of 2)	HDAC4	ensembl biomart, HSA, GO:0002376
HDAC4 (2 of 2)	HDAC4	ensembl biomart, HSA, GO:0002376
HDAC5	HDAC5	ensembl biomart, HSA, GO:0002376
HDAC9	HDAC9	ensembl biomart, HSA, GO:0002376
HELLS	HELLS	ensembl biomart, HSA, GO:0002376
HHEX	HHEX	ensembl biomart, HSA, GO:0002376
HIF1A	HIF1A	ensembl biomart, HSA, GO:0002376
HIPK1 (1 of 2)	HIPK1	ensembl biomart, HSA, GO:0002376
HIPK1 (2 of 2)	HIPK1	ensembl biomart, HSA, GO:0002376
HIPK2	HIPK2	ensembl biomart, HSA, GO:0002376
HMGB1 (1 of 2)	HMGB1	ensembl biomart, HSA, GO:0002376
HMGB1 (2 of 2)	HMGB1	ensembl biomart, HSA, GO:0002376
HMGB2	HMGB2	ensembl biomart, HSA, GO:0002376
HOXA9	HOXA9	ensembl biomart, HSA, GO:0002376
HOXB4	HOXB4	ensembl biomart, HSA, GO:0002376
HPRT1	HPRT1	ensembl biomart, HSA, GO:0002376
HRAS	HRAS	ensembl biomart, HSA, GO:0002376
HRH2 (1 of 2)	HRH2	ensembl biomart, HSA, GO:0002376
HRH2 (2 of 2)	HRH2	ensembl biomart, HSA, GO:0002376
HYAL2 (1 of 2)	HYAL2	ensembl biomart, HSA, GO:0002376
HYAL2 (2 of 2)	HYAL2	ensembl biomart, HSA, GO:0002376
ID2	ID2	ensembl biomart, HSA, GO:0002376
IFI35	IFI35	ensembl biomart, HSA, GO:0002376
IFIH1	IFIH1	ensembl biomart, HSA, GO:0002376
IGF1R (1 of 2)	IGF1R	ensembl biomart, HSA, GO:0002376
IGF1R (2 of 2)	IGF1R	ensembl biomart, HSA, GO:0002376
IK	IK	ensembl biomart, HSA, GO:0002376
IKBKAP	IKBKAP	ensembl biomart, HSA, GO:0002376
IKBKB	IKBKB	ensembl biomart, HSA, GO:0002376
IKBKE	IKBKE	ensembl biomart, HSA, GO:0002376
IKBKG	IKBKG	ensembl biomart, HSA, GO:0002376
IL10RB	IL10RB	ensembl biomart, HSA, GO:0002376
IL12B (3 of 3)	IL12B	ensembl biomart, HSA, GO:0002376
IL16	IL16	ensembl biomart, HSA, GO:0002376
IL1RAP	IL1RAP	ensembl biomart, HSA, GO:0002376
IL1RAPL1 (1 of 2)	IL1RAPL1	ensembl biomart, HSA, GO:0002376
IL1RAPL1 (2 of 2)	IL1RAPL1	ensembl biomart, HSA, GO:0002376
IL1RAPL2	IL1RAPL2	ensembl biomart, HSA, GO:0002376
IL1RL2	IL1RL2	ensembl biomart, HSA, GO:0002376
IL20RB	IL20RB	ensembl biomart, HSA, GO:0002376
IL2RG	IL2RG	ensembl biomart, HSA, GO:0002376
IL7R	IL7R	ensembl biomart, HSA, GO:0002376
ILF2	ILF2	ensembl biomart, HSA, GO:0002376
IMPDH1 (1 of 2)	IMPDH1	ensembl biomart, HSA, GO:0002376
INHA	INHA	ensembl biomart, HSA, GO:0002376
INHBA (1 of 2)	INHBA	ensembl biomart, HSA, GO:0002376
INHBA (2 of 2)	INHBA	ensembl biomart, HSA, GO:0002376
INPP5D	INPP5D	ensembl biomart, HSA, GO:0002376
INS	INS	ensembl biomart, HSA, GO:0002376
IP6K2 (1 of 2)	IP6K2	ensembl biomart, HSA, GO:0002376
IP6K2 (2 of 2)	IP6K2	ensembl biomart, HSA, GO:0002376
IRAK1	IRAK1	ensembl biomart, HSA, GO:0002376
IRAK3	IRAK3	ensembl biomart, HSA, GO:0002376
IRAK4	IRAK4	ensembl biomart, HSA, GO:0002376
IREB2	IREB2	ensembl biomart, HSA, GO:0002376
IRF2	IRF2	ensembl biomart, HSA, GO:0002376
IRF3	IRF3	ensembl biomart, HSA, GO:0002376
IRF4 (1 of 2)	IRF4	ensembl biomart, HSA, GO:0002376
IRF4 (2 of 2)	IRF4	ensembl biomart, HSA, GO:0002376
IRF5	IRF5	ensembl biomart, HSA, GO:0002376
IRF6	IRF6	ensembl biomart, HSA, GO:0002376
IRF7	IRF7	ensembl biomart, HSA, GO:0002376
IRF8	IRF8	ensembl biomart, HSA, GO:0002376
ITCH (1 of 2)	ITCH	ensembl biomart, HSA, GO:0002376
ITCH (2 of 2)	ITCH	ensembl biomart, HSA, GO:0002376
ITGA1	ITGA1	ensembl biomart, HSA, GO:0002376
ITGA5 (1 of 2)	ITGA5	ensembl biomart, HSA, GO:0002376
ITGA5 (2 of 2)	ITGA5	ensembl biomart, HSA, GO:0002376
ITGA6 (1 of 2)	ITGA6	ensembl biomart, HSA, GO:0002376
ITGA6 (2 of 2)	ITGA6	ensembl biomart, HSA, GO:0002376
ITGA9	ITGA9	ensembl biomart, HSA, GO:0002376
ITGAV	ITGAV	ensembl biomart, HSA, GO:0002376
ITGB1 (1 of 2)	ITGB1	ensembl biomart, HSA, GO:0002376
ITGB1 (2 of 2)	ITGB1	ensembl biomart, HSA, GO:0002376
ITGB5	ITGB5	ensembl biomart, HSA, GO:0002376
ITK	ITK	ensembl biomart, HSA, GO:0002376
ITPKB	ITPKB	ensembl biomart, HSA, GO:0002376
JAG1 (1 of 2)	JAG1	ensembl biomart, HSA, GO:0002376
JAG1 (2 of 2)	JAG1	ensembl biomart, HSA, GO:0002376
JAG2	JAG2	ensembl biomart, HSA, GO:0002376
JAK1	JAK1	ensembl biomart, HSA, GO:0002376
JAK2 (1 of 2)	JAK2	ensembl biomart, HSA, GO:0002376
JAK2 (2 of 2)	JAK2	ensembl biomart, HSA, GO:0002376
JAM2 (1 of 2)	JAM2	ensembl biomart, HSA, GO:0002376
JAM2 (2 of 2)	JAM2	ensembl biomart, HSA, GO:0002376
JAM3 (1 of 2)	JAM3	ensembl biomart, HSA, GO:0002376
JAM3 (2 of 2)	JAM3	ensembl biomart, HSA, GO:0002376
JARID2	JARID2	ensembl biomart, HSA, GO:0002376
JMJD6	JMJD6	ensembl biomart, HSA, GO:0002376
JUN (1 of 2)	JUN	ensembl biomart, HSA, GO:0002376
JUN (2 of 2)	JUN	ensembl biomart, HSA, GO:0002376
JUNB (1 of 2)	JUNB	ensembl biomart, HSA, GO:0002376
JUNB (2 of 2)	JUNB	ensembl biomart, HSA, GO:0002376
KCNJ8	KCNJ8	ensembl biomart, HSA, GO:0002376
KIF11	KIF11	ensembl biomart, HSA, GO:0002376
KIF15	KIF15	ensembl biomart, HSA, GO:0002376
KIF18A	KIF18A	ensembl biomart, HSA, GO:0002376
KIF22	KIF22	ensembl biomart, HSA, GO:0002376
KIF23 (1 of 2)	KIF23	ensembl biomart, HSA, GO:0002376
KIF23 (2 of 2)	KIF23	ensembl biomart, HSA, GO:0002376
KIF26A (1 of 2)	KIF26A	ensembl biomart, HSA, GO:0002376
KIF26A (2 of 2)	KIF26A	ensembl biomart, HSA, GO:0002376
KIF2C	KIF2C	ensembl biomart, HSA, GO:0002376
KIF3A	KIF3A	ensembl biomart, HSA, GO:0002376
KIF3B	KIF3B	ensembl biomart, HSA, GO:0002376
KIF3C (1 of 2)	KIF3C	ensembl biomart, HSA, GO:0002376
KIF3C (2 of 2)	KIF3C	ensembl biomart, HSA, GO:0002376
KIF4A	KIF4A	ensembl biomart, HSA, GO:0002376
KIF5A (1 of 2)	KIF5A	ensembl biomart, HSA, GO:0002376
KIF5A (2 of 2)	KIF5A	ensembl biomart, HSA, GO:0002376
KIFAP3 (1 of 2)	KIFAP3	ensembl biomart, HSA, GO:0002376
KIFAP3 (2 of 2)	KIFAP3	ensembl biomart, HSA, GO:0002376
KIT	KIT	ensembl biomart, HSA, GO:0002376
KLC1	KLC1	ensembl biomart, HSA, GO:0002376
KLC2	KLC2	ensembl biomart, HSA, GO:0002376
KLF2 (1 of 2)	KLF2	ensembl biomart, HSA, GO:0002376
KLF2 (2 of 2)	KLF2	ensembl biomart, HSA, GO:0002376
KLF6 (1 of 2)	KLF6	ensembl biomart, HSA, GO:0002376
KLF6 (2 of 2)	KLF6	ensembl biomart, HSA, GO:0002376
KRAS	KRAS	ensembl biomart, HSA, GO:0002376
KYNU	KYNU	ensembl biomart, HSA, GO:0002376
L1CAM	L1CAM	ensembl biomart, HSA, GO:0002376
LCK	LCK	ensembl biomart, HSA, GO:0002376
LEF1	LEF1	ensembl biomart, HSA, GO:0002376
LGALS1 (1 of 3)	LGALS1	ensembl biomart, HSA, GO:0002376
LGALS1 (2 of 3)	LGALS1	ensembl biomart, HSA, GO:0002376
LGALS1 (3 of 3)	LGALS1	ensembl biomart, HSA, GO:0002376
LGALS3	LGALS3	ensembl biomart, HSA, GO:0002376
LGALS8 (1 of 2)	LGALS8	ensembl biomart, HSA, GO:0002376
LGALS8 (2 of 2)	LGALS8	ensembl biomart, HSA, GO:0002376
LGMN	LGMN	ensembl biomart, HSA, GO:0002376
LIG4	LIG4	ensembl biomart, HSA, GO:0002376
LMO4	LMO4	ensembl biomart, HSA, GO:0002376
LNPEP	LNPEP	ensembl biomart, HSA, GO:0002376
LRRC17	LRRC17	ensembl biomart, HSA, GO:0002376
LRRC8A (1 of 2)	LRRC8A	ensembl biomart, HSA, GO:0002376
LRRC8A (2 of 2)	LRRC8A	ensembl biomart, HSA, GO:0002376
LYL1	LYL1	ensembl biomart, HSA, GO:0002376
LYN	LYN	ensembl biomart, HSA, GO:0002376
LYST	LYST	ensembl biomart, HSA, GO:0002376
MAEA	MAEA	ensembl biomart, HSA, GO:0002376
MAG	MAG	ensembl biomart, HSA, GO:0002376
MALT1	MALT1	ensembl biomart, HSA, GO:0002376
MAP2K1	MAP2K1	ensembl biomart, HSA, GO:0002376
MAP2K2 (1 of 2)	MAP2K2	ensembl biomart, HSA, GO:0002376
MAP2K2 (2 of 2)	MAP2K2	ensembl biomart, HSA, GO:0002376
MAP2K4	MAP2K4	ensembl biomart, HSA, GO:0002376
MAP2K6	MAP2K6	ensembl biomart, HSA, GO:0002376
MAP2K7	MAP2K7	ensembl biomart, HSA, GO:0002376
MAP3K7	MAP3K7	ensembl biomart, HSA, GO:0002376
MAP3K8	MAP3K8	ensembl biomart, HSA, GO:0002376
MAP4K2	MAP4K2	ensembl biomart, HSA, GO:0002376
MAPK1	MAPK1	ensembl biomart, HSA, GO:0002376
MAPK10	MAPK10	ensembl biomart, HSA, GO:0002376
MAPK11	MAPK11	ensembl biomart, HSA, GO:0002376
MAPK14 (1 of 2)	MAPK14	ensembl biomart, HSA, GO:0002376
MAPK14 (2 of 2)	MAPK14	ensembl biomart, HSA, GO:0002376
MAPK7	MAPK7	ensembl biomart, HSA, GO:0002376
MAPK8 (1 of 2)	MAPK8	ensembl biomart, HSA, GO:0002376
MAPK8 (2 of 2)	MAPK8	ensembl biomart, HSA, GO:0002376
MAPK9	MAPK9	ensembl biomart, HSA, GO:0002376
MAPKAP1	MAPKAP1	ensembl biomart, HSA, GO:0002376
MAPKAPK2	MAPKAPK2	ensembl biomart, HSA, GO:0002376
MARCO	MARCO	ensembl biomart, HSA, GO:0002376
MASP1 (1 of 2)	MASP1	ensembl biomart, HSA, GO:0002376
MASP1 (2 of 2)	MASP1	ensembl biomart, HSA, GO:0002376
MAVS	MAVS	ensembl biomart, HSA, GO:0002376
MBP	MBP	ensembl biomart, HSA, GO:0002376
MECOM	MECOM	ensembl biomart, HSA, GO:0002376
MED1	MED1	ensembl biomart, HSA, GO:0002376
MEF2A	MEF2A	ensembl biomart, HSA, GO:0002376
MEF2C (1 of 2)	MEF2C	ensembl biomart, HSA, GO:0002376
MEF2C (2 of 2)	MEF2C	ensembl biomart, HSA, GO:0002376
MELK	MELK	ensembl biomart, HSA, GO:0002376
MEN1	MEN1	ensembl biomart, HSA, GO:0002376
MERTK	MERTK	ensembl biomart, HSA, GO:0002376
MFAP2	MFAP2	ensembl biomart, HSA, GO:0002376
MINK1	MINK1	ensembl biomart, HSA, GO:0002376
MITF (1 of 2)	MITF	ensembl biomart, HSA, GO:0002376
MITF (2 of 2)	MITF	ensembl biomart, HSA, GO:0002376
MIXL1	MIXL1	ensembl biomart, HSA, GO:0002376
MKNK2	MKNK2	ensembl biomart, HSA, GO:0002376
MLF1	MLF1	ensembl biomart, HSA, GO:0002376
MLH1	MLH1	ensembl biomart, HSA, GO:0002376
MLL	MLL	ensembl biomart, HSA, GO:0002376
MLL5	MLL5	ensembl biomart, HSA, GO:0002376
MPZL2 (1 of 2)	MPZL2	ensembl biomart, HSA, GO:0002376
MPZL2 (2 of 2)	MPZL2	ensembl biomart, HSA, GO:0002376
MSH2	MSH2	ensembl biomart, HSA, GO:0002376
MSN (1 of 2)	MSN	ensembl biomart, HSA, GO:0002376
MSN (2 of 2)	MSN	ensembl biomart, HSA, GO:0002376
MST1R (1 of 2)	MST1R	ensembl biomart, HSA, GO:0002376
MST1R (2 of 2)	MST1R	ensembl biomart, HSA, GO:0002376
MYB	MYB	ensembl biomart, HSA, GO:0002376
MYD88	MYD88	ensembl biomart, HSA, GO:0002376
MYH9 (1 of 2)	MYH9	ensembl biomart, HSA, GO:0002376
MYH9 (2 of 2)	MYH9	ensembl biomart, HSA, GO:0002376
MYLPF (1 of 2)	MYLPF	ensembl biomart, HSA, GO:0002376
MYLPF (2 of 2)	MYLPF	ensembl biomart, HSA, GO:0002376
MYO1E	MYO1E	ensembl biomart, HSA, GO:0002376
MYO1F	MYO1F	ensembl biomart, HSA, GO:0002376
MYO9B (1 of 2)	MYO9B	ensembl biomart, HSA, GO:0002376
MYO9B (2 of 2)	MYO9B	ensembl biomart, HSA, GO:0002376
NBEAL2	NBEAL2	ensembl biomart, HSA, GO:0002376
NBN	NBN	ensembl biomart, HSA, GO:0002376
NCAM1 (1 of 2)	NCAM1	ensembl biomart, HSA, GO:0002376
NCAM1 (2 of 2)	NCAM1	ensembl biomart, HSA, GO:0002376
NCF1	NCF1	ensembl biomart, HSA, GO:0002376
NCF2	NCF2	ensembl biomart, HSA, GO:0002376
NCK1	NCK1	ensembl biomart, HSA, GO:0002376
NCK2	NCK2	ensembl biomart, HSA, GO:0002376
NCKAP1L	NCKAP1L	ensembl biomart, HSA, GO:0002376
NCOA6	NCOA6	ensembl biomart, HSA, GO:0002376
NCSTN	NCSTN	ensembl biomart, HSA, GO:0002376
NDRG1 (1 of 2)	NDRG1	ensembl biomart, HSA, GO:0002376
NDRG1 (2 of 2)	NDRG1	ensembl biomart, HSA, GO:0002376
NEDD4	NEDD4	ensembl biomart, HSA, GO:0002376
NFATC2	NFATC2	ensembl biomart, HSA, GO:0002376
NFKB1	NFKB1	ensembl biomart, HSA, GO:0002376
NFKB2	NFKB2	ensembl biomart, HSA, GO:0002376
NFKBIA (1 of 2)	NFKBIA	ensembl biomart, HSA, GO:0002376
NFKBIA (2 of 2)	NFKBIA	ensembl biomart, HSA, GO:0002376
NHEJ1	NHEJ1	ensembl biomart, HSA, GO:0002376
NKAP	NKAP	ensembl biomart, HSA, GO:0002376
NKX2-3	NKX2-3	ensembl biomart, HSA, GO:0002376
NKX2-5	NKX2-5	ensembl biomart, HSA, GO:0002376
NKX3-2	NKX3-2	ensembl biomart, HSA, GO:0002376
NLRC5	NLRC5	ensembl biomart, HSA, GO:0002376
NLRX1	NLRX1	ensembl biomart, HSA, GO:0002376
NOD1	NOD1	ensembl biomart, HSA, GO:0002376
NOD2	NOD2	ensembl biomart, HSA, GO:0002376
NOTCH1 (1 of 2)	NOTCH1	ensembl biomart, HSA, GO:0002376
NOTCH1 (2 of 2)	NOTCH1	ensembl biomart, HSA, GO:0002376
NPEPPS	NPEPPS	ensembl biomart, HSA, GO:0002376
NTRK1	NTRK1	ensembl biomart, HSA, GO:0002376
NUB1	NUB1	ensembl biomart, HSA, GO:0002376
NUP85	NUP85	ensembl biomart, HSA, GO:0002376
ONECUT1	ONECUT1	ensembl biomart, HSA, GO:0002376
OPRD1 (1 of 2)	OPRD1	ensembl biomart, HSA, GO:0002376
OPRD1 (2 of 2)	OPRD1	ensembl biomart, HSA, GO:0002376
OPRK1 (1 of 2)	OPRK1	ensembl biomart, HSA, GO:0002376
OPRK1 (2 of 2)	OPRK1	ensembl biomart, HSA, GO:0002376
OSBPL1A (1 of 2)	OSBPL1A	ensembl biomart, HSA, GO:0002376
OSBPL1A (2 of 2)	OSBPL1A	ensembl biomart, HSA, GO:0002376
OSTM1	OSTM1	ensembl biomart, HSA, GO:0002376
OTUD5	OTUD5	ensembl biomart, HSA, GO:0002376
PAFAH1B1 (1 of 2)	PAFAH1B1	ensembl biomart, HSA, GO:0002376
PAFAH1B1 (2 of 2)	PAFAH1B1	ensembl biomart, HSA, GO:0002376
PAG1	PAG1	ensembl biomart, HSA, GO:0002376
PAK1 (1 of 2)	PAK1	ensembl biomart, HSA, GO:0002376
PAK1 (2 of 2)	PAK1	ensembl biomart, HSA, GO:0002376
PAK2 (1 of 2)	PAK2	ensembl biomart, HSA, GO:0002376
PAK2 (2 of 2)	PAK2	ensembl biomart, HSA, GO:0002376
PANX1 (1 of 2)	PANX1	ensembl biomart, HSA, GO:0002376
PANX1 (2 of 2)	PANX1	ensembl biomart, HSA, GO:0002376
PAX1	PAX1	ensembl biomart, HSA, GO:0002376
PAX5	PAX5	ensembl biomart, HSA, GO:0002376
PBX1 (1 of 2)	PBX1	ensembl biomart, HSA, GO:0002376
PBX1 (2 of 2)	PBX1	ensembl biomart, HSA, GO:0002376
PCID2 (1 of 2)	PCID2	ensembl biomart, HSA, GO:0002376
PCID2 (2 of 2)	PCID2	ensembl biomart, HSA, GO:0002376
PDE1B	PDE1B	ensembl biomart, HSA, GO:0002376
PDE2A	PDE2A	ensembl biomart, HSA, GO:0002376
PDE4B (2 of 2)	PDE4B	ensembl biomart, HSA, GO:0002376
PDE4D	PDE4D	ensembl biomart, HSA, GO:0002376
PDGFB (1 of 2)	PDGFB	ensembl biomart, HSA, GO:0002376
PDGFB (2 of 2)	PDGFB	ensembl biomart, HSA, GO:0002376
PDIA3	PDIA3	ensembl biomart, HSA, GO:0002376
PDPK1	PDPK1	ensembl biomart, HSA, GO:0002376
PFDN1	PFDN1	ensembl biomart, HSA, GO:0002376
PGLYRP2 (1 of 2)	PGLYRP2	ensembl biomart, HSA, GO:0002376
PGLYRP2 (2 of 2)	PGLYRP2	ensembl biomart, HSA, GO:0002376
PGM3	PGM3	ensembl biomart, HSA, GO:0002376
PI4K2A	PI4K2A	ensembl biomart, HSA, GO:0002376
PIAS1 (1 of 2)	PIAS1	ensembl biomart, HSA, GO:0002376
PIAS1 (2 of 2)	PIAS1	ensembl biomart, HSA, GO:0002376
PIK3C3	PIK3C3	ensembl biomart, HSA, GO:0002376
PIK3CB	PIK3CB	ensembl biomart, HSA, GO:0002376
PIK3CG	PIK3CG	ensembl biomart, HSA, GO:0002376
PIK3R1 (1 of 2)	PIK3R1	ensembl biomart, HSA, GO:0002376
PIK3R1 (2 of 2)	PIK3R1	ensembl biomart, HSA, GO:0002376
PIK3R2 (1 of 2)	PIK3R2	ensembl biomart, HSA, GO:0002376
PIK3R2 (2 of 2)	PIK3R2	ensembl biomart, HSA, GO:0002376
PIK3R3	PIK3R3	ensembl biomart, HSA, GO:0002376
PIK3R4	PIK3R4	ensembl biomart, HSA, GO:0002376
PIN1	PIN1	ensembl biomart, HSA, GO:0002376
PIP4K2A	PIP4K2A	ensembl biomart, HSA, GO:0002376
PIP5K1C (1 of 2)	PIP5K1C	ensembl biomart, HSA, GO:0002376
PIP5K1C (2 of 2)	PIP5K1C	ensembl biomart, HSA, GO:0002376
PITX2	PITX2	ensembl biomart, HSA, GO:0002376
PKHD1L1	PKHD1L1	ensembl biomart, HSA, GO:0002376
PKNOX1	PKNOX1	ensembl biomart, HSA, GO:0002376
PLEK	PLEK	ensembl biomart, HSA, GO:0002376
PLEKHA1 (1 of 2)	PLEKHA1	ensembl biomart, HSA, GO:0002376
PLEKHA1 (2 of 2)	PLEKHA1	ensembl biomart, HSA, GO:0002376
PLG	PLG	ensembl biomart, HSA, GO:0002376
PMS2	PMS2	ensembl biomart, HSA, GO:0002376
PODXL	PODXL	ensembl biomart, HSA, GO:0002376
PODXL2	PODXL2	ensembl biomart, HSA, GO:0002376
POU1F1	POU1F1	ensembl biomart, HSA, GO:0002376
POU2F2 (1 of 2)	POU2F2	ensembl biomart, HSA, GO:0002376
POU2F2 (2 of 2)	POU2F2	ensembl biomart, HSA, GO:0002376
PPARG	PPARG	ensembl biomart, HSA, GO:0002376
PRDX1	PRDX1	ensembl biomart, HSA, GO:0002376
PRDX3	PRDX3	ensembl biomart, HSA, GO:0002376
PRELID1	PRELID1	ensembl biomart, HSA, GO:0002376
PREX1	PREX1	ensembl biomart, HSA, GO:0002376
PRF1 (1 of 5)	PRF1	ensembl biomart, HSA, GO:0002376
PRF1 (2 of 5)	PRF1	ensembl biomart, HSA, GO:0002376
PRF1 (3 of 5)	PRF1	ensembl biomart, HSA, GO:0002376
PRF1 (4 of 5)	PRF1	ensembl biomart, HSA, GO:0002376
PRF1 (5 of 5)	PRF1	ensembl biomart, HSA, GO:0002376
PRG4	PRG4	ensembl biomart, HSA, GO:0002376
PRKCA (1 of 2)	PRKCA	ensembl biomart, HSA, GO:0002376
PRKCA (2 of 2)	PRKCA	ensembl biomart, HSA, GO:0002376
PRKCB (1 of 2)	PRKCB	ensembl biomart, HSA, GO:0002376
PRKCB (2 of 2)	PRKCB	ensembl biomart, HSA, GO:0002376
PRKCD (1 of 2)	PRKCD	ensembl biomart, HSA, GO:0002376
PRKCD (2 of 2)	PRKCD	ensembl biomart, HSA, GO:0002376
PRKCE (1 of 2)	PRKCE	ensembl biomart, HSA, GO:0002376
PRKCE (2 of 2)	PRKCE	ensembl biomart, HSA, GO:0002376
PRKCQ	PRKCQ	ensembl biomart, HSA, GO:0002376
PRKCSH	PRKCSH	ensembl biomart, HSA, GO:0002376
PRKD2	PRKD2	ensembl biomart, HSA, GO:0002376
PRKDC	PRKDC	ensembl biomart, HSA, GO:0002376
PRKRA	PRKRA	ensembl biomart, HSA, GO:0002376
PRKX	PRKX	ensembl biomart, HSA, GO:0002376
PRLR (1 of 2)	PRLR	ensembl biomart, HSA, GO:0002376
PRLR (2 of 2)	PRLR	ensembl biomart, HSA, GO:0002376
PSEN1	PSEN1	ensembl biomart, HSA, GO:0002376
PSMA4 (1 of 2)	PSMA4	ensembl biomart, HSA, GO:0002376
PSMA4 (2 of 2)	PSMA4	ensembl biomart, HSA, GO:0002376
PSMA6 (1 of 2)	PSMA6	ensembl biomart, HSA, GO:0002376
PSMA6 (2 of 2)	PSMA6	ensembl biomart, HSA, GO:0002376
PSMB8 (1 of 10)	PSMB8	ensembl biomart, HSA, GO:0002376
PSMB8 (10 of 10)	PSMB8	ensembl biomart, HSA, GO:0002376
PSMB8 (2 of 10)	PSMB8	ensembl biomart, HSA, GO:0002376
PSMB8 (3 of 10)	PSMB8	ensembl biomart, HSA, GO:0002376
PSMB8 (4 of 10)	PSMB8	ensembl biomart, HSA, GO:0002376
PSMB8 (5 of 10)	PSMB8	ensembl biomart, HSA, GO:0002376
PSMB8 (6 of 10)	PSMB8	ensembl biomart, HSA, GO:0002376
PSMB8 (7 of 10)	PSMB8	ensembl biomart, HSA, GO:0002376
PSMB8 (8 of 10)	PSMB8	ensembl biomart, HSA, GO:0002376
PSMB8 (9 of 10)	PSMB8	ensembl biomart, HSA, GO:0002376
PSMB9 (1 of 5)	PSMB9	ensembl biomart, HSA, GO:0002376
PSMB9 (2 of 5)	PSMB9	ensembl biomart, HSA, GO:0002376
PSMB9 (3 of 5)	PSMB9	ensembl biomart, HSA, GO:0002376
PSMB9 (4 of 5)	PSMB9	ensembl biomart, HSA, GO:0002376
PSMB9 (5 of 5)	PSMB9	ensembl biomart, HSA, GO:0002376
PSMD11 (1 of 2)	PSMD11	ensembl biomart, HSA, GO:0002376
PSMD11 (2 of 2)	PSMD11	ensembl biomart, HSA, GO:0002376
PSMD4 (2 of 2)	PSMD4	ensembl biomart, HSA, GO:0002376
PSME4 (1 of 2)	PSME4	ensembl biomart, HSA, GO:0002376
PSME4 (2 of 2)	PSME4	ensembl biomart, HSA, GO:0002376
PSTPIP1 (1 of 2)	PSTPIP1	ensembl biomart, HSA, GO:0002376
PSTPIP1 (2 of 2)	PSTPIP1	ensembl biomart, HSA, GO:0002376
PTAFR	PTAFR	ensembl biomart, HSA, GO:0002376
PTBP3	PTBP3	ensembl biomart, HSA, GO:0002376
PTEN (1 of 2)	PTEN	ensembl biomart, HSA, GO:0002376
PTEN (2 of 2)	PTEN	ensembl biomart, HSA, GO:0002376
PTGDR2 (1 of 3)	PTGDR2	ensembl biomart, HSA, GO:0002376
PTGDR2 (2 of 3)	PTGDR2	ensembl biomart, HSA, GO:0002376
PTGDR2 (3 of 3)	PTGDR2	ensembl biomart, HSA, GO:0002376
PTGER4 (1 of 2)	PTGER4	ensembl biomart, HSA, GO:0002376
PTGER4 (2 of 2)	PTGER4	ensembl biomart, HSA, GO:0002376
PTK2B (1 of 2)	PTK2B	ensembl biomart, HSA, GO:0002376
PTK2B (2 of 2)	PTK2B	ensembl biomart, HSA, GO:0002376
PTPN1	PTPN1	ensembl biomart, HSA, GO:0002376
PTPN11	PTPN11	ensembl biomart, HSA, GO:0002376
PTPN2 (1 of 2)	PTPN2	ensembl biomart, HSA, GO:0002376
PTPN2 (2 of 2)	PTPN2	ensembl biomart, HSA, GO:0002376
PTPN22	PTPN22	ensembl biomart, HSA, GO:0002376
PTPN6	PTPN6	ensembl biomart, HSA, GO:0002376
PTPRC	PTPRC	ensembl biomart, HSA, GO:0002376
PTPRO	PTPRO	ensembl biomart, HSA, GO:0002376
PTX3	PTX3	ensembl biomart, HSA, GO:0002376
PVRL1 (1 of 2)	PVRL1	ensembl biomart, HSA, GO:0002376
PVRL1 (2 of 2)	PVRL1	ensembl biomart, HSA, GO:0002376
RAB27A	RAB27A	ensembl biomart, HSA, GO:0002376
RAB7A (1 of 2)	RAB7A	ensembl biomart, HSA, GO:0002376
RAB7A (2 of 2)	RAB7A	ensembl biomart, HSA, GO:0002376
RAC1 (1 of 2)	RAC1	ensembl biomart, HSA, GO:0002376
RAC1 (2 of 2)	RAC1	ensembl biomart, HSA, GO:0002376
RACGAP1	RACGAP1	ensembl biomart, HSA, GO:0002376
RAG1	RAG1	ensembl biomart, HSA, GO:0002376
RAG2	RAG2	ensembl biomart, HSA, GO:0002376
RASGRP4	RASGRP4	ensembl biomart, HSA, GO:0002376
RB1	RB1	ensembl biomart, HSA, GO:0002376
RBCK1	RBCK1	ensembl biomart, HSA, GO:0002376
RBM15 (1 of 2)	RBM15	ensembl biomart, HSA, GO:0002376
RBM15 (2 of 2)	RBM15	ensembl biomart, HSA, GO:0002376
RELA	RELA	ensembl biomart, HSA, GO:0002376
RELB	RELB	ensembl biomart, HSA, GO:0002376
RET	RET	ensembl biomart, HSA, GO:0002376
RFX1 (1 of 2)	RFX1	ensembl biomart, HSA, GO:0002376
RFX1 (2 of 2)	RFX1	ensembl biomart, HSA, GO:0002376
RGCC	RGCC	ensembl biomart, HSA, GO:0002376
RGS1	RGS1	ensembl biomart, HSA, GO:0002376
RHOH	RHOH	ensembl biomart, HSA, GO:0002376
RICTOR	RICTOR	ensembl biomart, HSA, GO:0002376
RILP	RILP	ensembl biomart, HSA, GO:0002376
RIPK2	RIPK2	ensembl biomart, HSA, GO:0002376
RIPK3	RIPK3	ensembl biomart, HSA, GO:0002376
RNF168	RNF168	ensembl biomart, HSA, GO:0002376
RNF8	RNF8	ensembl biomart, HSA, GO:0002376
ROCK1	ROCK1	ensembl biomart, HSA, GO:0002376
RORC	RORC	ensembl biomart, HSA, GO:0002376
RPA1 (1 of 2)	RPA1	ensembl biomart, HSA, GO:0002376
RPA1 (2 of 2)	RPA1	ensembl biomart, HSA, GO:0002376
RPL22 (1 of 2)	RPL22	ensembl biomart, HSA, GO:0002376
RPL22 (2 of 2)	RPL22	ensembl biomart, HSA, GO:0002376
RPS14	RPS14	ensembl biomart, HSA, GO:0002376
RPS19	RPS19	ensembl biomart, HSA, GO:0002376
RPS27A	RPS27A	ensembl biomart, HSA, GO:0002376
RPS6KA1	RPS6KA1	ensembl biomart, HSA, GO:0002376
RPS6KA2	RPS6KA2	ensembl biomart, HSA, GO:0002376
RPS6KA3	RPS6KA3	ensembl biomart, HSA, GO:0002376
RPS6KA5	RPS6KA5	ensembl biomart, HSA, GO:0002376
RSAD2	RSAD2	ensembl biomart, HSA, GO:0002376
RTKN2	RTKN2	ensembl biomart, HSA, GO:0002376
RUNX1	RUNX1	ensembl biomart, HSA, GO:0002376
RUNX2	RUNX2	ensembl biomart, HSA, GO:0002376
S100B	S100B	ensembl biomart, HSA, GO:0002376
S1PR4	S1PR4	ensembl biomart, HSA, GO:0002376
SATB1	SATB1	ensembl biomart, HSA, GO:0002376
SBDS	SBDS	ensembl biomart, HSA, GO:0002376
SCG2 (1 of 2)	SCG2	ensembl biomart, HSA, GO:0002376
SCG2 (2 of 2)	SCG2	ensembl biomart, HSA, GO:0002376
SEC61A1 (1 of 2)	SEC61A1	ensembl biomart, HSA, GO:0002376
SEC61A1 (2 of 2)	SEC61A1	ensembl biomart, HSA, GO:0002376
SEC61B	SEC61B	ensembl biomart, HSA, GO:0002376
SEMA7A (1 of 2)	SEMA7A	ensembl biomart, HSA, GO:0002376
SEMA7A (2 of 2)	SEMA7A	ensembl biomart, HSA, GO:0002376
SERPING1	SERPING1	ensembl biomart, HSA, GO:0002376
SFRP1	SFRP1	ensembl biomart, HSA, GO:0002376
SFRP2	SFRP2	ensembl biomart, HSA, GO:0002376
SFXN1	SFXN1	ensembl biomart, HSA, GO:0002376
SGPL1	SGPL1	ensembl biomart, HSA, GO:0002376
SH2B2	SH2B2	ensembl biomart, HSA, GO:0002376
SH2B3	SH2B3	ensembl biomart, HSA, GO:0002376
SH2D1A	SH2D1A	ensembl biomart, HSA, GO:0002376
SH2D1B	SH2D1B	ensembl biomart, HSA, GO:0002376
SH3GL2 (1 of 2)	SH3GL2	ensembl biomart, HSA, GO:0002376
SH3GL2 (2 of 2)	SH3GL2	ensembl biomart, HSA, GO:0002376
SHH	SHH	ensembl biomart, HSA, GO:0002376
SIGIRR	SIGIRR	ensembl biomart, HSA, GO:0002376
SIKE1	SIKE1	ensembl biomart, HSA, GO:0002376
SIX1	SIX1	ensembl biomart, HSA, GO:0002376
SIX4	SIX4	ensembl biomart, HSA, GO:0002376
SKAP1	SKAP1	ensembl biomart, HSA, GO:0002376
SKAP2	SKAP2	ensembl biomart, HSA, GO:0002376
SKIL	SKIL	ensembl biomart, HSA, GO:0002376
SLC11A2 (1 of 2)	SLC11A2	ensembl biomart, HSA, GO:0002376
SLC11A2 (2 of 2)	SLC11A2	ensembl biomart, HSA, GO:0002376
SLC16A1 (1 of 2)	SLC16A1	ensembl biomart, HSA, GO:0002376
SLC16A1 (2 of 2)	SLC16A1	ensembl biomart, HSA, GO:0002376
SLC16A3 (1 of 2)	SLC16A3	ensembl biomart, HSA, GO:0002376
SLC16A3 (2 of 2)	SLC16A3	ensembl biomart, HSA, GO:0002376
SLC25A38 (1 of 2)	SLC25A38	ensembl biomart, HSA, GO:0002376
SLC25A38 (2 of 2)	SLC25A38	ensembl biomart, HSA, GO:0002376
SLC3A2 (1 of 2)	SLC3A2	ensembl biomart, HSA, GO:0002376
SLC3A2 (2 of 2)	SLC3A2	ensembl biomart, HSA, GO:0002376
SLC7A10 (1 of 2)	SLC7A10	ensembl biomart, HSA, GO:0002376
SLC7A10 (2 of 2)	SLC7A10	ensembl biomart, HSA, GO:0002376
SLC7A9 (1 of 2)	SLC7A9	ensembl biomart, HSA, GO:0002376
SLC7A9 (2 of 2)	SLC7A9	ensembl biomart, HSA, GO:0002376
SMAD3	SMAD3	ensembl biomart, HSA, GO:0002376
SMAD6	SMAD6	ensembl biomart, HSA, GO:0002376
SMARCA4	SMARCA4	ensembl biomart, HSA, GO:0002376
SNRK	SNRK	ensembl biomart, HSA, GO:0002376
SOCS1 (1 of 2)	SOCS1	ensembl biomart, HSA, GO:0002376
SOCS1 (2 of 2)	SOCS1	ensembl biomart, HSA, GO:0002376
SOCS3 (1 of 2)	SOCS3	ensembl biomart, HSA, GO:0002376
SOCS3 (2 of 2)	SOCS3	ensembl biomart, HSA, GO:0002376
SOD1	SOD1	ensembl biomart, HSA, GO:0002376
SOD2	SOD2	ensembl biomart, HSA, GO:0002376
SOX4 (1 of 2)	SOX4	ensembl biomart, HSA, GO:0002376
SOX4 (2 of 2)	SOX4	ensembl biomart, HSA, GO:0002376
SP1	SP1	ensembl biomart, HSA, GO:0002376
SP2	SP2	ensembl biomart, HSA, GO:0002376
SP3 (1 of 2)	SP3	ensembl biomart, HSA, GO:0002376
SP3 (2 of 2)	SP3	ensembl biomart, HSA, GO:0002376
SP7	SP7	ensembl biomart, HSA, GO:0002376
SPI1 (1 of 2)	SPI1	ensembl biomart, HSA, GO:0002376
SPI1 (2 of 2)	SPI1	ensembl biomart, HSA, GO:0002376
SPON2	SPON2	ensembl biomart, HSA, GO:0002376
SPTB	SPTB	ensembl biomart, HSA, GO:0002376
SPTBN2	SPTBN2	ensembl biomart, HSA, GO:0002376
SRC (1 of 2)	SRC	ensembl biomart, HSA, GO:0002376
SRC (2 of 2)	SRC	ensembl biomart, HSA, GO:0002376
SRF (1 of 2)	SRF	ensembl biomart, HSA, GO:0002376
SRF (2 of 2)	SRF	ensembl biomart, HSA, GO:0002376
SRPK1 (1 of 2)	SRPK1	ensembl biomart, HSA, GO:0002376
SRPK1 (2 of 2)	SRPK1	ensembl biomart, HSA, GO:0002376
SRPK2	SRPK2	ensembl biomart, HSA, GO:0002376
ST6GAL1	ST6GAL1	ensembl biomart, HSA, GO:0002376
STAR (1 of 2)	STAR	ensembl biomart, HSA, GO:0002376
STAR (2 of 2)	STAR	ensembl biomart, HSA, GO:0002376
STAT1	STAT1	ensembl biomart, HSA, GO:0002376
STK3	STK3	ensembl biomart, HSA, GO:0002376
STK4	STK4	ensembl biomart, HSA, GO:0002376
STXBP2	STXBP2	ensembl biomart, HSA, GO:0002376
STXBP3	STXBP3	ensembl biomart, HSA, GO:0002376
SUMO1	SUMO1	ensembl biomart, HSA, GO:0002376
SUSD2	SUSD2	ensembl biomart, HSA, GO:0002376
SWAP70 (1 of 2)	SWAP70	ensembl biomart, HSA, GO:0002376
SWAP70 (2 of 2)	SWAP70	ensembl biomart, HSA, GO:0002376
SYK	SYK	ensembl biomart, HSA, GO:0002376
TAB2	TAB2	ensembl biomart, HSA, GO:0002376
TAB3	TAB3	ensembl biomart, HSA, GO:0002376
TACC3	TACC3	ensembl biomart, HSA, GO:0002376
TAL1	TAL1	ensembl biomart, HSA, GO:0002376
TANK	TANK	ensembl biomart, HSA, GO:0002376
TAP1	TAP1	ensembl biomart, HSA, GO:0002376
TAPBP (1 of 7)	TAPBP	ensembl biomart, HSA, GO:0002376
TAPBP (2 of 7)	TAPBP	ensembl biomart, HSA, GO:0002376
TAPBP (3 of 7)	TAPBP	ensembl biomart, HSA, GO:0002376
TAPBP (4 of 7)	TAPBP	ensembl biomart, HSA, GO:0002376
TAPBP (5 of 7)	TAPBP	ensembl biomart, HSA, GO:0002376
TAPBPL	TAPBPL	ensembl biomart, HSA, GO:0002376
TAX1BP1 (1 of 2)	TAX1BP1	ensembl biomart, HSA, GO:0002376
TAX1BP1 (2 of 2)	TAX1BP1	ensembl biomart, HSA, GO:0002376
TAZ	TAZ	ensembl biomart, HSA, GO:0002376
TBK1	TBK1	ensembl biomart, HSA, GO:0002376
TBKBP1 (1 of 2)	TBKBP1	ensembl biomart, HSA, GO:0002376
TBKBP1 (2 of 2)	TBKBP1	ensembl biomart, HSA, GO:0002376
TBX1	TBX1	ensembl biomart, HSA, GO:0002376
TCF12	TCF12	ensembl biomart, HSA, GO:0002376
TCF21	TCF21	ensembl biomart, HSA, GO:0002376
TCF3 (1 of 2)	TCF3	ensembl biomart, HSA, GO:0002376
TCF3 (2 of 2)	TCF3	ensembl biomart, HSA, GO:0002376
TEC	TEC	ensembl biomart, HSA, GO:0002376
TESC (1 of 2)	TESC	ensembl biomart, HSA, GO:0002376
TESC (2 of 2)	TESC	ensembl biomart, HSA, GO:0002376
TET2	TET2	ensembl biomart, HSA, GO:0002376
TFEB	TFEB	ensembl biomart, HSA, GO:0002376
TFRC (1 of 2)	TFRC	ensembl biomart, HSA, GO:0002376
TFRC (2 of 2)	TFRC	ensembl biomart, HSA, GO:0002376
TGFB1	TGFB1	ensembl biomart, HSA, GO:0002376
TGFB2	TGFB2	ensembl biomart, HSA, GO:0002376
TGFBR1	TGFBR1	ensembl biomart, HSA, GO:0002376
TGFBR2 (1 of 2)	TGFBR2	ensembl biomart, HSA, GO:0002376
TGFBR2 (2 of 2)	TGFBR2	ensembl biomart, HSA, GO:0002376
TGFBR3	TGFBR3	ensembl biomart, HSA, GO:0002376
THBD	THBD	ensembl biomart, HSA, GO:0002376
THBS1 (1 of 2)	THBS1	ensembl biomart, HSA, GO:0002376
THBS1 (2 of 2)	THBS1	ensembl biomart, HSA, GO:0002376
THOC5	THOC5	ensembl biomart, HSA, GO:0002376
THRA (1 of 2)	THRA	ensembl biomart, HSA, GO:0002376
THRA (2 of 2)	THRA	ensembl biomart, HSA, GO:0002376
TINAGL1	TINAGL1	ensembl biomart, HSA, GO:0002376
TLR2	TLR2	ensembl biomart, HSA, GO:0002376
TLR5 (1 of 3)	TLR5	ensembl biomart, HSA, GO:0002376
TLR5 (2 of 3)	TLR5	ensembl biomart, HSA, GO:0002376
TLR5 (3 of 3)	TLR5	ensembl biomart, HSA, GO:0002376
TLR7	TLR7	ensembl biomart, HSA, GO:0002376
TLR9	TLR9	ensembl biomart, HSA, GO:0002376
TLX1	TLX1	ensembl biomart, HSA, GO:0002376
TMEM173	TMEM173	ensembl biomart, HSA, GO:0002376
TMX1	TMX1	ensembl biomart, HSA, GO:0002376
TNFAIP1	TNFAIP1	ensembl biomart, HSA, GO:0002376
TNFAIP3	TNFAIP3	ensembl biomart, HSA, GO:0002376
TNFAIP8L2	TNFAIP8L2	ensembl biomart, HSA, GO:0002376
TNFRSF14	TNFRSF14	ensembl biomart, HSA, GO:0002376
TNFRSF1B	TNFRSF1B	ensembl biomart, HSA, GO:0002376
TNIP1	TNIP1	ensembl biomart, HSA, GO:0002376
TNIP2	TNIP2	ensembl biomart, HSA, GO:0002376
TNRC6C (1 of 2)	TNRC6C	ensembl biomart, HSA, GO:0002376
TNRC6C (2 of 2)	TNRC6C	ensembl biomart, HSA, GO:0002376
TP53	TP53	ensembl biomart, HSA, GO:0002376
TPD52	TPD52	ensembl biomart, HSA, GO:0002376
TPO	TPO	ensembl biomart, HSA, GO:0002376
TPP2	TPP2	ensembl biomart, HSA, GO:0002376
TRAF2 (1 of 2)	TRAF2	ensembl biomart, HSA, GO:0002376
TRAF2 (2 of 2)	TRAF2	ensembl biomart, HSA, GO:0002376
TRAF3IP2	TRAF3IP2	ensembl biomart, HSA, GO:0002376
TRAF6	TRAF6	ensembl biomart, HSA, GO:0002376
TRIM25 (1 of 2)	TRIM25	ensembl biomart, HSA, GO:0002376
TRIM25 (2 of 2)	TRIM25	ensembl biomart, HSA, GO:0002376
TRIM32	TRIM32	ensembl biomart, HSA, GO:0002376
TRPM4 (1 of 2)	TRPM4	ensembl biomart, HSA, GO:0002376
TRPM4 (2 of 2)	TRPM4	ensembl biomart, HSA, GO:0002376
TSHR (1 of 2)	TSHR	ensembl biomart, HSA, GO:0002376
TSHR (2 of 2)	TSHR	ensembl biomart, HSA, GO:0002376
TTC7A	TTC7A	ensembl biomart, HSA, GO:0002376
TUBB	TUBB	ensembl biomart, HSA, GO:0002376
TUSC2 (1 of 2)	TUSC2	ensembl biomart, HSA, GO:0002376
TUSC2 (2 of 2)	TUSC2	ensembl biomart, HSA, GO:0002376
TXK	TXK	ensembl biomart, HSA, GO:0002376
TXNIP	TXNIP	ensembl biomart, HSA, GO:0002376
TXNRD2	TXNRD2	ensembl biomart, HSA, GO:0002376
TYK2	TYK2	ensembl biomart, HSA, GO:0002376
TYR (1 of 2)	TYR	ensembl biomart, HSA, GO:0002376
TYR (2 of 2)	TYR	ensembl biomart, HSA, GO:0002376
TYRO3	TYRO3	ensembl biomart, HSA, GO:0002376
UBA52	UBA52	ensembl biomart, HSA, GO:0002376
UBA7	UBA7	ensembl biomart, HSA, GO:0002376
UBE2A	UBE2A	ensembl biomart, HSA, GO:0002376
UNG	UNG	ensembl biomart, HSA, GO:0002376
USP18	USP18	ensembl biomart, HSA, GO:0002376
VAMP7	VAMP7	ensembl biomart, HSA, GO:0002376
VASP	VASP	ensembl biomart, HSA, GO:0002376
VAV1	VAV1	ensembl biomart, HSA, GO:0002376
VAV3 (1 of 2)	VAV3	ensembl biomart, HSA, GO:0002376
VAV3 (2 of 2)	VAV3	ensembl biomart, HSA, GO:0002376
VCAM1	VCAM1	ensembl biomart, HSA, GO:0002376
VEGFA (1 of 2)	VEGFA	ensembl biomart, HSA, GO:0002376
VEGFA (2 of 2)	VEGFA	ensembl biomart, HSA, GO:0002376
VIPR1 (1 of 2)	VIPR1	ensembl biomart, HSA, GO:0002376
VIPR1 (2 of 2)	VIPR1	ensembl biomart, HSA, GO:0002376
VPS33A	VPS33A	ensembl biomart, HSA, GO:0002376
VTN (1 of 2)	VTN	ensembl biomart, HSA, GO:0002376
VTN (2 of 2)	VTN	ensembl biomart, HSA, GO:0002376
WAS	WAS	ensembl biomart, HSA, GO:0002376
WNT1	WNT1	ensembl biomart, HSA, GO:0002376
WNT10B	WNT10B	ensembl biomart, HSA, GO:0002376
WNT2B (1 of 2)	WNT2B	ensembl biomart, HSA, GO:0002376
WNT2B (2 of 2)	WNT2B	ensembl biomart, HSA, GO:0002376
WNT3	WNT3	ensembl biomart, HSA, GO:0002376
WNT4	WNT4	ensembl biomart, HSA, GO:0002376
WNT5A	WNT5A	ensembl biomart, HSA, GO:0002376
XBP1	XBP1	ensembl biomart, HSA, GO:0002376
XRCC5	XRCC5	ensembl biomart, HSA, GO:0002376
XRCC6	XRCC6	ensembl biomart, HSA, GO:0002376
YES1 (1 of 2)	YES1	ensembl biomart, HSA, GO:0002376
YES1 (2 of 2)	YES1	ensembl biomart, HSA, GO:0002376
YTHDF2	YTHDF2	ensembl biomart, HSA, GO:0002376
ZBTB16	ZBTB16	ensembl biomart, HSA, GO:0002376
ZC3H8	ZC3H8	ensembl biomart, HSA, GO:0002376
ZC3HAV1	ZC3HAV1	ensembl biomart, HSA, GO:0002376
ZEB1 (1 of 2)	ZEB1	ensembl biomart, HSA, GO:0002376
ZEB1 (2 of 2)	ZEB1	ensembl biomart, HSA, GO:0002376
ZFAT	ZFAT	ensembl biomart, HSA, GO:0002376
ZFP36L1	ZFP36L1	ensembl biomart, HSA, GO:0002376
ZFPM1	ZFPM1	ensembl biomart, HSA, GO:0002376
C1QTNF4 (1 of 2)	C1QTNF4	Star et al. 2011; Haase et al. 2013
C1QTNF4 (2 of 2)	C1QTNF4	Star et al. 2011; Haase et al. 2013
C1QTNF5	C1QTNF5	Star et al. 2011; Haase et al. 2013
C4A	C4	Star et al. 2011; Haase et al. 2013
CCR7	CCR7	Star et al. 2011; Haase et al. 2013
CD226	CD226	Star et al. 2011; Haase et al. 2013
CD3E	CD3E	Star et al. 2011; Haase et al. 2013
CD74 (2 of 2)	CD74	Star et al. 2011; Haase et al. 2013
CD79A	CD79A	Star et al. 2011; Haase et al. 2013
CD79B	CD79B	Star et al. 2011; Haase et al. 2013
CXCR3	CXCR3	Star et al. 2011; Haase et al. 2013
ENSGACG00000000336	MHC II beta	Star et al. 2011; Haase et al. 2013
ENSGACG00000000478	TNF	Star et al. 2011; Haase et al. 2013
ENSGACG00000000569	IgM	Star et al. 2011; Haase et al. 2013
ENSGACG00000001729	IL8	Star et al. 2011; Haase et al. 2013
ENSGACG00000001745	TLR18	Star et al. 2011; Haase et al. 2013
ENSGACG00000001805	TRGC	Star et al. 2011; Haase et al. 2013
ENSGACG00000001919	MHC I	Star et al. 2011; Haase et al. 2013
ENSGACG00000003030	C1QTNF3	Star et al. 2011; Haase et al. 2013
ENSGACG00000005289	TRBC	Star et al. 2011; Haase et al. 2013
ENSGACG00000005449	TLR22	Star et al. 2011; Haase et al. 2013
ENSGACG00000008369	AIRE	Star et al. 2011; Haase et al. 2013
ENSGACG00000008375	AIRE	Star et al. 2011; Haase et al. 2013
ENSGACG00000008397	TLR13	Star et al. 2011; Haase et al. 2013
ENSGACG00000008945	CD8A	Star et al. 2011; Haase et al. 2013
ENSGACG00000008960	CD8B	Star et al. 2011; Haase et al. 2013
ENSGACG00000009282	Fas	Star et al. 2011; Haase et al. 2013
ENSGACG00000009364	TLR21	Star et al. 2011; Haase et al. 2013
ENSGACG00000009832	CD4	Star et al. 2011; Haase et al. 2013
ENSGACG00000012763	IgM	Star et al. 2011; Haase et al. 2013
ENSGACG00000012766	IgM	Star et al. 2011; Haase et al. 2013
ENSGACG00000012767	IgD	Star et al. 2011; Haase et al. 2013
ENSGACG00000012769	IgM	Star et al. 2011; Haase et al. 2013
ENSGACG00000012781	IgD	Star et al. 2011; Haase et al. 2013
ENSGACG00000012783	IgM	Star et al. 2011; Haase et al. 2013
ENSGACG00000012792	IgM	Star et al. 2011; Haase et al. 2013
ENSGACG00000012797	IgD	Star et al. 2011; Haase et al. 2013
ENSGACG00000012799	IgM	Star et al. 2011; Haase et al. 2013
ENSGACG00000012804	IgM	Star et al. 2011; Haase et al. 2013
ENSGACG00000012809	IgM	Star et al. 2011; Haase et al. 2013
ENSGACG00000013031	GZMB	Star et al. 2011; Haase et al. 2013
ENSGACG00000013372	TNF	Star et al. 2011; Haase et al. 2013
ENSGACG00000014811	C5	Star et al. 2011; Haase et al. 2013
ENSGACG00000014852	C1QTNF3	Star et al. 2011; Haase et al. 2013
ENSGACG00000015763	TRGC	Star et al. 2011; Haase et al. 2013
ENSGACG00000016298	IL4RA	Star et al. 2011; Haase et al. 2013
ENSGACG00000017257	FasL	Star et al. 2011; Haase et al. 2013
ENSGACG00000017271	IL15	Star et al. 2011; Haase et al. 2013
ENSGACG00000019078	IL8	Star et al. 2011; Haase et al. 2013
ENSGACG00000019282	B2M	Star et al. 2011; Haase et al. 2013
ENSGACG00000019287	B2M	Star et al. 2011; Haase et al. 2013
FOXP3	FOXP3	Star et al. 2011; Haase et al. 2013
HLA-DMA (5 of 5)	MHC II alpha	Star et al. 2011; Haase et al. 2013
IGBP1	IGBP1	Star et al. 2011; Haase et al. 2013
IL12B (1 of 3)	IL12B	Star et al. 2011; Haase et al. 2013
IL12B (2 of 3)	IL12B	Star et al. 2011; Haase et al. 2013
IL12RB2	IL12RB2	Star et al. 2011; Haase et al. 2013
IL17D (1 of 2)	IL17D	Star et al. 2011; Haase et al. 2013
IL17D (2 of 2)	IL17D	Star et al. 2011; Haase et al. 2013
IL17RA (1 of 2)	IL17RA	Star et al. 2011; Haase et al. 2013
IL17RA (2 of 2)	IL17RA	Star et al. 2011; Haase et al. 2013
IL17RD	IL17RD	Star et al. 2011; Haase et al. 2013
IL1B	IL1B	Star et al. 2011; Haase et al. 2013
IL2RB	IL2RB	Star et al. 2011; Haase et al. 2013
IL6ST	IL6ST	Star et al. 2011; Haase et al. 2013
PSMB1	PSMB1	Star et al. 2011; Haase et al. 2013
PSMB10	PSMB10	Star et al. 2011; Haase et al. 2013
PSMB2	PSMB2	Star et al. 2011; Haase et al. 2013
PSMB3	PSMB3	Star et al. 2011; Haase et al. 2013
PSMB4	PSMB4	Star et al. 2011; Haase et al. 2013
PSMB5	PSMB5	Star et al. 2011; Haase et al. 2013
PSMB6	PSMB6	Star et al. 2011; Haase et al. 2013
PSMB7	PSMB7	Star et al. 2011; Haase et al. 2013
PSME1	PSME1	Star et al. 2011; Haase et al. 2013
PSME2	PSME2	Star et al. 2011; Haase et al. 2013
PSME3	PSME3	Star et al. 2011; Haase et al. 2013
RFX7 (1 of 2)	RFX7	Star et al. 2011; Haase et al. 2013
RFX7 (2 of 2)	RFX7	Star et al. 2011; Haase et al. 2013
RFXANK	RFXANK	Star et al. 2011; Haase et al. 2013
RFXAP	RFXAP	Star et al. 2011; Haase et al. 2013
TAPBP (6 of 7)	TAPBP	Star et al. 2011; Haase et al. 2013
TAPBP (7 of 7)	TAPBP	Star et al. 2011; Haase et al. 2013
TLR6	TLR1	Star et al. 2011; Haase et al. 2013
TLR8	TLR8	Star et al. 2011; Haase et al. 2013
UNC93B1	UNC93B	Star et al. 2011; Haase et al. 2013
